# What does a good day look like?: An interpretable machine learning approach to the American Time Use Survey

**DOI:** 10.1093/pnasnexus/pgag014

**Published:** 2026-03-13

**Authors:** Dunigan Folk, Mirka Henninger, Elizabeth Dunn

**Affiliations:** Department of Psychology, University of British Columbia, Vancouver, Canada V6T 1Z4; Faculty of Psychology, University of Basel, CH-4001 Basel, Switzerland; Department of Psychology, University of British Columbia, Vancouver, Canada V6T 1Z4

**Keywords:** time use, happiness, well-being, machine learning, random forests

## Abstract

What differentiates a happy day from a typical one? Using interpretable machine learning techniques, we assessed the relationship between the time people spent on over 100 activities and whether they rated their day as typical or “better than typical” in the 2013 (*n* = 9,286) and 2021 (*n* = 6,196) waves of the American Time Use Survey. Our random forest models identified better than typical (vs. typical) days with accuracy levels substantially above chance (i.e. 62–63% balanced accuracy across 2013 and 2021). Socializing was one of the activities most strongly linked to the probability of having a good day, but beyond 2 hours, additional socializing was not associated with further increases in the probability of reporting a better than typical day. Working for up to 6 hours was not related to whether people rated their day as better than usual; beyond 6 hours, however, additional work was associated with sharp declines in the probability of having a good day. While the present results are descriptive in nature, they provide insight into the rhythms and routines that characterize happy days.

Significance Statement We identified the differences between a good day and a typical one. Specifically, we used machine learning to assess how the amount of time people devoted to over one hundred activities related to whether they rated their day as better than typical. For some activities (e.g. time with friends), more time was almost always positively associated with having a good day. In contrast, some activities had a more complex relationship; working was associated with a decreased likelihood of having a good day, but only when people worked for more than 6 hours. While the present results are not causal in nature, they highlight a pattern of activities that differentiate better days from typical ones.

## Introduction

The novelist Annie Dillard once said, “How we spend our days is, of course, how we spend our lives.” By understanding how happy days differ from average ones, we can better understand the activities that comprise a happy life. Yet, little is known about the behavioral ingredients that commonly accompany good days. To investigate the common ingredients of good days, we used machine learning to assess how time spent on over 100 activities was associated with whether someone rated their day as better than typical or just average.

Researchers have identified a number of activities that are associated with greater happiness, such as socializing (1–4), spending time with friends and family (5, 6), and engaging in active leisure such as exercise (5, 7). In contrast, other activities, such as commuting or working are linked to feeling less happy (1, 8–10). Thus, past research points to a variety of activities that may characterize especially good days.

Of course, there are only 24 hours in a day, so minutes spent on one activity are minutes that cannot be spent on another. Even though socializing is associated with happiness (1–4), a good day will not necessarily include hours of conversation, partly because such time investment leaves less time for other activities. Yet, we know very little about how much time people devote to certain activities on days they rate as better than typical. Some activities may be akin to salt in a cookie recipe, occurring only in small amounts on most good days, while others may be more like chocolate chips, typically occurring in larger doses. Past research has investigated whether there are diminishing marginal returns to activities like socializing (11–13) and whether activities like working and commuting are especially unpleasant in high doses (14, 15). However, these studies examined specific activities in isolation, rather than assessing how a broader pattern of activities relates to having a good day. By examining a comprehensive list of daily activities, we aim to describe the most common ingredients of days that people rate as better than typical.

In the present study, we fit conditional random forest models (16) to two datasets from the 2013 (*n* = 9,286) and 2021 (*n* = 6,196) waves of the American Time Use Survey (ATUS). Specifically, we used the time people reported spending on over 100 activities to predict the probability that they rated their day as “better” than a typical day vs. the “same” as a typical day (we use the terms “better day,” “good day,” and “better-than-typical day” interchangeably throughout this manuscript). Compared with standard regression models, such as logistic regressions, random forests can model nonlinear relationships, complex interactions, and test many predictors simultaneously while avoiding overfitting (17). We also utilized interpretable machine learning techniques (18, 19) to elucidate the relationships between the time devoted to each activity and the probability that someone had a good day. By harnessing the power of interpretable machine learning, the present research provides the most comprehensive picture to date of what a good day looks like.

## Methods

We preregistered the sample, analysis plan, and exclusion criteria for this study (see https://tinyurl.com/3anmnu74).

### Sample

#### The American Time Use Survey

We utilized the 2013 and 2021 ATUS datasets available from the American Bureau of Labor Statistics (https://www.bls.gov/tus/). Specifically, we obtained the data on the amount of time participants spent doing each activity from the ATUS Activity Summary Files for 2013 and 2021. We then merged these datasets with the corresponding ATUS Well-Being Module Datasets, which contained participants’ responses to the question, “Thinking about yesterday as a whole, how would you say that your feelings, both good and bad, compared to a typical [fill day of the week]? Were they better than a typical [fill day of the week], the same as a typical [fill day of the week], or worse than a typical [fill day of the week]?”

When conducting the ATUS, the United States Census Bureau selects a broad range of American homes that collectively mirror the nation's demographic makeup. To ensure adequate data collection from specific populations, they then deliberately include a higher proportion of families that are Hispanic, Black, or have children in their household. Finally, the Census Bureau selects one individual who is at least 15 years old from each selected home to take part in the telephone survey. The Census Bureau equally samples weekends and weekdays, such that Saturday and Sundays represent 50% of the reported days. During the survey, participants reconstruct their prior 24 h in “episodes” from 4 AM the previous day to 4 AM the day of the interview, similar to the Day Reconstruction Methodology (1). It is worth noting that this definition of the “previous day” means that the reported sleep time captures parts of two different nights of sleep, rather than one complete night's rest. Full details on the ATUS methodology are available at https://www.bls.gov/tus/.

#### Final sample details

Because we were interested in what differentiated a good day from a typical day, we only included participants who responded with “better” or “the same” (as preregistered), leaving us with final samples of *n* = 9,286 (2013; mean age = 48.23, SD = 18.03; 55% female; 79% White, 15% Black, 4% Asian, 2% other; 70% typical days) and *n* = 6,196 (2021; mean age *=* 51.85, SD = 18.40, 53% female; 80% White, 12% Black, 5% Asian, 3% other; 71% typical days). We preregistered analyses involving the 2013 and 2021 datasets because these were the two most recent waves of the ATUS that included the question about whether participants had a good day.

### Activity categorization

After participants describe how they spent their day, ATUS coders categorize each episode into prespecified activity categories. As a result, the ATUS datasets contain several hundred numeric “Activity” variables that represent the number of minutes participants reported spending on a given behavior over the past 24 hours. All these variables are coded with a six-digit number (e.g. 010201 represents “Washing, dressing, and grooming oneself”), based on a prespecified coding scheme (see the ATUS documentation for more details: https://tinyurl.com/9799hmwy). As preregistered, we grouped the activities based on the first “four” digits of their numeric indicator (i.e. the “detailed subcategory” level of activity), resulting in ∼100 different activity variables. It is important to note that participants' descriptions of how they spent their time are coded based on the “primary” activity they were engaged in (e.g. each episode reported by participants is only assigned to a single activity category). As a result, the ATUS likely systematically underestimates the time participants spent on certain activities, given that each episode can only be categorized into a single activity.

Beyond the specific activity categories, we also included 8 additional broader time-use variables that were created by ATUS coders by combining other activity variables (e.g. TRTFAMILY = time spent with family, TRTFRIEND = time spent with friends; see our preregistration at https://tinyurl.com/3anmnu74 for the full list of the eight additional variables). These variables were created by combining all activities where participants indicated they were with the person of interest (i.e. TRTFAMILY combines the time spent on any activity where participants indicated a family member was present). In contrast to the 100+ activities which were mutually exclusive, it is possible for one single activity to count towards both time with friends and time with family (assuming friends and family were present).

### Analyses

We conducted the same set of analyses on both the 2013 and 2021 datasets. Below, we provide the full details on how we fit the models, determined variable importance, and investigated how the time spent on any given behavior related to the probability of having a good day. Our analysis plan was preregistered for both years (see https://tinyurl.com/3anmnu74).

#### Model fitting

We fit a conditional random forest classification model with each of the aforementioned time-use variables as predictors and whether participants rated their day “better” or the “same” as typical as the outcome variable. Note that across both years, the outcome variable was imbalanced, with far more people reporting their day was the same as typical (70% in 2013 and 71% in 2021) than those reporting that their day was better than typical. Such imbalanced data can lead machine learning models to overpredict the majority case (in our situation, typical days) because always predicting the majority case artificially leads to higher model accuracy than trying to accurately predict both cases. However, such a model would teach us nothing about the underlying differences between a good day and a typical day. As a result, we “downsampled” when fitting the models—a common approach to dealing with imbalanced outcome data (e.g. More and Rana (20)).

We fit the model on a random sample of 80% of the full datasets (i.e. the training set) in *R*; we used the train function from the “caret” package (21) to implement the cforest function from the “partykit” package (22). We tuned the mtry value (the number of features chosen at each split in the decision trees in the random forest) such that models with mtry values of 5, 10, 15, and 20 were fit. For model training and evaluation, we utilized out of bag (OOB) validation, a technique inherent to ensemble learning methods such as conditional random forests. For each tree in the ensemble, a subset of 75% of the training data was randomly selected without replacement to train the tree. The data points that are excluded from the training subset serve as the OOB data, which are used to validate the performance of that specific tree. The overall OOB accuracy is then computed by aggregating predictions for the excluded observations across all trees in the ensemble. As such, the model with the best OOB accuracy was chosen. This approach provides a robust and unbiased estimate of model accuracy.

After identifying the model with the mtry value that resulted in the best fit on the training set (2013: mtry = 5; 2021: mtry = 15), we evaluated the model performance using a test set, which consisted of the remaining 20% of the dataset that was not used in the training set. To evaluate fit, we assessed the overall and balanced accuracy estimates, the kappa estimate, and the confusion matrix using the test set with a classification threshold of 0.50.

It is important to underscore that the test set was imbalanced, such that there were more typical days than good days, as in the original structure of the data before we downsampled. The downsampling procedure ensured that the subset samples used for fitting each individual tree in the random forest contained an equal distribution of both “same” and “better” outcome cases. As mentioned, this prevents the model from simply maximizing accuracy by over-optimizing to fit the majority outcome (e.g. always predicting “typical” days because they are most frequent in the data). This was especially important for us, as we wanted to maximize the model's accuracy at predicting the “better” than typical days, rather than optimizing for predicting the typical days.

#### Variable importance

To identify the most important activities in predicting whether someone had a better than typical day, we calculated both the conditional and unconditional importance values for the predictor variables in the model we obtained from the model fitting steps described above. Specifically, we used the varimp function from the “partykit” package to obtain the conditional and unconditional importance values for the predictors in the model based on the training set. “Unconditional” importance values represent the impact of each variable in predicting the outcome without taking into consideration the impact of the other variables (akin to zero-order correlation; 23). “Conditional” importance values are similar to regression coefficients, as they represent the predictive utility of a variable over and above the impact of the other variables in the model (24, 25).

To narrow down our list of important variables to investigate further, we utilized a heuristic put forth by Strobl et al. (17). This heuristic takes advantage of the fact that unimportant variables may sometimes receive importance values less than zero, simply due to sampling error. As a result, variables that have positive conditional/unconditional importance values that are greater than the absolute value of the most negative conditional/unconditional importance values can be viewed as “important,” because they have an importance value higher than what one could expect due to sampling error alone. We preregistered that we would primarily focus on activities that passed this threshold with both their unconditional and conditional importance values, but that we would also examine variables that passed this threshold for either the unconditional or conditional variables. We provide the raw conditional importance values for the 20 most important variables in Supplementary material. However, we decided to focus on the unconditional importance values because we were interested in the activities that were most descriptively associated with having a good day (i.e. akin to raw correlations), rather than those with the largest partial effect. That said, it is worth noting that the correlations between the raw unconditional and conditional importance values were exceptionally high in 2013 (*r* = 0.99, *P* < 0.001) and 2021 (*r* = 0.87, *P* < 0.001).

#### Partial dependence + individual conditional expectation curves

To examine how time spent on activities related to the probability of having a good day, we used partial dependence (PD (26)) and individual conditional expectation (ICE (27)) curves (18). We created the PD + ICE curves for the variables of interest by fitting the best model from the model fitting procedure on the entire dataset (i.e. the training + test sets); we used the entire dataset because this allowed us to examine the model prediction across the complete range of observed predictor values, providing maximum information with no impact on model performance estimates. For each participant, the model recalculated their estimated probability of having a “better” than typical day if they had spent between 0 and 960 minutes (16 hours) on each activity of interest. Essentially, for any given participant we asked, “What would their predicted probability of having a good day have been if they had spent [any value between 0 and 960] minutes with family instead of their reported number of minutes?,” and we repeated this procedure for every activity. Specifically, we calculated a new estimated probability of having a good day for each 15-minutes increment between 0 and 16 hours, meaning that each participant had 65 estimated probabilities of having a good day, each corresponding to a specific amount of time devoted to an activity of interest. In the plots, we only included a random subset of ICE curves to avoid cluttering the graphs. However, the PD curves included in the plots are the average of the ICE curves created for all the participants and reflect the overall pattern in how predicted probabilities vary across the time spent on each activity. We “centered” the curves for plotting, such that we subtracted the estimated probability of having a good day at 0 minutes from all other values, so that each curve starts at zero ((18); see the Supplementary material for noncentered versions of the same plots).

## Results

We present the results from both the 2013 and 2021 analyses with a focus on the insights that were consistent across both samples.

### How accurately could the models predict whether someone had a better than typical day?

Overall, our models were able to accurately predict 65% (2021) and 64% (2013) of cases in the test samples for each year (Table 1). “Balanced accuracy,” however, offers a more meaningful measure of model performance when dealing with imbalanced data, as it averages the model's ability to correctly identify both good days and typical days, giving equal importance to each. Table 2 provides several fit statistics for our 2021 and 2013 analyses, including accuracy, balanced accuracy and area under the curve (AUC).

**Table 1.** Confusion matrices of model predictions in 2021 and 2013 test datasets.

	2021 (*n*_test set_ = 1,239)		2013 (*n*_test set_ = 1,857)	
Model prediction	Actual response		Actual response	
	Better than typical	Same as typical	Better than typical	Same as typical
Better than typical	**216**	289	**322**	434
Same as typical	149	**585**	241	**860**

Bolded cells indicate the number of correct model predictions.

**Table 2.** Model fit statistics for 2021 and 2013 analyses.

	2021	2013
Overall accuracy	0.65 (95% CI 0.61–0.67)	0.64 (95% CI 0.61–0.66)
Balanced accuracy	0.63	0.62
AUC	0.67	0.66
Cohen’s *κ*	0.23	0.22
Sensitivity (proportion of correctly identified better days)	0.59	0.57
Specificity (proportion of correctly identified typical days)	0.67	0.66

Fit statistics reflect the model performance on the test set.

To evaluate the fit of our models, we compared the balanced accuracy of our preregistered RF models to three alternative linear models: a LASSO model, a logistic regression model, and a baseline model that always predicted the majority case in the entire dataset. According to balanced accuracy, our RF models (2021: 63.06%; 2013: 61.83%) showed a slight advantage compared with the LASSO and logistic regression models, though the difference was typically <1%. Importantly, our RF models also showed a substantial improvement in accuracy compared with the baseline models (i.e. >10% increase in balanced accuracy).

Although the difference in balanced accuracy between the RF and the LASSO and logistic regression models was relatively small, the RF models additionally showed a greater ability to reliably identify good days (i.e. ∼4–5% improvement in sensitivity; see Supplementary material for more details on model comparisons). Given our focus was on good days, this increased sensitivity for identifying good days supports our use of nonlinear RF models which were able to capture meaningful patterns in the data (such as initial increases followed by a plateau). It is also worth noting that there was large overlap in the most important variables identified by our preregistered random forest models and the LASSO regression, supporting the robustness of our findings (see Supplementary material).

### What activities were most associated with having a better than typical day?

To identify which activities were most related to having a better than typical day, we calculated unconditional and conditional permutation importance values. These values indicate the importance of each activity for increasing the model's predictive accuracy but do not indicate the direction of the association. An activity could be important because it is strongly negatively or positively associated with having a good day, or because it is involved in complex interaction effects. The 20 most important variables as ranked by unconditional importance in our 2021 dataset are available in Table 3. The correlation between the raw unconditional importance values in 2013 and 2021 was extremely high, *r* = 0.88, *P* < 0.001.

**Table 3.** Activities ranked by 2021 unconditional importance value, with 2013 unconditional importance.

Activity	Unconditional importance ranking		Average time spent (m) 2021		Average time spent (m) 2013	
	2021	2013	Typical days	Good days	Typical days	Good days
Time with family^a^	1	1	263.30	366.98	286.82	368.98
Socializing and communicating	2	5	28.41	58.59	35.40	60.68
Travel related to eating and drinking	3	2	4.33	8.49	5.73	11.08
Working	4	7	166.09	109.63	160.58	115.32
Travel related to socializing, relaxing, and leisure	5	3	6.16	14.65	9.22	17.76
Time with friends^a^	6	6	21.43	49.76	48.91	94.71
Time alone with spouse^a^	7	23	110.20	104.99	87.70	83.92
Time with own household children^a^	8	13	85.68	131.21	116.24	147.48
Eating and drinking	9	10	67.33	75.60	66.01	73.57
Relaxing and leisure	10	4	280.19	238.55	266.36	211.62
Housework	11	25	42.39	34.21	41.41	36.68
Travel related to sports, exercise, and recreation	12	19	2.05	3.94	2.06	4.35
Providing secondary childcare for household children^a^	13	18	84.42	124.34	109.21	140.72
Time with spouse (alone and with others)^a^	14	8	166.68	205.51	153.49	189.46
Travel related to work (i.e. commuting)	15	9	10.36	7.09	13.02	8.91
Tending to lawn, garden, and houseplants	16	43	20.10	13.41	13.75	11.40
Participating in sports, exercise, and recreation	17	17	18.01	23.34	15.50	25.76
Travel related to consumer purchases	18	14	12.95	16.89	13.54	19.40
Travel related to caring for and helping nonhousehold members	19	20	2.62	4.66	2.84	5.08
Caring for and helping nonhousehold children	20	28	3.69	8.41	4.35	5.82

The importance rankings do not indicate whether engaging in a behavior increases or decreases the likelihood of having a good day; they simply indicate that a variable is important for increasing the accuracy of model prediction. We have provided the mean number of minutes spent on each activity across good and typical days for both years to provide readers with a sense of the direction of the effect of each activity.

^a^Variable created by combining all activities where participants indicated they were with the person of interest (e.g. a family member). In contrast to the all the other activity categories, these variables are not mutually exclusive (i.e. one single activity could count towards time with friends and time with family).

It is worth noting that we conducted exploratory analyses that separately analyzed the participants who described how they spent a weekend day (i.e. a Saturday or Sunday) and those who described how they spent a weekday (i.e. Monday—Friday). The correlation between the raw unconditional importance values for activities on weekends and weekdays was quite high in both 2013 (*r* = 0.75, *P* < 0.001) and 2021 (*r* = 0.68, *P* < 0.001), suggesting that there were not large distinctions between the activities that best described better than typical weekends vs. better than typical weekdays (see Supplementary material for additional details on these analyses).

### How does the amount of time spent on each activity relate to the probability of having a good day?

**Fig. 1.**
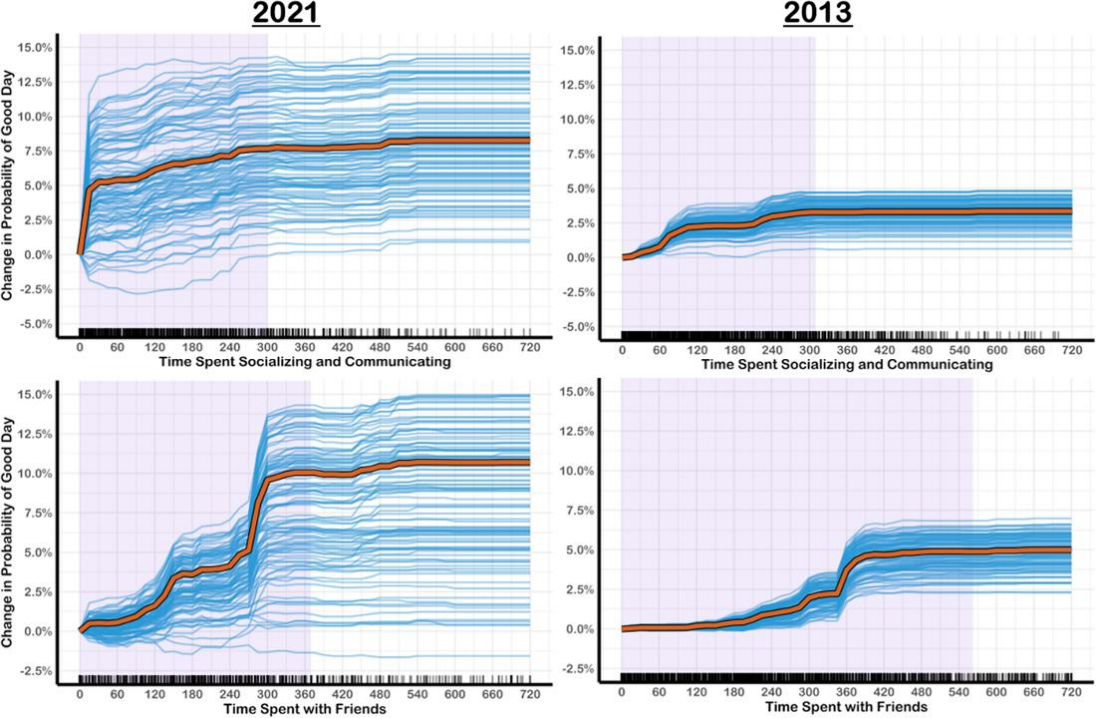
ICE and PD curves for time spent on socializing and time spent with friends. The thin blue lines are ICE curves for a random subset of participants. The thick orange lines are the PD curves based on the full sample of participants. The purple-shaded area of plot represents 2.5th and 97.5th percentiles of the predictor variable.

**Fig. 2.**
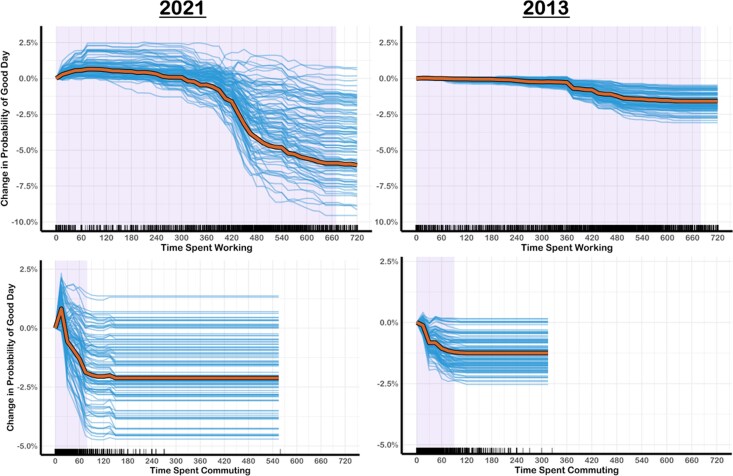
ICE and PD curves for time spent working and commuting. The thin blue lines are ICE curves for a random subset of participants. The thick orange lines are the PD curves based on the full sample of participants. The purple-shaded area of plot represents 2.5th and 97.5th percentiles of the predictor variable.

**Fig. 3.**
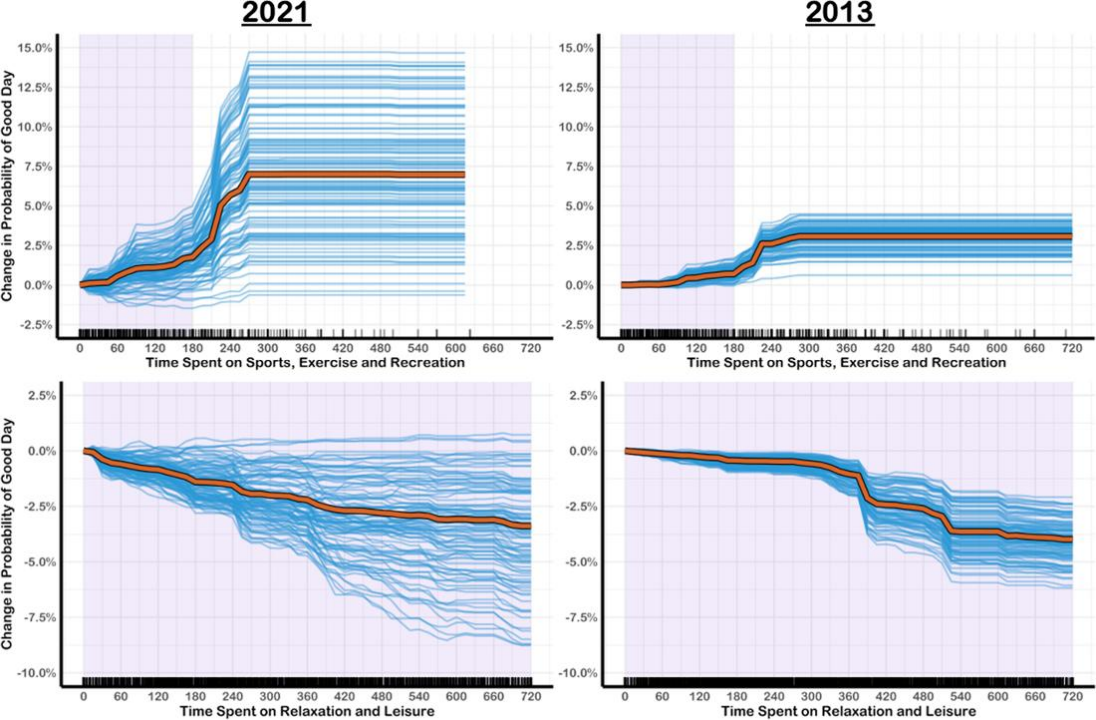
ICE and PD curves for time spent on sports and exercise and relaxing and leisure. The thin blue lines are ICE curves for a random subset of participants. The thick orange lines are the PD curves based on the full sample of participants. The purple-shaded area of plot represents 2.5th and 97.5th percentiles of the predictor variable. (97.5th percentile for work is beyond the bounds of the plot).

As mentioned, we used PD (26) and ICE (27) curves to examine how time spent on activities related to the probability of having a better than typical day (18). ICE curves reveal how varying the time spent on an activity is associated with changes in an individual's predicted probability of reporting a good day. For example, if Alex reported spending sixty minutes with friends, we can see how her predicted probability of having a good day would change if she had instead spent 15 min, 30 min, or several hours with friends. PD curves are averages of these individual ICE curves across all participants, providing an overall trend of how time spent on an activity relates to the predicted probability of having a good day in the entire sample. Below, we highlight the PD + ICE plots for the variables in Table 3 that showed consistent relationships across both our samples (see Supplementary material for PD + ICE plots for all variables presented in Table 3). In general, the associations between time on any activity and the probability of having a good day tended to be larger in 2021 than in 2013 (Figs. 1–3).

#### Social behaviors

Time spent socializing, time with family, and time with friends were in the top six most important predictors across both 2013 and 2021 (Table 3). For an activity to be categorized as “socializing and communicating” in the ATUS, the primary purpose of the activity had to be socializing. Values for “time with family” and “time with friends,” in contrast, are calculated by combining all activities where participants indicated a family member or friend was present. Across both the 2021 and 2013 analyses, PD + ICE plots revealed that there was little marginal increase in the probability of having a good day after socializing for more than 2 hours, and in 2021, the predicted probabilities plateaued at less than 1 hour of socializing (Fig. 1). In contrast, the probability of reporting a good day increased steadily with time spent with friends, continuing to rise for up to 5–6 hours (Fig. 1).

#### Work and commuting

Time spent working ranked as one of the most important variables in predicting whether people had a good day, ranking seventh in 2013 and fourth in 2021 (Table 3). The PD + ICE curves show that working was unrelated to having a good day for up to 6 hours, at which point it quickly became negatively associated with having a good day (Fig. 2). Commuting was also within the top 20 most important variables in both 2021 and 2013, according to the unconditional importance values. Notably, in 2021 brief (15-min), commutes showed a slight positive association with having a good day, possibly because people enjoyed days when they had a reason to leave their house during the COVID-19 pandemic. Other than this small positive relationship in 2021, commuting for up to 90 min was increasingly negatively associated with having a good day. It is worth noting that very few people reported commutes of >90 min in 2013 (2.38%) or 2021 (1.63%).

#### Active and passive leisure

Time spent on sports and exercise and time spent on relaxing and leisure were rated as important variables in both samples (Table 3). Time spent on sports and exercise was positively associated with having a good day up until ∼5 hours, after which predicted probabilities began to level off (Fig. 3). Surprisingly, spending any amount of time on relaxation and leisure was associated with a lower probability of having a good day, with the strongest negative association appearing in 2021. In interpreting this finding, it is worth noting that “watching television and movies” accounted for most of the minutes people spent on relaxation and leisure in both 2021 (70%) and 2013 (72%).

### What are some examples of a good day?

As an exploratory analysis to further illustrate the pattern of activities that characterize a good day, we identified participants in each year's holdout sample who were assigned the highest probability of having a good day by our model. In 2021, the highest probability was 0.82, while in 2013, it was 0.66. As shown in Table 4, both participants spent over 13 hours with family and spent ∼4 hours on sports and exercise. The 2021 participant also reported 2 hours dedicated to socializing. The 2013 participant, in contrast, spent significant amounts of time with friends (590 minutes), their spouse (860 minutes), and their own children (795 minutes).

**Table 4.** Minutes spent on 20 most important activities by the participant from 2013 and 2021 assigned the highest probability of having a better than typical day.

Activity	Minutes devoted to each activity	
	2021 participant with the highest probability of having a good day	2013 participant with the highest probability of having a good day
Time with family^a^	821	875
Socializing and communicating	120	0
Travel related to eating and drinking	50	5
Working	0	0
Travel related to socializing, relaxing, and leisure	0	10
Time with friends^a^	0	590
Time alone with spouse^a^	0	80
Time with own household children^a^	0	795
Eating and Drinking	360	48
Relaxing and leisure	0	90
Housework	0	0
Travel related to sports, exercise, and recreation	45	55
Providing secondary childcare for household children^a^	0	745
Time with spouse (alone and with others)^a^	0	860
Travel related to work (i.e. commuting)	0	0
Tending to lawn, garden, and houseplants	0	0
Participating in sports, exercise, and recreation	246	250
Travel related to consumer purchases	0	158
Travel related to caring for and helping nonhousehold members	0	95
Caring for and helping nonhousehold children	0	12

^a^Variable created by combining all activities where participants indicated they were with the person of interest (e.g. a family member). In contrast to all the other activity categories, these variables are not mutually exclusive (i.e. one single activity could count towards time with friends and time with family).

## Discussion

Life requires complex trade-offs between competing activities, but the present research reveals clear patterns that differentiate better days from typical ones. Indeed, our random forest models achieved up to 63% balanced accuracy, substantially outperforming the 50% balanced accuracy expected by chance alone. According to these models, socializing for 30 minutes (vs. 0 minutes) was associated with a higher likelihood of reporting a good day. Beyond 2 hours, however, there was little marginal increase in the probability of having a good day. In contrast, the probability of reporting a good day increased steadily with time spent with friends, continuing to rise for up to 5–6 hours. While past research shows that people tend to experience low levels of happiness when they are working (1, 8), we found that working for up to 6 hours was unrelated to whether people rated their day as better than usual. When individuals worked for more than 6 hours though, their predicted probability of having a good day rapidly declined. Another surprising pattern was that time spent relaxing was always negatively associated with having a better than typical day.

It is important to underscore that our findings are descriptive in nature and cannot be used to infer causality. While the findings presented here help identify the common ingredients of a good day, some activities may be more commonly observed on better days not because the activities are inherently enjoyable, but because they accompany other positive experiences. For example, “travel related to eating and drinking” was positively associated with having a good day, perhaps because a 30-minute drive for food could indicate a meal of perfectly seared scallops at a restaurant rather than freezer-burned fish sticks at home. Additionally, we cannot rule out bidirectional effects—people may have been more likely to engage in some activities because they were already feeling particularly good or bad. Indeed, bidirectional effects likely help explain the negative association between relaxing and having a good day, because people probably spend more time relaxing when they are feeling down. Still, it is striking that even a little bit of relaxing (e.g. less than an hour) was negatively related to having a good day.

Although we have used the terms “better” day and “good” day interchangeably, it is important to emphasize that reporting a better than typical day is not necessarily equivalent to having what would universally be considered a good day. Because our outcome measure asked participants to rate their day relative to their own typical day, it is conceivable some individuals' “better” days were still relatively negative. Nevertheless, only 30% of the days in our sample were rated as better than typical, suggesting that participants applied a relatively stringent standard when identifying a better day.

It is also worth noting that additional confounders such as income, household composition, and personality could influence the patterns uncovered here. Indeed, it is possible that activities like socializing were associated with good days partially because extraverted, healthy individuals may both be more likely to socialize and more likely to report their days as better than typical. In addition, differences in income or education may fundamentally change the nature of some activities. An hour of working, for example, could mean the drudgery of data entry for a low-paid employee or an invigorating brainstorming session for a high-powered executive. Relatedly, time with one's children may be an entirely different experience for an overworked single mother vs. a married couple with an in-home nanny. Even without these crucial distinctions, however, our models still predicted good vs. typical days with up to 63% balanced accuracy.

We also found largely consistent results in 2013 and 2021, which is remarkable given the dramatic cultural shifts that occurred between these 2 years—including the release of TikTok, the first election of Donald Trump, the #MeToo movement, and the onset of the COVID-19 pandemic. Yet, the activities that characterized good days remained fundamentally the same. Indeed, the unconditional importance values for activities across 2013 and 2021 were extremely highly correlated (*r* = 0.88).

Taken together, our results highlight a pattern of activities that differentiate better days from typical ones. Given that resources for research are often limited, these findings offer a useful starting point for identifying promising avenues for future study. For example, we hope the patterns uncovered here help generate future causal work, such as research examining the emotional consequences of too much relaxation or whether the difference between 6- and 8-hour workdays is as substantial as our findings suggest.

More broadly, we hope this work encourages greater attention in happiness research to the trade-offs inherent in daily life, where time devoted to one activity necessarily comes at the expense of another. While the present results are descriptive in nature, they provide insight into the rhythms and routines that characterize good days.

## Supplementary Material

pgag014_Supplementary_Data

## Data Availability

Full details on the methodology of the American Time Use Survey are available at https://www.bls.gov/tus/. All primary data are available at https://tinyurl.com/tmek7c2d. The analysis scripts are available at https://osf.io/cf9n3/files?view_only=8c203fcebcf840f9aa2d63864955e79b.

## Preprints

This manuscript was originally posted on a preprint server at https://osf.io/preprints/psyarxiv/zs9t2_v5.

## Artificial Intelligence

The authors used AI (ChatGPT 4.omini and ChatGPT 5) for minor editing of the manuscript (e.g. typos, avoiding causal language, etc.), but all aspects of the manuscript were written by the authors.
