# Supplementary material for: What does a good day look like?: An interpretable machine learning approach to the American Time Use Survey
Source: PNAS Nexus. 2026 Mar 13;5(3):pgag014. doi: 10.1093/pnasnexus/pgag014 (PMC12983456; doi:10.1093/pnasnexus/pgag014)
Supplement: pgag014_Supplementary_Data [file pgag014_supplementary_data.pdf]

Table of Contents for Supplemental Online Material

Raw Unconditional and Conditional Importance Values for 2021 and 2013 .... *3*

Figure S1. Top 20 variables by unconditional and conditional importance in 2021 4

Figure S2. Top 20 variables by unconditional and conditional importance in 2013 5

Additional Model Accuracy Details *6*

Table S1. Fit statistics for pre-registered RF model and additional linear comparison models 6

Figure S3. Top 20 most important variables according to standardized LASSO coefficients in 2021 and 2013. 7

Uncentered ICE+PD Curves for Activities Featured in Main Text *8*

Figure S4. Uncentered ICE and PD curves for time spent on socializing and time spent with friends.... 9

Figure S5. Uncentered ICE and PD curves for time spent working and time spent commuting........... 10

Figure S6. Uncentered ICE and PD curves for time spent on sports, exercise, and recreation and time spent relaxing and leisure 11

Uncentered ICE+PD Curves for 14 Other Activities Not Featured in Main Text *12*

Figure S7. Uncentered ICE and PD curves for time spent with family and time spent on travel related to eating and drinking. 13

Figure S8. Uncentered ICE and PD curves for time spent on travel related to socializing, relaxing and leisure and time alone with spouse. 14

Figure S9. Uncentered ICE and PD curves for time spent with own children and time spent eating and drinking 15

Figure S10. Uncentered ICE and PD curves for time spent on housework and time spent on travel related to sports and exercise. 16

Figure S11. Uncentered ICE and PD curves for time spent providing secondary childcare to household children and time spent with spouse (alone or with others). 17

Figure S12. Uncentered ICE and PD curves for time spent tending to lawn, garden, and houseplants and time spent on travel related to consumer purchases. 18

Figure S13. Uncentered ICE and PD curves for time spent on travel related to caring for and helping non-household members and time spent caring for non-household children. 19

Examining Weekends and Weekdays Separately *20*

Table S2. Model fit information for 2021 weekday-only and weekend-only models. 20

Table S3. Model fit information for 2013 weekday-only and weekend-only models. 20

Figure S14. Top 20 variables by unconditional and conditional importance for 2021 weekday model. 21

Figure S15. Top 20 variables by unconditional and conditional importance for 2021 weekend model. 22

Figure S16. Top 20 variables by unconditional and conditional importance for 2013 weekday model. 23

Figure S17. Top 20 variables by unconditional and conditional importance for 2013 weekend model. 24

Appendix A: Data Dictionary for Time Use Portion of the ATUS (2013 and 2021) 25

S1: Introduction 27

S2: Household Roster 30

S3: Employment 34

S4: Time-use Diary 43

S5: Summary Questions 49

S7 (a): Labor Force Status: Layoff/Looking 66

S7 (b): Labor Force Status: Employed 72

S8: Earnings and School Enrollment 75

S9: Conclusion 79

Appendix B: Data Dictionary for Well-being Module of the 2013 ATUS 81

Appendix C: Data Dictionary for Well-being Module of the 2021 ATUS 92

Raw Unconditional and Conditional Importance Values for 2021 and 2013

Aside from the rankings provided in Table 1 in the main text, we also calculated the raw unconditional and conditional importance values across both 2021 and 2013. Figure S1 and S2 display the raw importance values for the top 20 most important variables (according to each importance metric) in 2021 and 2013. The orange line in each graph represents a cut-off point analogous to statistical significance. Specifically, we utilized a heuristic put forth by Strobl et al. (2009) which takes advantage of the fact that unimportant variables may sometimes receive importance values less than zero, simply due to sampling error. The orange line in each graph represents the absolute value of the largest negative unconditional/conditional importance value for each year. Variables that have a positive unconditional/conditional above this threshold can be viewed as “important”, because they have importance values higher that what would be expected simply from sampling error alone (i.e., chance).

Figure S1. Top 20 variables by unconditional and conditional importance in 2021

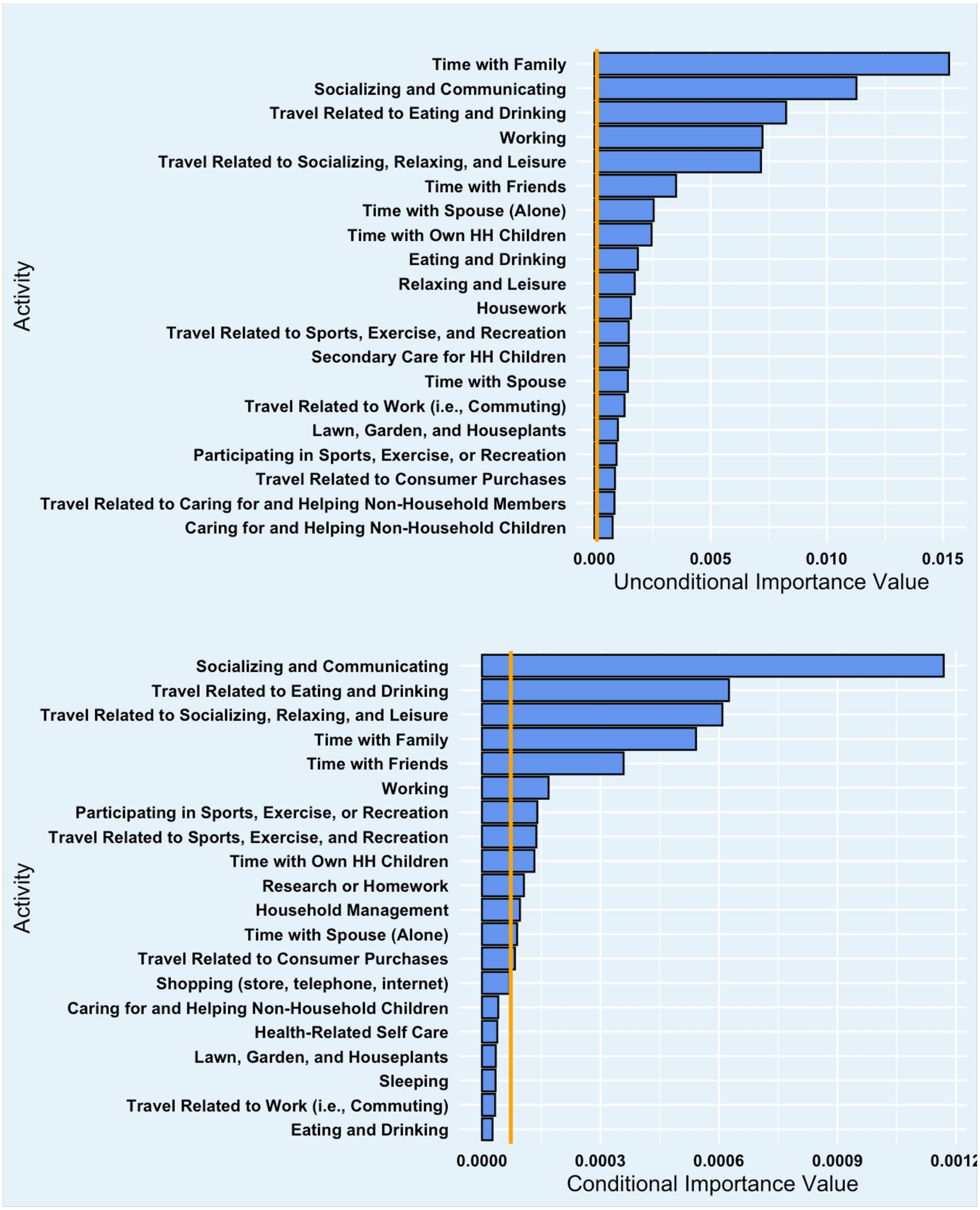

Figure S2. Top 20 variables by unconditional and conditional importance in 2013

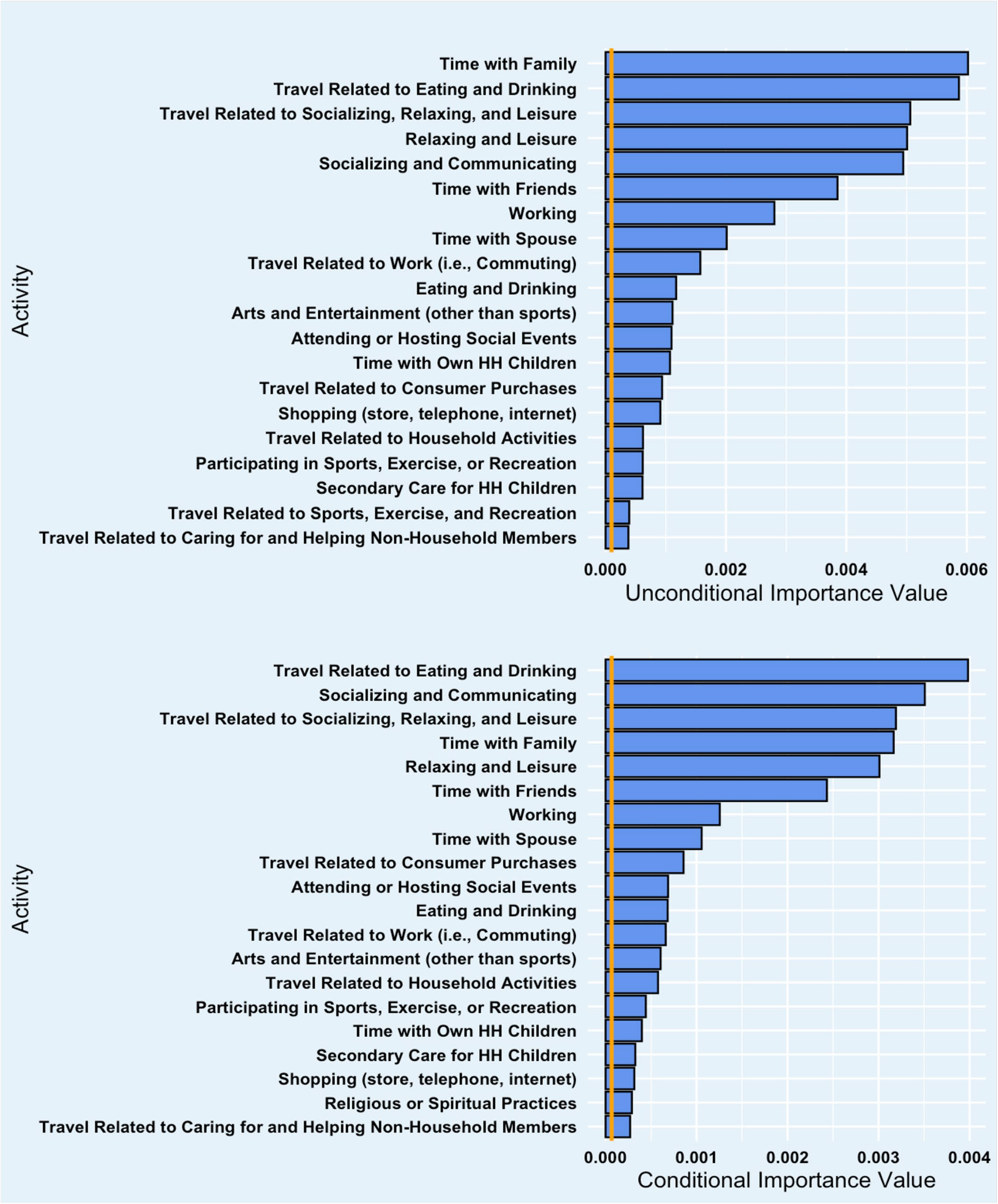

Additional Model Accuracy Details

As discussed in the main text, we compared our preregistered RF models to three linear models: a LASSO model, a logistic regression model, and a baseline model that always predicted the majority case. When fitting these additional models, we once again split the data into a training set (80%) and a test set (20%). The LASSO, logistic, and baseline models were fit to the training set using 5-fold cross-validation, and their accuracy was assessed on the test set. Because our pre-registered RF models were fit using OOB validation, we also fit an RF model using the same 5-fold cross-validation used to fit the linear models, for comparison. For the RF, LASSO, and logistic models, we used down sampling with the 5-fold cross-validation. We also standardized all the predictor variables before including them in the LASSO and logistic regression models. As shown in Table S1, our pre-registered RF model always outperformed the alternative models according to balanced accuracy. It also outperformed the other models at correctly identifying better-than-typical days (i.e., sensitivity). It is also worth noting that our RF model fit using 5-fold cross-validation also outperformed the other models according to balanced accuracy. These results support our interpretation of the non-linear effects uncovered by our pre-registered RF models.

Table S1. Fit statistics for pre-registered RF model and additional linear comparison models

Model	Accuracy (%)	Balanced Accuracy (%)	Sensitivity (proportion of correctly identified better days)	Specificity (proportion of correctly identified typical days)	AUC
2021					
Pre-registered RF model	64.65	63.06	.5918	.6693	.671
RF model fit with 5-factor CV	64.89	63.15	.5890	.6739	.676
LASSO model	65.05	62.23	.5534	.6911	.650
Logistic regression	63.28	60.65	.5425	.6705	.638
Baseline model	70.54	50.00	.0000	1.000	.500
2013					
Pre-registered RF model	63.65	61.83	.5719	.6646	.657
RF model fit with 5-factor CV	63.00	61.81	.5879	.6484	.668
LASSO model	64.84	61.57	.5329	.6986	.664
Logistic regression	64.30	61.14	.5311	.6917	.667
Baseline model	69.68	50.00	.0000	1.000	.500

To further explore the robustness of our primary analyses, we compared the importance values for each activity as estimated by our random forest models with the importance values for each activity as estimated by the LASSO regression. In contrast to random forest models,

importance values do not need to be calculated separately for the predictors in a LASSO regression. Instead, the standardized coefficients associated with each predictor in the LASSO model represent the predictive importance of each variable (analogous to coefficients in a standard regression model). As such, we compared the standardized LASSO coefficients for each activity in 2021 with the unconditional and conditional importance values for each activity in 2021, and the standardized LASSO coefficients for each activity in 2013 with the corresponding unconditional and conditional importance values.

Within both years, the standardized lasso coefficients for each activity were highly correlated with both the unconditional (2021: $r = .69$, $p <. 001$; 2013: $r = .76$, $p < .001$) and conditional importance (2021: $r = .49$, $p < .001$; 2013: $r = .74$, $p <.001$) values. Figure S3 provides the top 20 most important variables across the two years according to the standardized LASSO coefficients. There was substantial overlap among the most important variables ranked by the LASSO model and by our preregistered random forest. For example, time with family, time with friends, relaxing and leisure, socializing and communicating, and working were all among the top ten activities by standardized lasso coefficients and unconditional importance values in both years.

Figure S3. Top 20 most important variables according to standardized LASSO coefficients in 2021 and 2013.

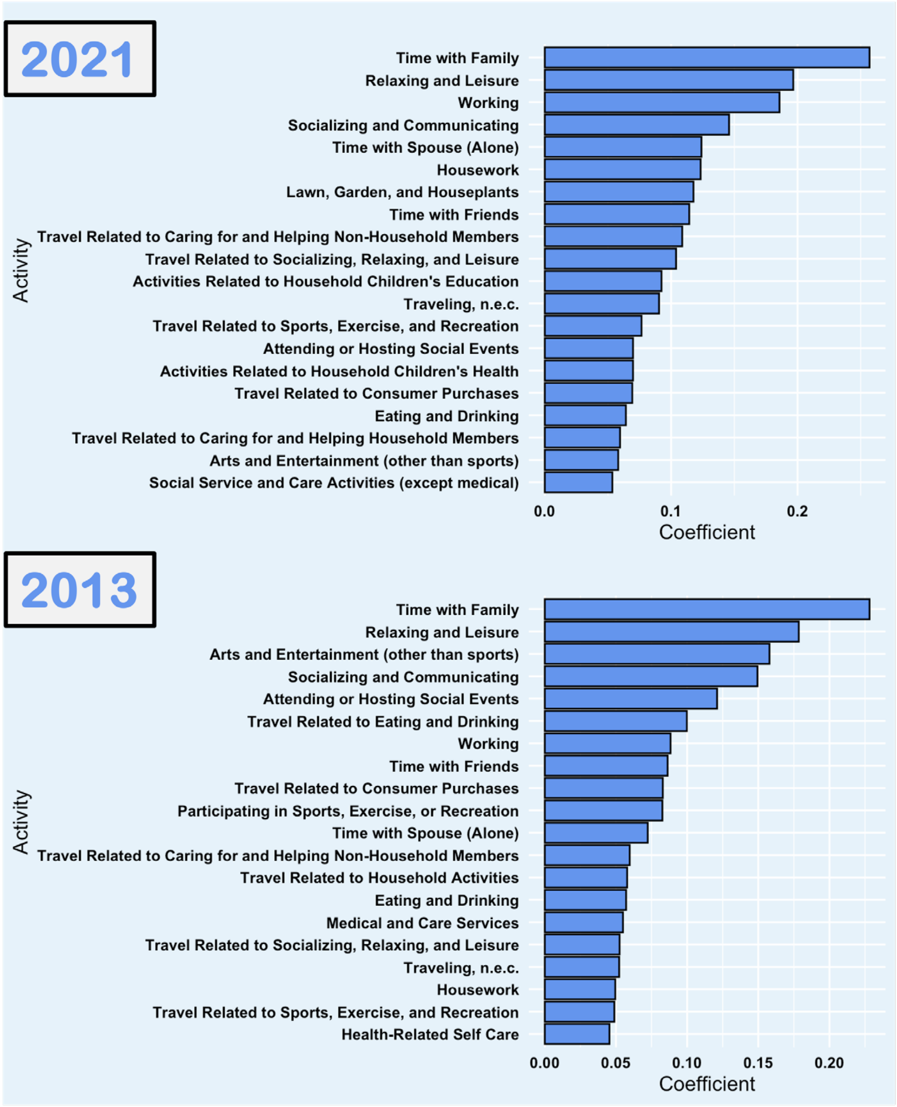

Uncentered ICE+PD Curves for Activities Featured in Main Text

As noted in the main text, we centered the ICE+ PD curves such that we subtracted the estimated probability of having a good day at 0 minutes from all other values, so that each curve starts at zero. Below, in Figures S3-S6, we provide the non-centered ICE+PD curves for the activities examined in the main text. For these plots, the model's raw probability estimates of a good day are presented on the y-axis, rather than the change in probability (which was the case in the centered graphs).

Figure S4. Uncentered ICE and PD curves for time spent on socializing and time spent with friends.

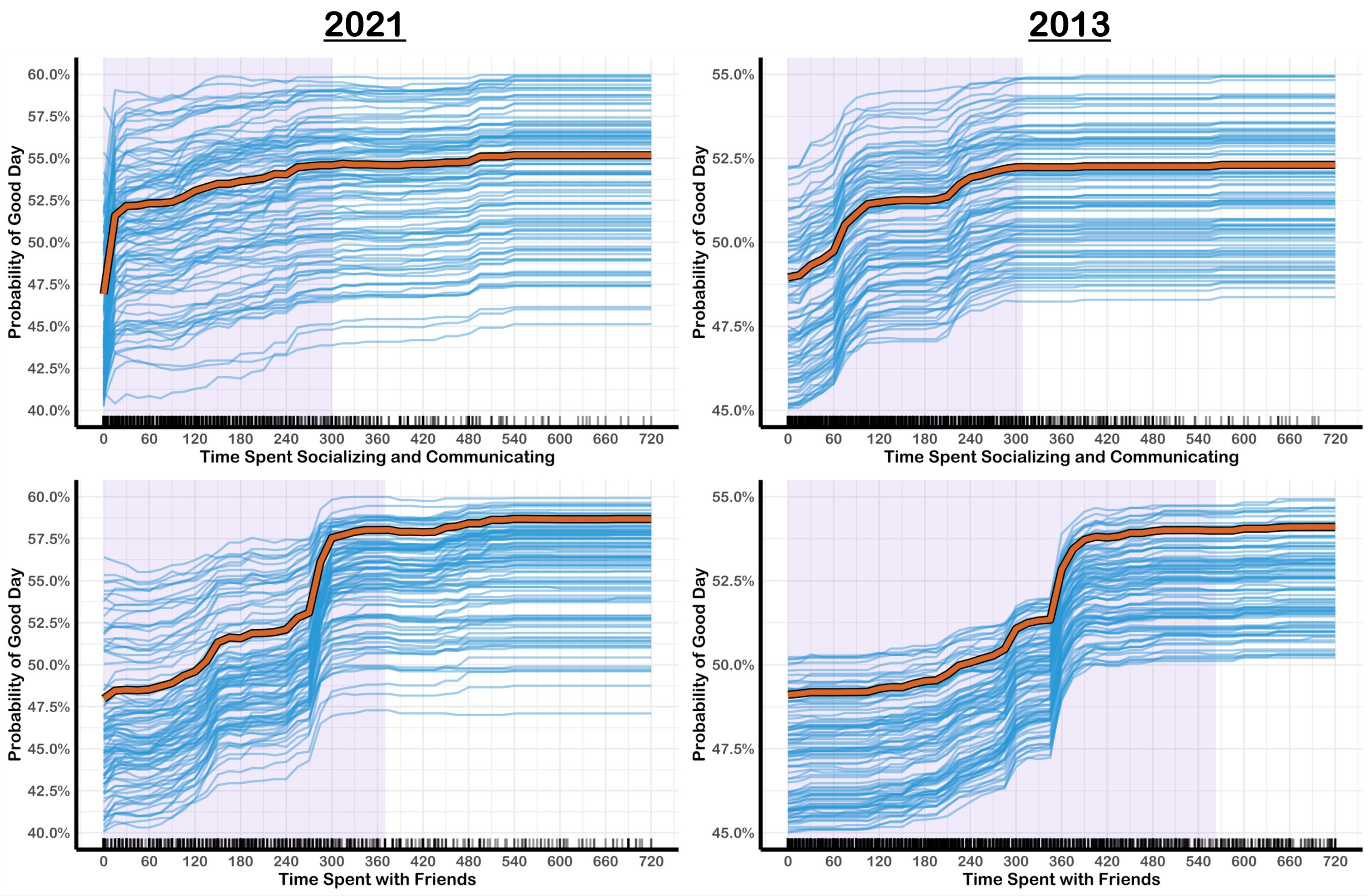

Figure S5. Uncentered ICE and PD curves for time spent working and time spent commuting.

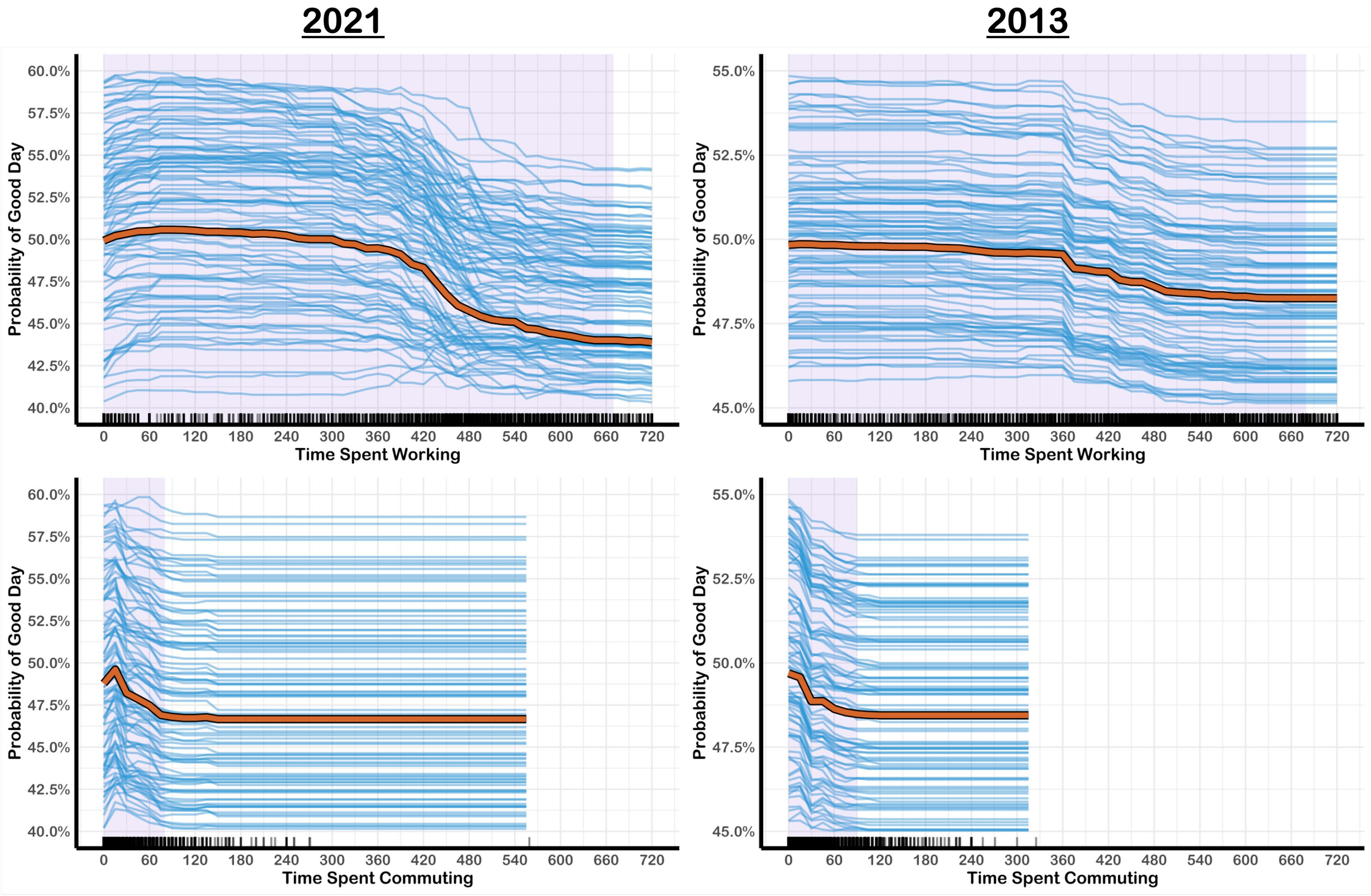

Figure S6. Uncentered ICE and PD curves for time spent on sports, exercise, and recreation and time spent relaxing and leisure

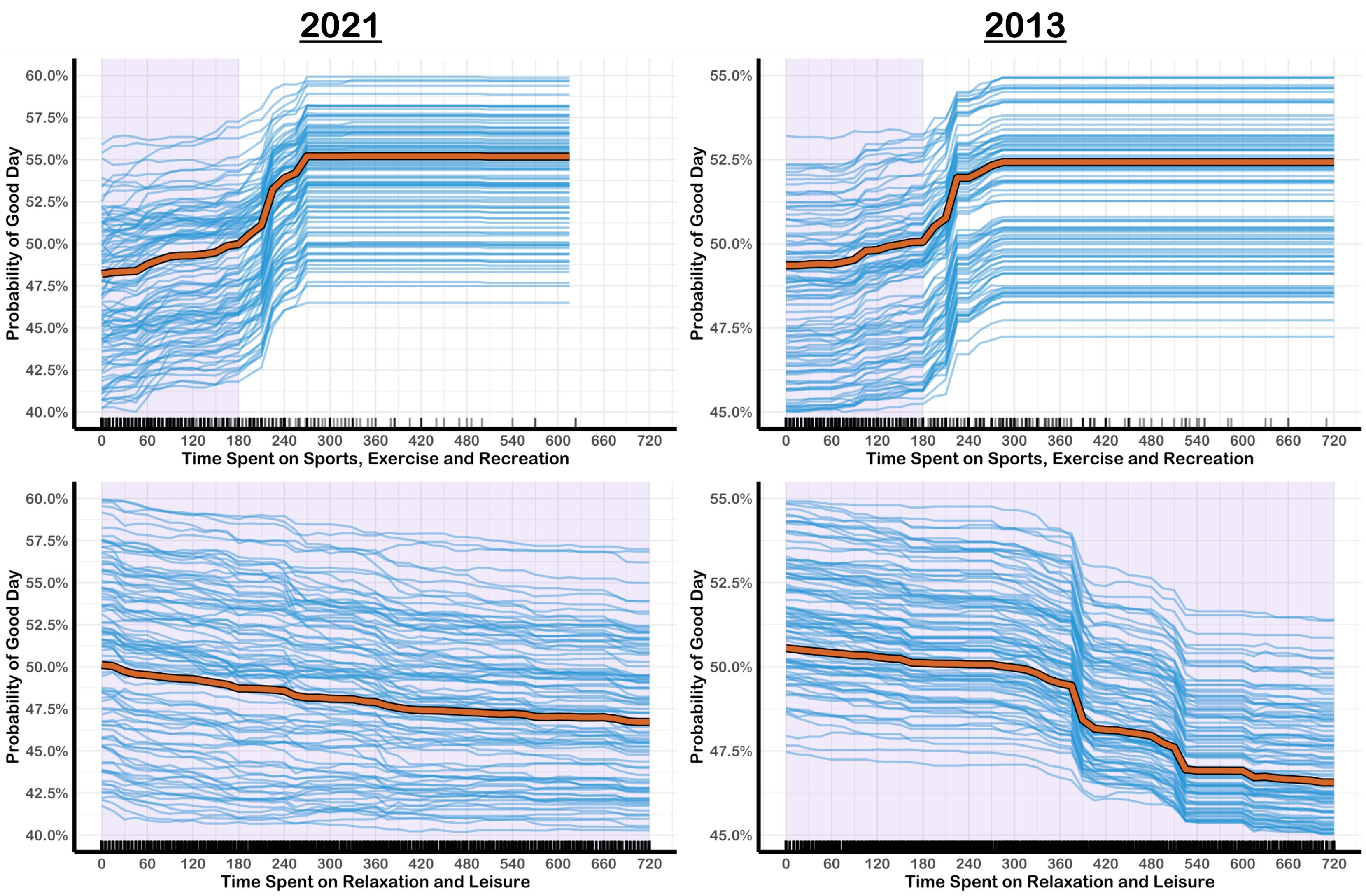

Uncentered ICE+PD Curves for 14 Other Activities Not Featured in Main Text

In the main text, we focused on 6 of the top 20 most important variables displayed in Table 1. Below, in Figures S7 – S13, we provide the uncentered ICE+PD curves for the remaining variables featured in Table 1.

Figure S7. Uncentered ICE and PD curves for time spent with family and time spent on travel related to eating and drinking.

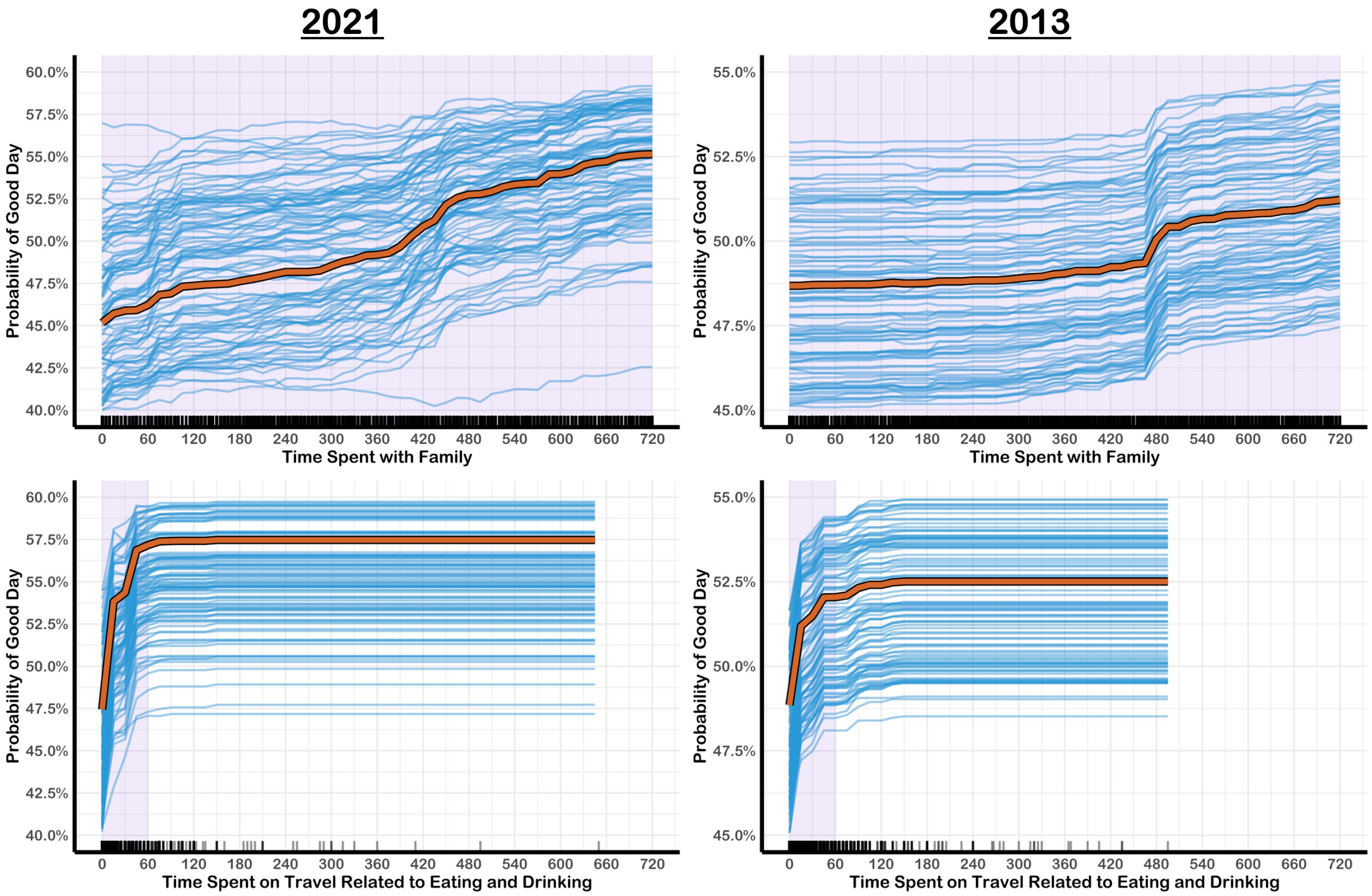

Figure S8. Uncentered ICE and PD curves for time spent on travel related to socializing, relaxing and leisure and time alone with spouse.

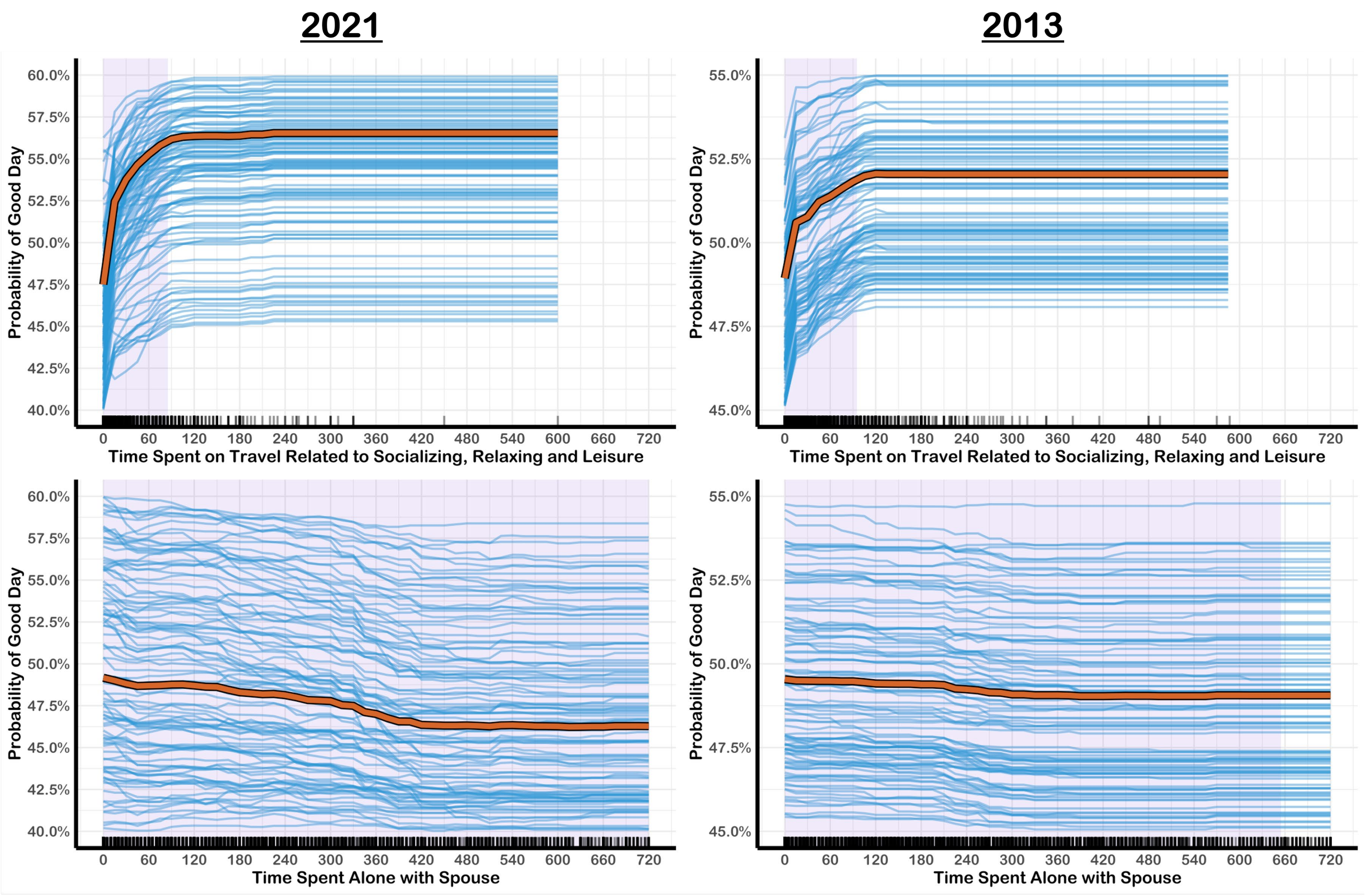

Figure S9. Uncentered ICE and PD curves for time spent with own children and time spent eating and drinking.

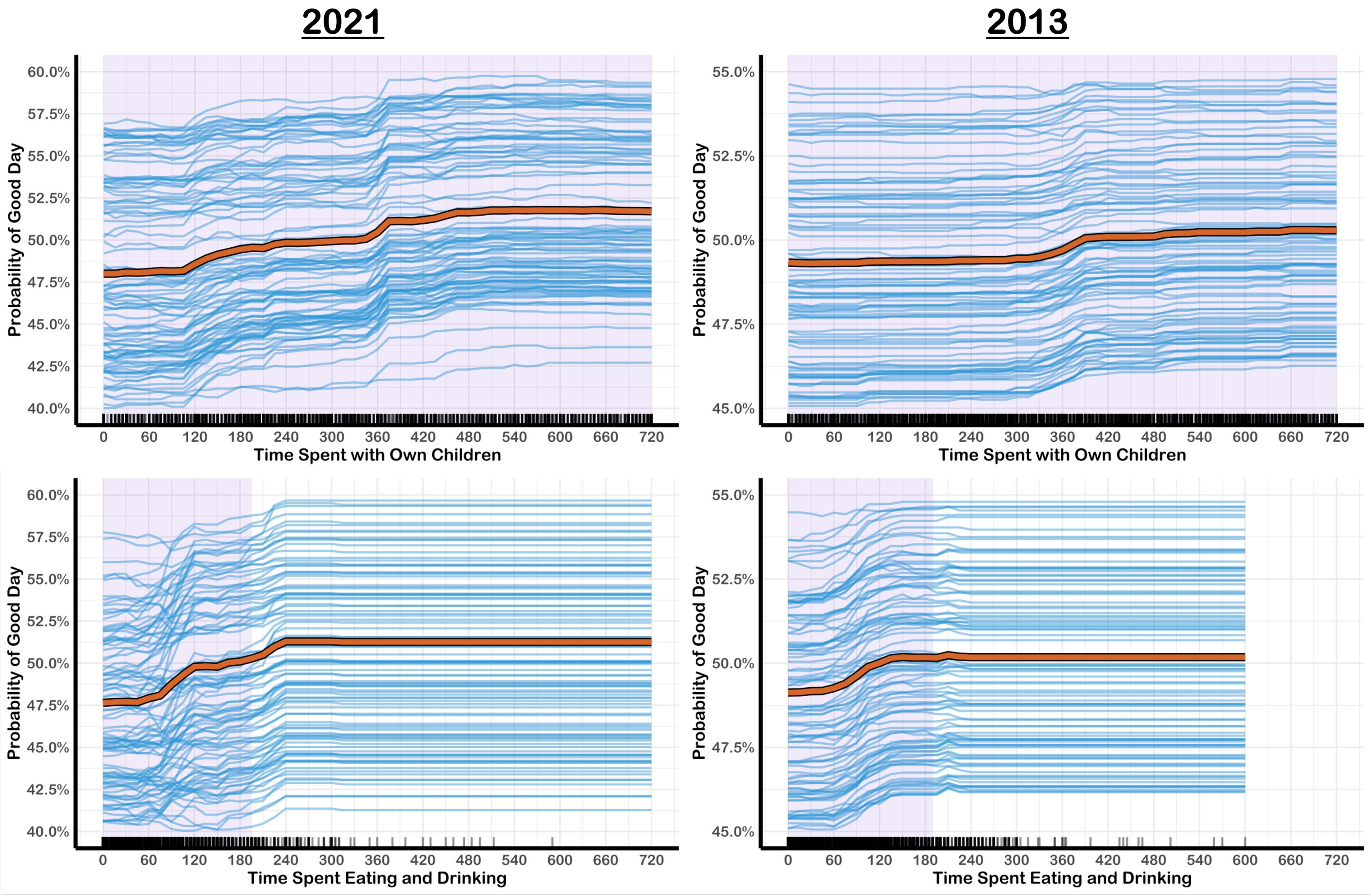

Figure S10. Uncentered ICE and PD curves for time spent on housework and time spent on travel related to sports and exercise.

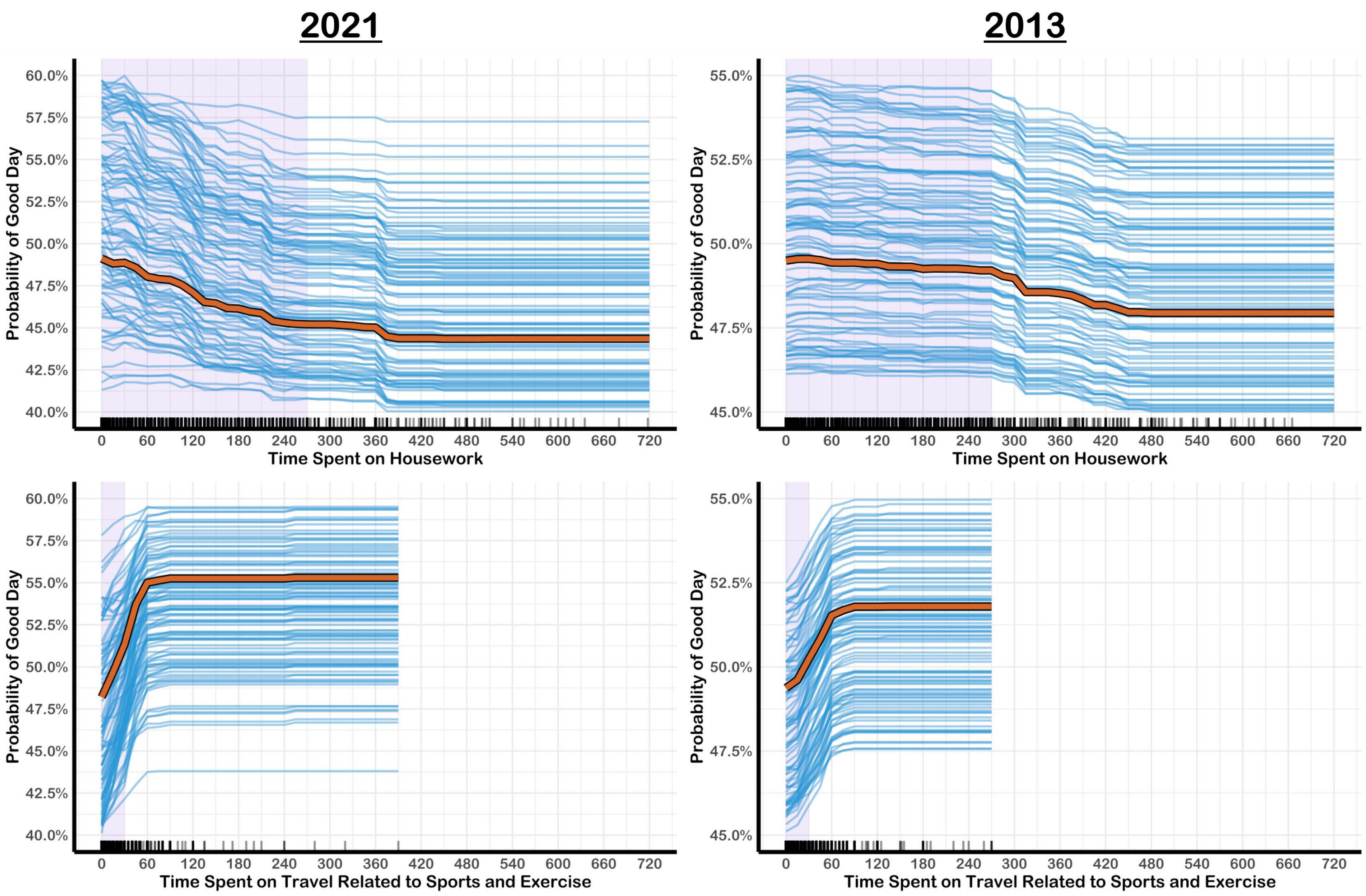

Figure S11. Uncentered ICE and PD curves for time spent providing secondary childcare to household children and time spent with spouse (alone or with others).

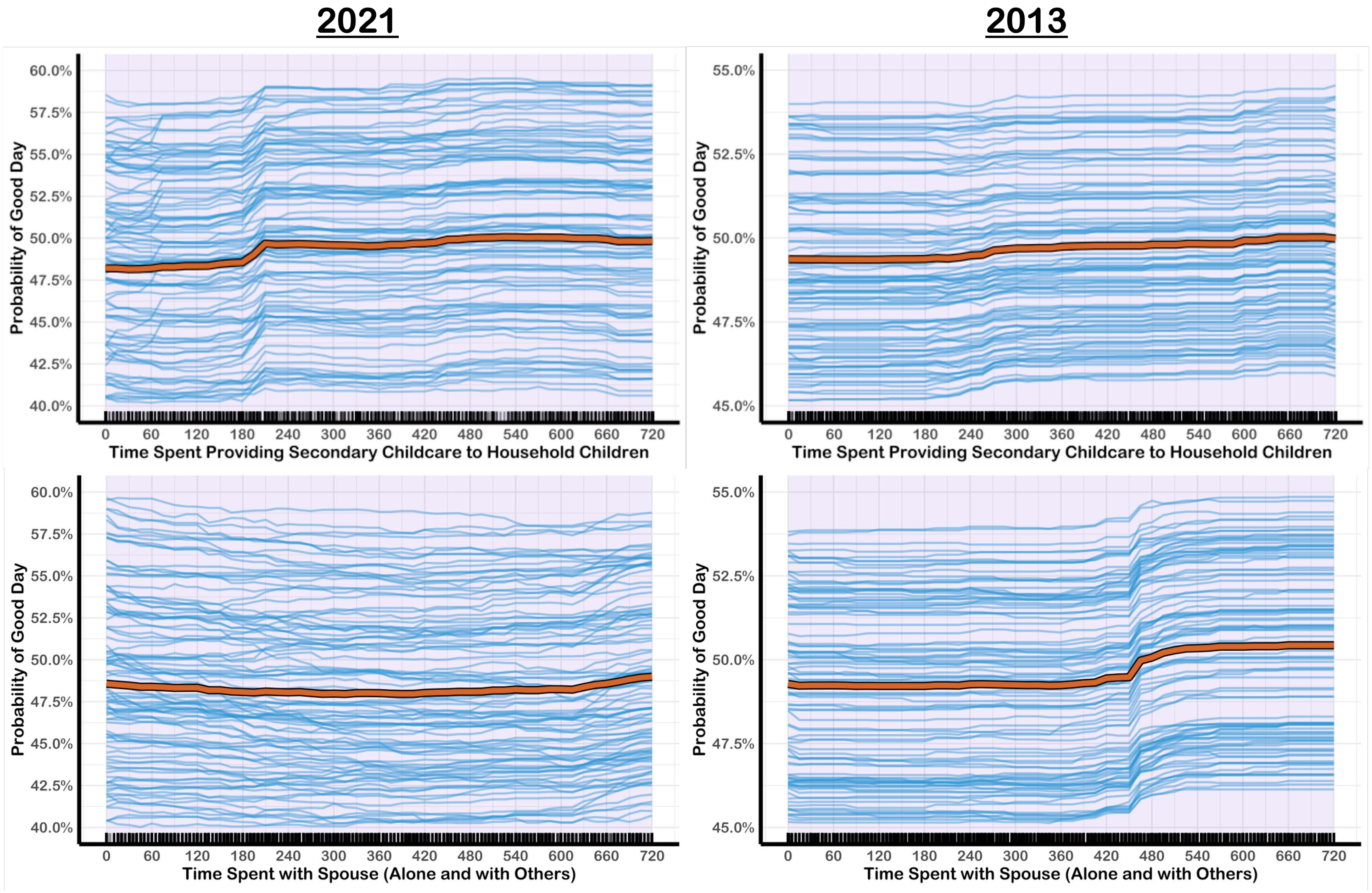

Figure S12. Uncentered ICE and PD curves for time spent tending to lawn, garden, and houseplants and time spent on travel related to consumer purchases.

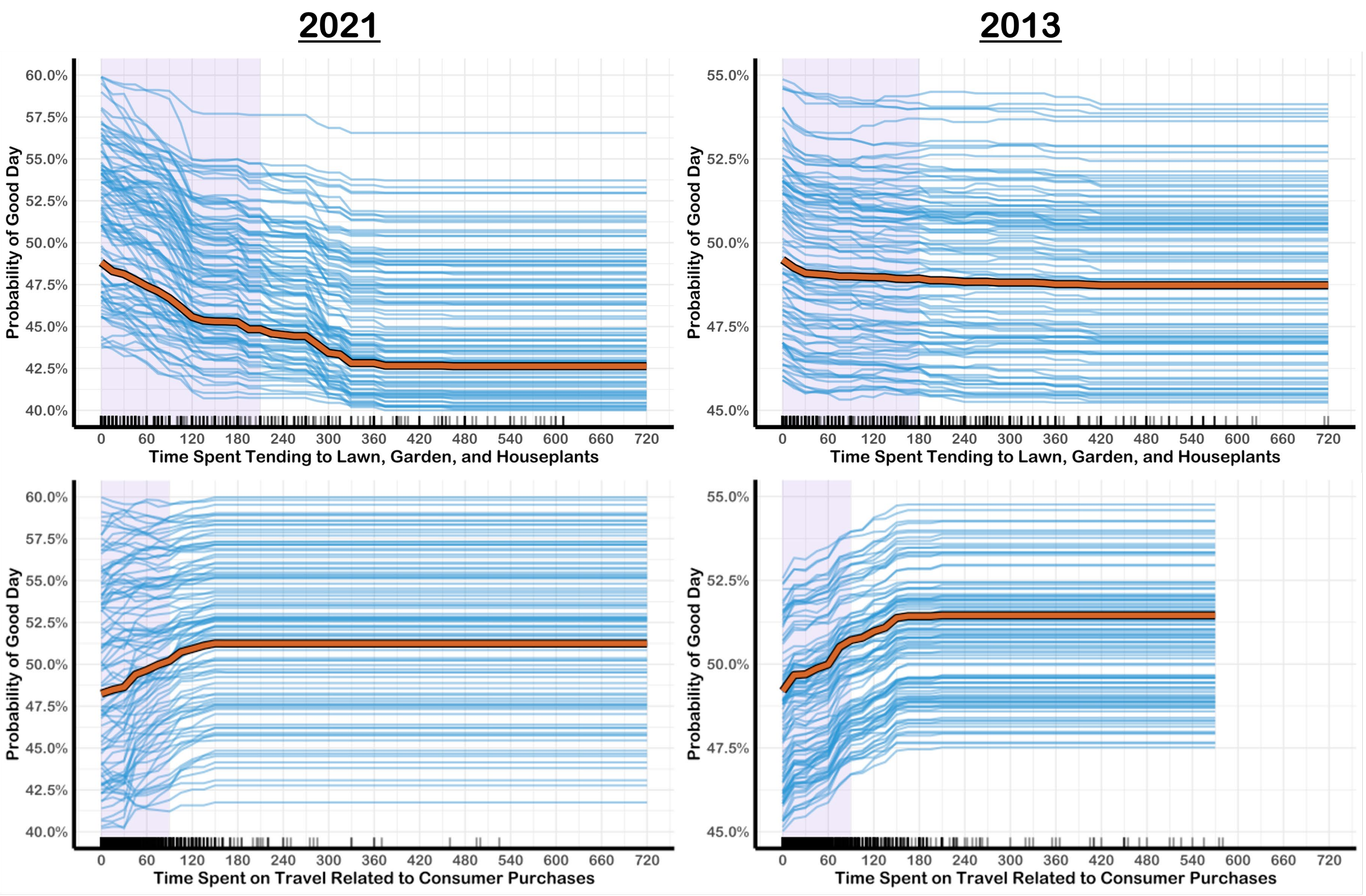

Figure S13. Uncentered ICE and PD curves for time spent on travel related to caring for and helping non-household members and time spent caring for non-household children.

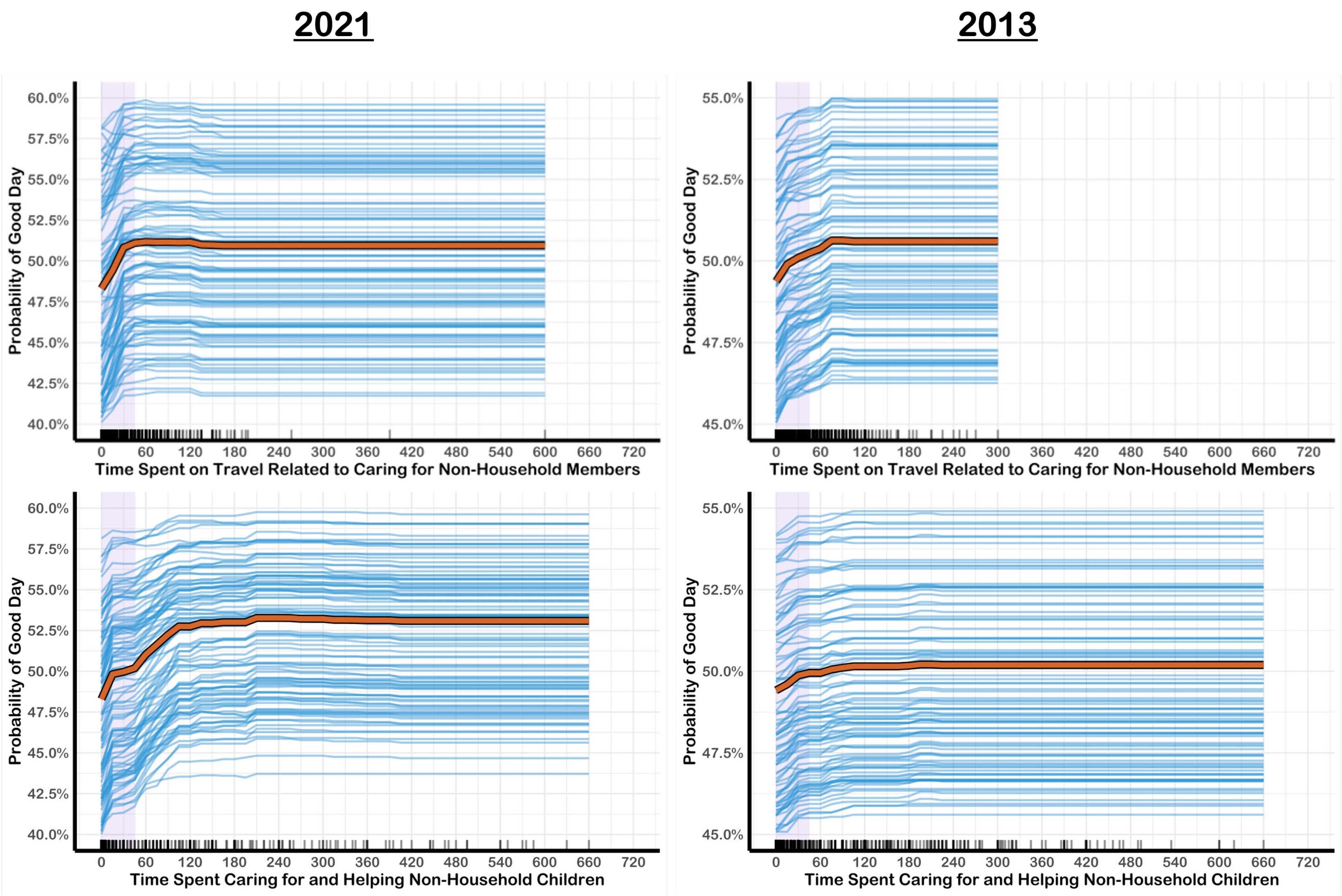

Examining Weekends and Weekdays Separately

As mentioned in the main text, we also fit random forests models to datasets consisting of only weekdays (i.e., Monday – Friday) and only weekends (i.e., Saturday-Sunday) for across 2013 and 2021. Our model fitting process was identical to our primary analyses. In 2021, the model fit to the weekend-only data was somewhat more accurate than the weekday model (see Table S2), but in 2013, it was the weekday model that was slightly more accurate than the weekend model (see Table S3). Overall, though, the accuracy for these models was generally similar to the accuracy of our primary models. Figures S14-S17 display the top 20 activities by unconditional and conditional importance for each of the models.

Table S2. Model fit information for 2021 weekday-only and weekend-only models.

	Weekdays	Weekends
Overall accuracy	.66<br>(95% CI: .62 - .70)	.72<br>(95% CI: .69 - .76)
Cohens κ	.26	.40
Balanced accuracy	.66	.71
Sensitivity	.65	.67
Specificity	.66	.75

Note: For sensitivity and specificity estimates, “good days” are treated as the positive case.

Table S3. Model fit information for 2013 weekday-only and weekend-only models.

	Weekdays	Weekends
Overall accuracy	.66<br>(95% CI: .62 - .69)	.60<br>(95% CI: .56 - .63)
Cohens κ	.22	.19
Balanced accuracy	.63	.60
Sensitivity	.56	.62
Specificity	.69	.58

Note: For sensitivity and specificity estimates, “good days” are treated as the positive case.

Figure S14. Top 20 variables by unconditional and conditional importance for 2021 weekday model.

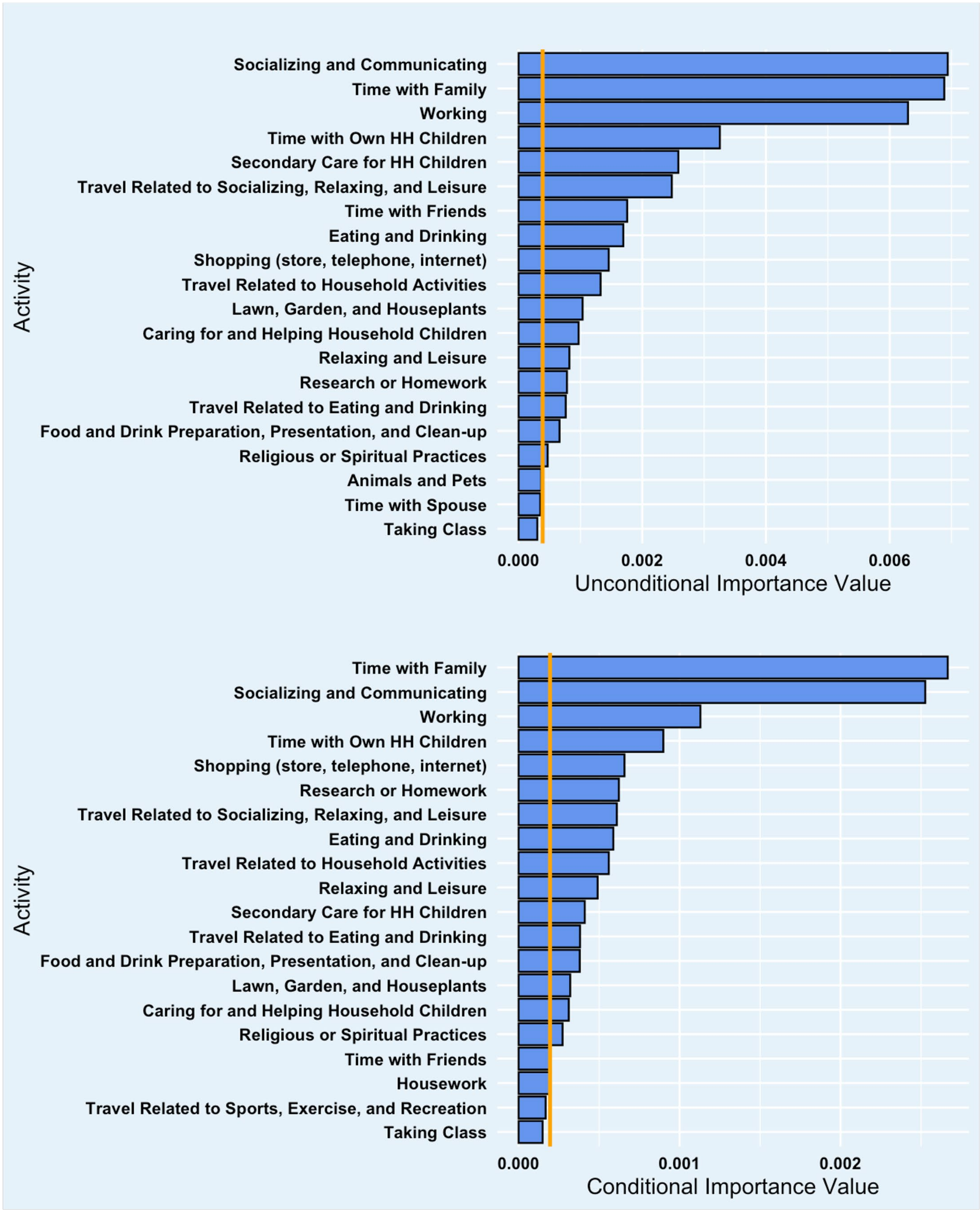

Figure S15. Top 20 variables by unconditional and conditional importance for 2021 weekend model.

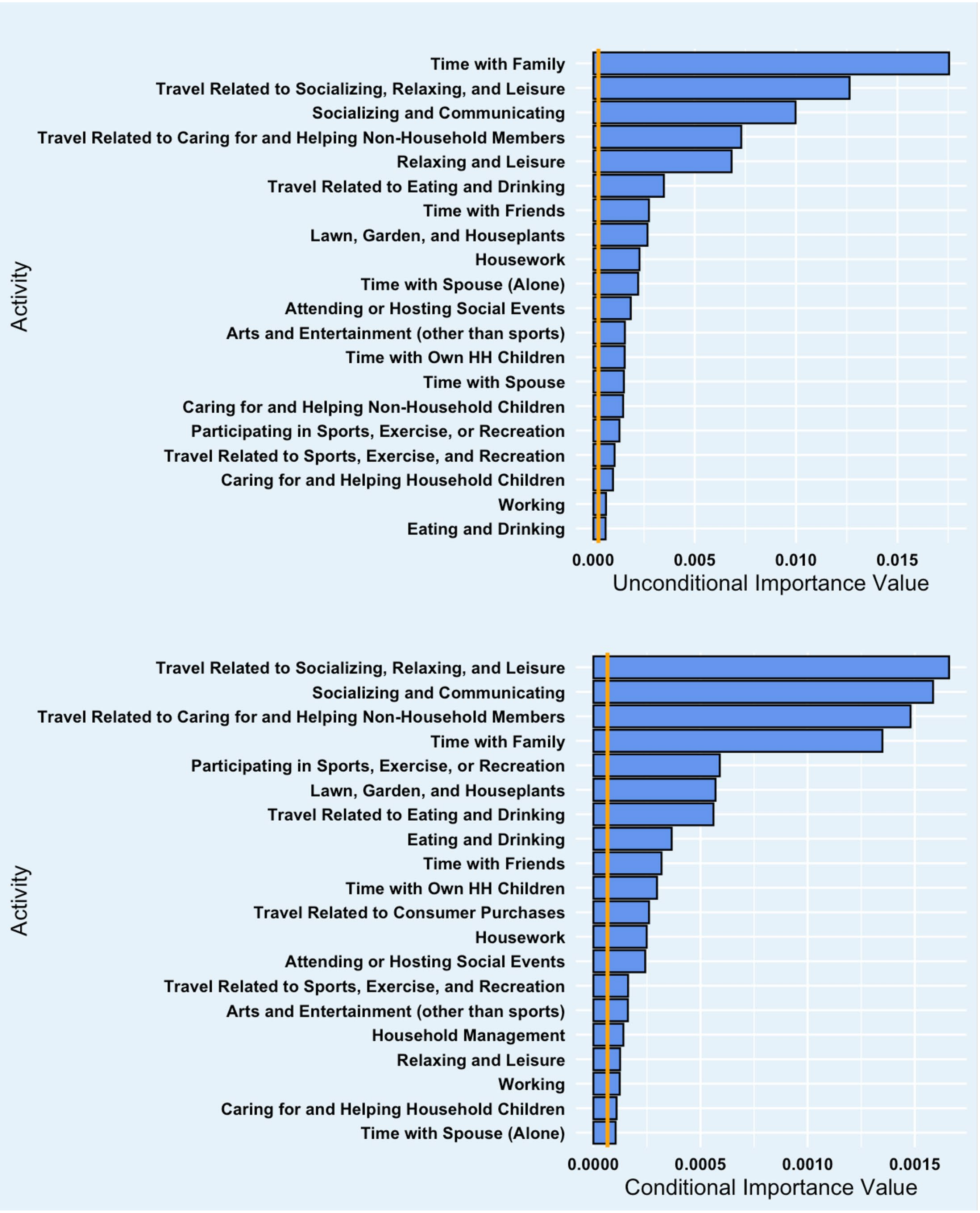

Figure S16. Top 20 variables by unconditional and conditional importance for 2013 weekday model.

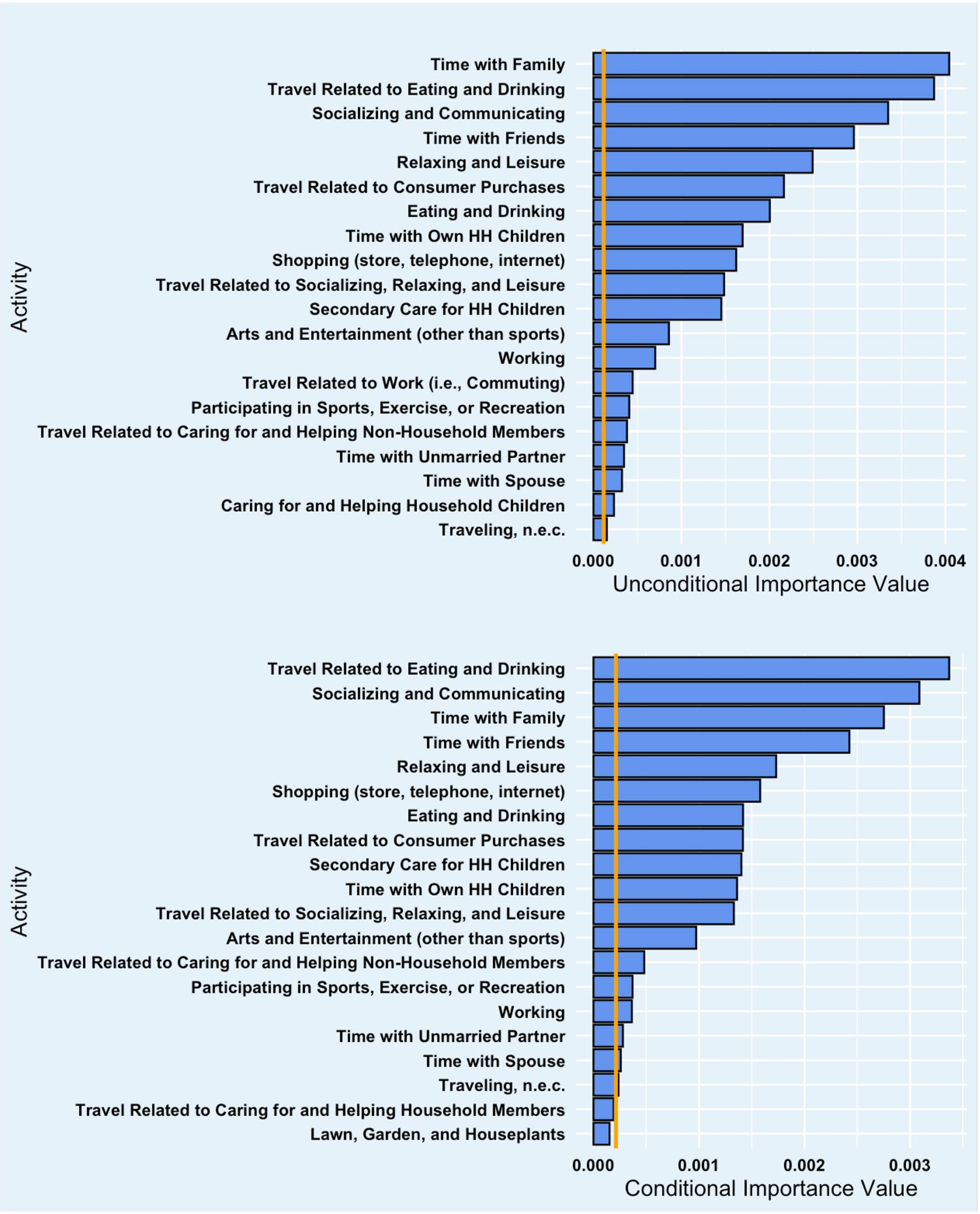

Figure S17. Top 20 variables by unconditional and conditional importance for 2013 weekend model.

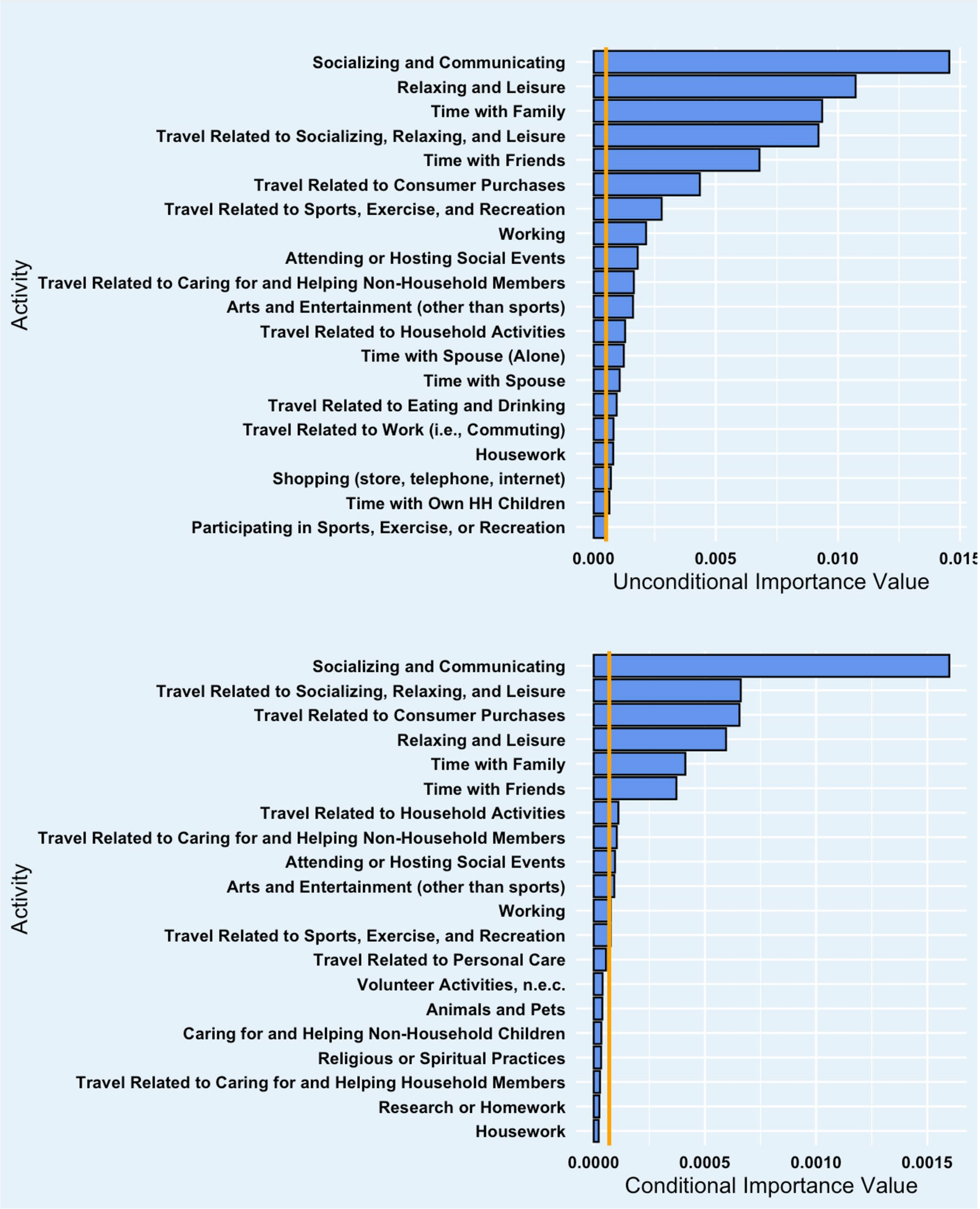

Appendix A: Data Dictionary for Time Use Portion of the ATUS (2013 and 2021)

This appendix provides the documentation provided the ATUS detailing the set of questions and metadata used in the ATUS survey aside from the well-being questions. Please see Appendix B and C for the well-being module information.

American Time Use Survey Questionnaire 2011-24

(Revised and condensed for public dissemination)

The American Time Use Survey is sponsored by the Bureau of Labor Statistics and conducted by the U.S. Census Bureau and is collected under OMB control #12200175 with an expiration of September 30, 2025.

About the ATUS Questionnaire

The American Time Use Survey (ATUS) is the first federally funded, continuous survey designed to measure time use in the United States. The ATUS is sponsored by the Bureau of Labor Statistics and conducted by the U.S. Census Bureau. Information is collected in the ATUS on how people spend their time, as well as where they are and whom they are with during their daily activities.

The ATUS is a nationally representative sample drawn from households that have completed their eighth and final interview for the Current Population Survey (CPS). Using CPS households saves time because demographic and labor force information has already been collected. An eligible person from each CPS household in the sample is selected as the ATUS designated person (DP). Each ATUS DP is then assigned a day about which to report his or her activities. The ATUS interviewer contacts the DP on the interview day, the day directly following the pre-assigned reporting day (e.g., Monday will be the interview day for a DP who has been assigned to report about their activities on a Sunday). ATUS interviewers attempt to contact the DP on the interview

day for up to eight weeks to complete a one-time interview. No proxy responses are allowed in the ATUS, so only the DP may complete the interview.

All of the ATUS interviews are conducted using a Computer Assisted Telephone Interviewing (CATI) instrument, allowing interviewers to easily follow complex skip patterns, skipping different questions depending on various respondent characteristics. For example, a person living alone will not be asked questions about his or her spouse. See the ATUS User's Guide (http://www.bls.gov/tus/atususersguide.pdf) for information about the interview and the CATI instrument.

One of the disadvantages of the CATI system is the loss of a readable paper questionnaire. The purpose of this document is to provide the data user with question universes (i.e., information about which respondents were asked the associated questions), question wording, response choices, and skip patterns in a readable paper format. This document includes most, but not all, questions asked in the survey in 2011 and later, their descriptions, and variables associated with the questions. These question variables should not be confused with the variable names provided in the ATUS data dictionaries. Information for each question is contained within individual boxes in this document. Text and selected screen shots are included to provide further explanation of the data collection instrument.

In 2011, questions about trips were replaced with eldercare questions. No other changes were made to the questionnaire. The 2003-10 ATUS questionnaire is available online at https://www.bls.gov/tus/questionnaires/tuquestionnaire0310.pdf.

S1: Introduction

The interview begins as the interviewer attempts to contact the designated person (DP) on the interview day. If the DP refuses the interview or someone else in the household refuses the interview on behalf of the DP, the interviewer records who refused and the reason for refusal. If the DP agrees to the interview, the interviewer verifies the DP's address.

HELLO

Universe Someone answers the telephone or someone calls into the telephone center

If outgoing call: Hello. This is _____ from the U.S. Census Bureau. May I please speak to [DPNAME]? **If incoming call:** Thank you for returning our call. My name is ______ from the U.S. Census Bureau. We contacted your house concerning a very important survey.

*If necessary: Am I speaking with [DPNAME]?

1. This is the correct person	[Go to INTRO]
2. Person not home or not available now	[Call back later]
3. Person unknown at this number	[Verify telephone number and address]
4. Other outcome or problem interviewing DP	[Terminate interview, record outcome]
Refused	[Go to S_REFWHO]

INTRO

Universe: All

We are conducting a survey for the Bureau of Labor Statistics on how people in America spend their time. This information will be used to find out how people balance work, child care, leisure, and other activities in their lives. This is a one-time only survey and will take about 20 minutes.

Did you receive our mailing for the American Time Use Survey explaining the importance of this survey and requesting your participation?

1. Yes [Continue with address verification]
2. No [Go to H_LETTER]
   Refusal [Go to S_REFWHO]

If the DP confirms receipt of the letter, the interview continues with address verification (below):

Before we get started, I need to verify that your address is still [ADDRESS]?

1. Same address [Go to INTRO_B]
2. Moved, not same address [Go to MOVED]
3. Haven't moved, but address has changed
4. Incorrect address previously recorded [Correct address, go to INTRO_B]

If the DP indicates that he or she has not received the letter, then the interviewer reads the following text (see H_LETTER below):

H_LETTER

Universe: INTRO = 2

As I mentioned, this survey will provide information about how people balance their time. I would like you to know that all the information you provide for this voluntary survey is confidential. This survey is authorized by Title 13, Sections 8 and 9. The OMB number is 1220-0175.

1. Return to INTRO and verify address

MOVED

Universe: Address verification in INTRO = 2

Since we selected an individual at this address for inclusion in the survey and we are not following people when they move, no interview is needed of you at this time. Thank you for your time.

1. Terminate interview and record outcome in interviewer notes

INTRO_B

Universe: INTRO = 1, 3, 4

This call may be recorded for quality assurance purposes. Do I have your permission to record this call? *If NO – respondent does not wish to be recorded, click on the stop recording button. Read if Necessary: I appreciate your concern. I am turning off the recording.

1. Continue	[Go to S2: (Household Roster)]
2. Inconvenient time. Callback needed.	[Schedule call back appointment]
3. Refused to participate	[Go to S_REFWHO]
4. Language problem or refer to supervisor	[Terminate interview, record outcome]

S_REFWHO

Universe: HELLO = Refused OR INTRO = Refused OR INTRO_B = 3

*Was the refusal BY the respondent or from someone else FOR the respondent?

If necessary ask: May I ask with whom I am speaking?

1. By DP
2. Parent/ guardian for the DP
3. Someone else
4. Not sure [Go to S_REFWHY]

S_REFWHY

Universe: S_REFWHO = 1-4

*Recode reason for refusal

If necessary ask: Would you please tell me the main reason you don't want to participate in this survey?

1. Tired of doing CPS
2. Not interested in topic
3. Too busy, survey takes too long
4. Topic is too personal/ none of government's business [Terminate interview, record outcome]
5. Other - Specify [Record verbatim response, terminate interview, record outcome]

S2: Household Roster

Universe: ALL

Next, the interviewer reviews the household roster, which includes the name, sex, birth date, and age of each household member, as well his or her relationship to the DP. The ATUS household roster begins with the last recorded CPS household roster. The interviewer verifies and updates the roster to reflect any changes in the household since the final CPS interview.

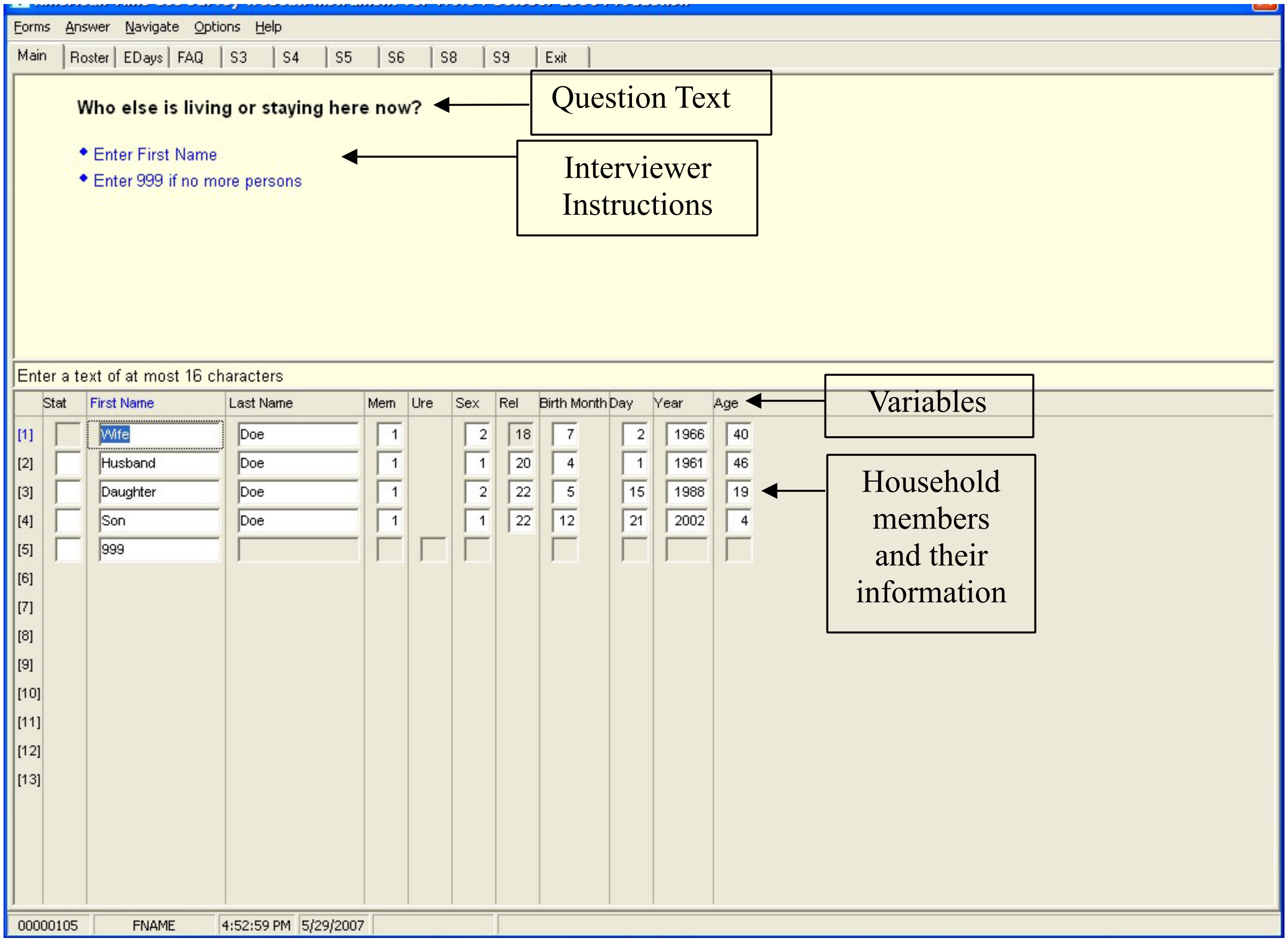

STLLIV

Universe: All

I have listed [Names]
* Read names

Do all these persons still live here?
1. Yes
2. No [Go to STATUS]

The following variables are included in the household roster (see screenshot above):

STATUS (Labeled STAT in screenshot above)

Universe: STLLIV = 1, 2 and Household member is not the DP

If STLLIV = 2: Use arrow key to move to the correct person and enter reason for membership change.
1. Person deceased
2. Person moved out
3. Person left - was a 'Usually Resides Elsewhere' last month
4. Correct a previous mistake
5. Person is a 'Usually Resides Elsewhere' this month
9. Reinstate

If there is a blank field on the roster, the interviewer runs through a series of questions to fill it in.

FNAME (Labeled FIRST NAME in screenshot above)

Universe: STATUS = blank, 9

Who else is living or staying here now?
1. Enter First Name
2. 999 if no more persons [Go to NHHCHILD]

LNAME (Labeled LAST NAME in screenshot above)

Universe: FNAME ≠ 999 and STATUS = blank, 9

1. Enter Last Name

MEM

Universe: FNAME ≠ 999 and STATUS = blank, 9

Is this [your/name's] usual place of residence?
* A nonhousehold child is a respondent's child under the age of 18 who does not live with the respondent
1. Yes [Go to SEX]
2. No [Go to URE]
3. No, nonhousehold child
Don't Know, Refused [Go to SEX]

URE

Universe: MEM = 2

Do you/ Does [FNAME] [LNAME] have a usual place of residence elsewhere?
1. Yes [Go to next household member]
2. No [Go to SEX]

SEX
Universe: MEM = 1, 2, 3, Don't Know or Refused and STATUS = blank, 9

What is [your/FNAME] [LNAME] sex?
1. Male 2. Female

Don't Know, Refused [IF DP or nonhousehold child, go to BIRTHM] [Else go to RRP]

RRP (Labeled REL in screenshot above)

Universe: (MEM = 1, 2, Don't Know, Refused and STATUS = blank, 9) AND (Person is not the DP)

How is [FNAME] [LNAME] related to you?
*Ask if not apparent
20. Spouse (husband/wife)
21. Unmarried partner
22. Child/stepchild
23. Grandchild
24. Parent (mother or father)
25. Brother/sister
26. Other related person (aunt, cousin, nephew, mother-in-law, etc.)
27. Foster child
28. Housemate/roommate
29. Roomer/boarder
30. Other nonrelative
40. Nonhousehold child [Go to BIRTHM]

BIRTHM (labeled Birth Month in screenshot above)

Universe: Person is household member and STATUS = blank, 9

What is [fill: your/name's] date of birth?
1. Enter Birth Month
Don't Know, Refused [Go to BIRTHD]

BIRTHD (Labeled DAY in screenshot above) Universe: BIRTHM = valid response

1. Enter Birth Day
Don't Know, Refused [Go to BIRTHY]

BIRTHY (Labeled YEAR in screenshot above)

Universe: BIRTHD = valid response

1. Enter Birth Year
Don't Know, Refused [Go to AGE]

AGE

Universe: BIRTHY

If BIRTHY = Response: As of yesterday, that would make [FNAME] [LNAME] approximately [AGE] years old. Is that correct?
IF BIRTHY = blank, Don't Know, Refused: Even though you don't know [FNAME] [LNAME]'s birth date, what is your best guess as to how old you/he/she were/was on your/his/her last birthday?
1. 0-98
Don't Know/Refused [Go to next line on grid]
[If last line on grid, go to NHHCHILD]

Before closing the roster screen, the interviewer verifies that no one else is currently living or staying in the household. If anyone new is added to the household roster, the series of questions beginning with FNAME above are repeated to capture his/her name, age, and relationship to the DP. In addition, respondents are asked whether they have any children under age 18 not living with them. If a respondent has a nonhousehold child under age 18, the child's information is added to the household roster, enabling the selection of that child in the WHO column once the ATUS diary is asked.

NHHCHILD

Universe: FNAME = 999 and all appropriate roster grid fields are complete

Do you have any children under 18 who do not live with you?
* Read if necessary: The way people spend time is often related to whether they have children even if the children don't live with them.
1. Yes, add child [Add person]

2. No [Go to S3: (Employment)]

S3: Employment

Universe: ALL

Next, the interviewer asks questions to determine whether the DP was working, looking for a job, on layoff, retired, or disabled. The interviewer also asks if anyone in the household owns a business or a farm. These questions update previously collected CPS information and use the same wording as the CPS questions except that the CPS language "last week" is changed to "last seven days" in the ATUS.

A CPS variable called the Monthly Labor force Recode (MLR) is used to drive the skip patterns in this section. MLR indicates whether the respondent was reported to be employed in the final CPS interview. Its values are as follows:

1 Employed, at work
2 Employed, absent
3 Unemployed, on layoff
4 Unemployed, looking
5 Not in labor force, retired
6 Not in labor force, disabled
7 Not in labor force, other

RRP (See S2: Household Roster) is also used to constrain the universe for several variables. RRPs of 20 and 21 constrain the universe to persons with a spouse (20) or unmarried partner (21).

LABFOR

Universe: All

We know that people who work spend their time differently than others. So, before I ask you about what you did yesterday, I need to update your employment status. The next few questions will sound familiar to you if you were the person in your household who did the Current Population Survey. I'm going to ask you about work in the LAST SEVEN DAYS. By the LAST SEVEN DAYS, I mean the days beginning on [Interview date minus eight, in day, month, date, year format], and ending yesterday.

1. Enter 1 to continue. [Go to BUS]

BUS

Universe: All

[Do you/Does anyone in this household] have a business or a farm?
1. Yes [If there is only 1 household member, go to FWK] [Else go to BUSL1]
2. No

Don’t Know, Refused [Go to FWK]

BUSL1

Universe: BUS =1 AND more than 1 household member

Whose business or farm is it?

*Enter all that apply, separate with commas.

(The instrument displays all household members, and instructs the interviewer to enter the number of each person who owns the business or farm, separated by a comma.)

1. [FNAME] [LNAME]
2. [FNAME] [LNAME]
3. [FNAME] [LNAME]

Don’t Know, Refused [Go to FWK]

FWK

Universe: All

In the LAST SEVEN DAYS, did you do ANY work for [pay/either pay or profit]?

1. Yes [Go to MJ]
2. No [If BUS=1 and there is only 1 household member go to BUS1]
   [If MLR = 5 and age > 64 AND FWK = 2, 4, or 5 go to RET]
   [If MLR = 6 go to DIS]
   [All others go to FABS]
3. Retired [Age > 64 go to RET1a]
4. Disabled [Go to DIS1a] 5. Unable to work [Go to DIS2a]

Don’t Know, Refused [Go to FABS]

BUS1

Universe: FWK = 2-5 AND BUS = 1 AND more than one household member

In the LAST SEVEN DAYS, did you do any unpaid work in the family business or farm?

1. Yes [If a household member is a business owner go to HRUSL1] [Else go to BUS2]
2. No
   [If MLR = 5 and age > 64 AND FWK = 2, 4, or 5 go to RET]
   [If MLR = 6 go to DIS]
   [If FWK = 4 go to DIS1a]
   [If FWK = 5 go to DIS2a]
   [If FWK = 3 AND age > 64 go to RET1a]
   [All else go to FABS]

BUS2
Universe: **BUS1 = 1 AND household member other than DP or DP's spouse/unmarried partner is/are the business owner(s)**

Do you receive any payments or profits from the business?
1. Yes 2. No

Don't Know, Refused [Go to HRUSL1]

DIS
Universe: **(FWK = 2-5 AND (BUS ≠ 1 OR there is more than one household member) OR (BUS1 = 2, Don't Know, Refused)) AND MLR = 6**

[Fill: Last time we spoke to someone in this household, you were reported to have a disability.]
Does your disability continue to prevent you from doing any kind of work for the next 6 months [fill: including work in the family business or farm]?

1. Yes [If RRP = 20, 21 for any household member go to SP1]
[Else go to S4: (Time-use Diary)]
2. No
3. Did not have disability last month
Don't Know, Refused [If FWK = 3 AND age > 64 go to RET1a] [Else go to FABS]

RET1a
Universe: **(FWK = 3 AND (BUS ≠ 1 or there is more than one household member) and age > 64 and (DIS ≠ 1))**
OR (BUS1 = 2, Don't Know, Refused and FWK = 3 and age > 64)
OR (RET = 1)

Do you currently want a job, either full or part time?
1. Yes, Maybe, it depends
2. No [If RRP = 20, 21 for any household member go to SP1]
[Else go to S4: (Time-use Diary)]
3. Has a job [Go to ABSRSN]
Don't Know, Refused [If RRP = 20, 21 for any household member go to SP1]
[Else go to S4: (Time-use Diary)]

DIS1a
Universe: **(DIS = 2, 3, Don't Know, Refused AND FWK =3 AND age > 64)**
OR (FWK = 4 AND (MLR ≠ 5 or age < 50) AND MLR ≠ 6 AND (BUS ≠ 1 or there is more than one household member))
OR (BUS1 = 2, Don't Know, Refused AND (FWK = 4 AND (MLR ≠ 5 or age < 50)
AND MLR ≠ 6)

Does your disability prevent you from accepting any kind of work during the next six months?

1. Yes [If RRP = 20, 21 for any household member go to SP1] [Else go to S4: (Time-use Diary)]
2. No

Don't Know, Refused [Go to FABS]

DIS2a

Universe: (FWK = 5 AND (MLR ≠ 5 or age < 50) AND MLR ≠ 6 AND (BUS ≠ 1 or there is more than one household member)) OR BUS1 = 2, Don't Know, Refused AND FWK = 5 AND ((MLR ≠ 5 or age < 50) AND MLR ≠ 6)

Do you have a disability that prevents you from accepting any kind of work during the next six months?

1. Yes [If RRP = 20, 21 for any household member go to SP1] [Else go to S4: (Time-use Diary)]
2. No [Go to FABS]

FABS

Universe: (RET1a = empty) AND [(WK = 2) AND (BUS ≠ 1 or more than one household member) AND (MLR ≠ 5) or (age < 50) AND (MLR ≠ 6)]
OR [(FWK = 3) AND (age < 50) AND (BUS ≠ 1 or more than one household member) AND (MLR ≠ 5) AND (MLR ≠ to 6)] OR
[(BUS1 = 2, Don't Know, Refused) AND (FWK = 2) AND (MLR ≠ 5) or (age < 50) AND (MLR ≠ 6)]
OR [(BUS1 = 2, Don't Know, Refused) AND (FWK = 3) AND (age < 50) AND (MLR ≠ 6)]
OR [(RET = 2, 3, Don't Know, Refused AND (FWK ≠ 3 or age < 50)]
OR [(DIS = 2, 3, Don't Know, Refused AND (FWK ≠ 3 or age < 50)]
OR (DIS1a = 2, Don't Know, Refused)
OR (DIS2a = 2, Don't Know, Refused)

OR (FWK = Don't Know, Refused)

In the LAST SEVEN DAYS, [in addition to the business,] did you have a job either full or part time? Include any job from which you were temporarily absent.

1. Yes [Go to ABSRSN]
2. No [If a household member is a business owner, go to ABSRSN] [Else go to LAY]
3. Retired [If age > 64 AND no entry in RET1a go to RET1b] [Else go to LAY]
4. Disabled [Go to DIS 1b]
5. Unable [Go to DIS 2b]

Don't Know, Refused [Go to LAY]

RET1b

Universe: (No entry in RET1a) AND FABS = 3 AND age > 64

Do you currently want a job, either full or part time?

1. Yes or Maybe, it depends

2. No [If RRP = 20, 21 for any household member go to SP1]
[Else go to S4: (Time-use Diary)]
3. Has a job [Go to ABSRSN]
Don't Know, Refused [If RRP = 20, 21 for any household member go to SP1]
[Else go to S4: (Time-use Diary)]

DIS1b

Universe: FABS = 4

Does your disability prevent you from accepting any kind of work during the next six months?
1. Yes [IF RRP = 20, 21 for any household member go to SP1]
[Else go to S4: (Time-Use Diary)]
2. No
Don't Know, Refused [Go to LAY]

DIS2b

Universe: FABS = 5

Do you have a disability that prevents you from accepting any kind of work during the next six months?
1. Yes [If RRP = 20, 21 go to SP1]
[Else go to S4: (Time-use Diary)]
2. No
3. Don't Know, Refused [Go to LAY]

LAY

Universe: (DIS1b = 2, Don't Know, Refused)
OR (DIS2b = 2, Don't Know, Refused)
OR (FABS = Don't Know, Refused)
OR (FABS =2 AND No household member is a business owner)
OR (FABS = 3 AND entry in RET1a or age < 25)

During the LAST SEVEN DAYS, were you on layoff from a job?
1. Yes
2. No [If RRP =20, 21 for any household member go to SP1]
[Else go to S4 (Time-use Diary)]
3. Retired [If age > 64 go to RET1c]
[If RRP = 20, 21 for any household member go to SP1]
[Else go to S4: (Time-use Diary)]
4. Disabled [Go to DIS1c]
5. Unable [Go to DIS2c]
Don't Know, Refused [If RRP =20, 21 for any household member go to SP1]
[Else go to S4: (Time-use Diary)]

RET1c

Universe: (No entry in RET1 AND RET1a) AND LAY = 3 AND age > 64

Do you currently want a job, either full or part time?
1. Yes or Maybe, it depends
2. No [If RRP = 20, 21 for any household member go to SP1]
[Else go to S4: (Time-use Diary)]
3. Has a job [Go to ABSRSN]
Don't Know, Refused [If RRP = 20, 21 for any household member go to SP1]
[Else go to S4: (Time-use Diary)]

DIS1c
Universe: (No entry in DIS1a, DIS2a, DIS1b and DIS2b) AND LAY = 4

Does your disability prevent you from accepting any kind of work during the next six months?
1. Yes 2. No

Don't Know, Refused [If RRP = 20, 21 for any household member go to SP1]
[Else go to S4: (Time-use Diary)]

DIS2c
Universe: (No entry in DIS1a, DIS2a, DIS1b and DIS2b) AND LAY = 5

Do you have a disability that prevents you from accepting any kind of work during the next six months?
1. Yes
2. No
Don't Know, Refused [If RRP = 20, 21 for any household member go to SP1]
[Else go to S4: (Time-use Diary)]

ABSRSN
Universe: **(FABS = 1)**
OR (RET1a = 3)
OR (RET1b = 3)
OR (RET1c = 3)

OR (FABS =2 AND (a household member is a business owner))

What was the main reason you were absent from work during the LAST SEVEN DAYS?

1. On layoff (temporary or indefinite)
2. Slack work/business conditions
3. Waiting for new job to begin [If RRP = 20, 21 for any household member go to SP1] [Else go to S4: (Time-use Diary)]
4. Vacation/personal days
5. Own illness/injury/medical problems
6. Childcare problems
7. Other family/personal obligation
8. Maternity/paternity leave
9. Labor dispute
10. Weather affected job
11. School/training
12. Civic/military duty [If a household member is a business owner and FABS = 1, 2 go to HRUSL1] [Else go to MJ]
13. Does not work in the business [If RRP = 20, 21 for any household member go to SP1] [Else go to S4: (Time-use Diary)]
14. Other [Go to ABSPC]

Don't Know, Refused [If a household member is a business owner and FABS = 1, 2 go to HRUSL1] [Else go to MJ]

ABSPC
Universe: **ABSRSN = 14**

1. Enter Verbatim Response

Don't Know, Refused

[If a household member is a business owner and FABS = 1, 2 go to HRUSL1] [Else go to MJ]

MJ
Universe: **FWK = 1**
OR (ABSRSN = 4 – 12, 14, Don't Know, Refused
AND (no household member is a business owner)) OR (FABS ≠ 2)

In the LAST SEVEN DAYS, did you have more than one job [or business], including part-time, evening or weekend work?

1. Yes 2. No

Don't Know, Refused [go to HRUSL1]

HRUSL1

Universe: (BUS1 = 1 AND a household member is a business owner)
OR (MJ = 1, 2, Don't Know, Refused)
OR (BUS2 = 1, 2, Don't Know, Refused)
OR (ABSRSN = 4-12, 14, Don't Know or Refused

AND (a household member is a business owner) AND FABS = 1, 2)

How many hours per week do you USUALLY work at your [main] job?

*By main job we mean the one at which you usually work the most hours.

*Please enter Hours or Enter V if Hours Vary

0-99 hours [If MJ = 1 go to HRUSL2]
[If RRP = 20, 21 for any household member go to SP1]
[Else go to S4: (Time-use Diary)]

Hours Vary

Don't Know, Refused [BUS1 = 1 and if DP has only one job, go to HRFTPT]
[If RRP = 20, 21 for any household member go to SP1]
[Else go to S4: (Time-use Diary)]

HRUSL2

Universe: HRUSL1 = valid response AND DP has more than one job

How many hours per week do you USUALLY work at your other job(s)?

* Please enter Hours or Enter V if Hours vary

0-99 hours

Hours vary

Don't Know, Refused [If HRUSL1 = Don't Know, Refused, Hours vary, 0-34 and
HRUSL2 =Don't Know, Refused, Hours vary, go to HRFTPT]
[If HRUSL2 = Don't Know, Refused, Hours vary, 0-34 and
HRUSL1 = Don't Know, Refused, Hours vary, go to HRFTP]
[If RRP = 20, 21 for any household member go to SP1] [Else go to S4: (Time-use Diary)]

HRFTPT

Universe: (HRUSL1 = Don't Know, Refused, Hours Vary AND (BUS =1 or DP has only 1 job))
OR (HRUSL2 = Don't Know, Refused, Hours Vary AND HRUSL1 = Don't Know, Refused, Hours Vary, 0-34)
OR (HRUSL1 = Don't Know, Refused, Hours vary AND HRUSL2 = Don't Know, Refused, Hours vary, 0-34)

Do you USUALLY work 35 hours or more per week [at all your jobs combined/in the family business or farm/ at your job]?

1. Yes
2. No
3. Hours vary

Don't Know, Refused [If RRP = 20, 21 for any household member go to SP1]
[Else go to S4: (Time-use Diary)]

SP1
Universe: **Household member with RRP = 20, 21 who was not deleted from household roster because he/she moved out, or was deceased**

In the LAST SEVEN DAYS, did [spouse's name] do ANY work for [pay/ either pay or profit]?
1. Yes [Go to SP3]
2. No [Go to SP2]
3. Retired [Go to S4: (Time-use Diary)]
4. Disabled [Go to S4: (Time-use Diary)] 5. Unable to Work [Go to S4: (Time-use Diary)]

Don't Know, Refused [Go to SP2]

SP2
Universe: **SP1 = 2, Don't Know, Refused**

In the LAST SEVEN DAYS, [in addition to the business,] did [spouse's name] have a job either full or part time? Include any job from which [he/she] was temporarily absent.
1. Yes [Go to SP3]
2. No
3. Retired
4. Disabled 5. Unable

Don't Know, Refused [Go to S4: (Time-use Diary)]

SP3

Universe: SP2 = 1

How many hours per week does [spouse's name] USUALLY work?
*Enter V for Hours vary

00-99 hours [Go to S4: (Time-use Diary)]
Hours vary
Don't Know, Refused [Go to SP4]

SP4

Universe: SP3 = Hours vary, Don't Know, Refused

Does [he/she] USUALLY work 35 hours or more per week?
1. Yes

2. No
3. Hours vary
4. No longer has a job
Don’t Know, Refused [Go to S4: (Time-use Diary)]

S4: Time-use Diary

Universe: ALL

Next, the interviewer collects a detailed account of the DP’s activities from 4 a.m. the previous day to 4 a.m. on the interview day. The interviewer uses pre-codes (1-12 in the diary grid) to quickly record commonly-reported activities, but records the DP’s verbatim responses for all other activities. The interviewer then asks how long each reported activity took. This may be recorded either as the duration of the activity or as the start and stop times of the activity. The interviewer continues, asking WHO questions for all activities, except for sleeping, grooming, and personal activities (e.g. cuddling, making out, etc.). He or she then asks the WHERE questions for all activities except for sleeping, grooming, and personal activities. After the DP completely answers all questions about an activity (including duration, with whom, and where), the interviewer prompts for the next activity.

The ATUS does not collect simultaneous activities. If a respondent reports doing more than one activity at a given time, the interviewer first asks her if she can separate the activities into different time intervals. If she is unable to do this, the interviewer asks her which activity was her main activity and records the response.

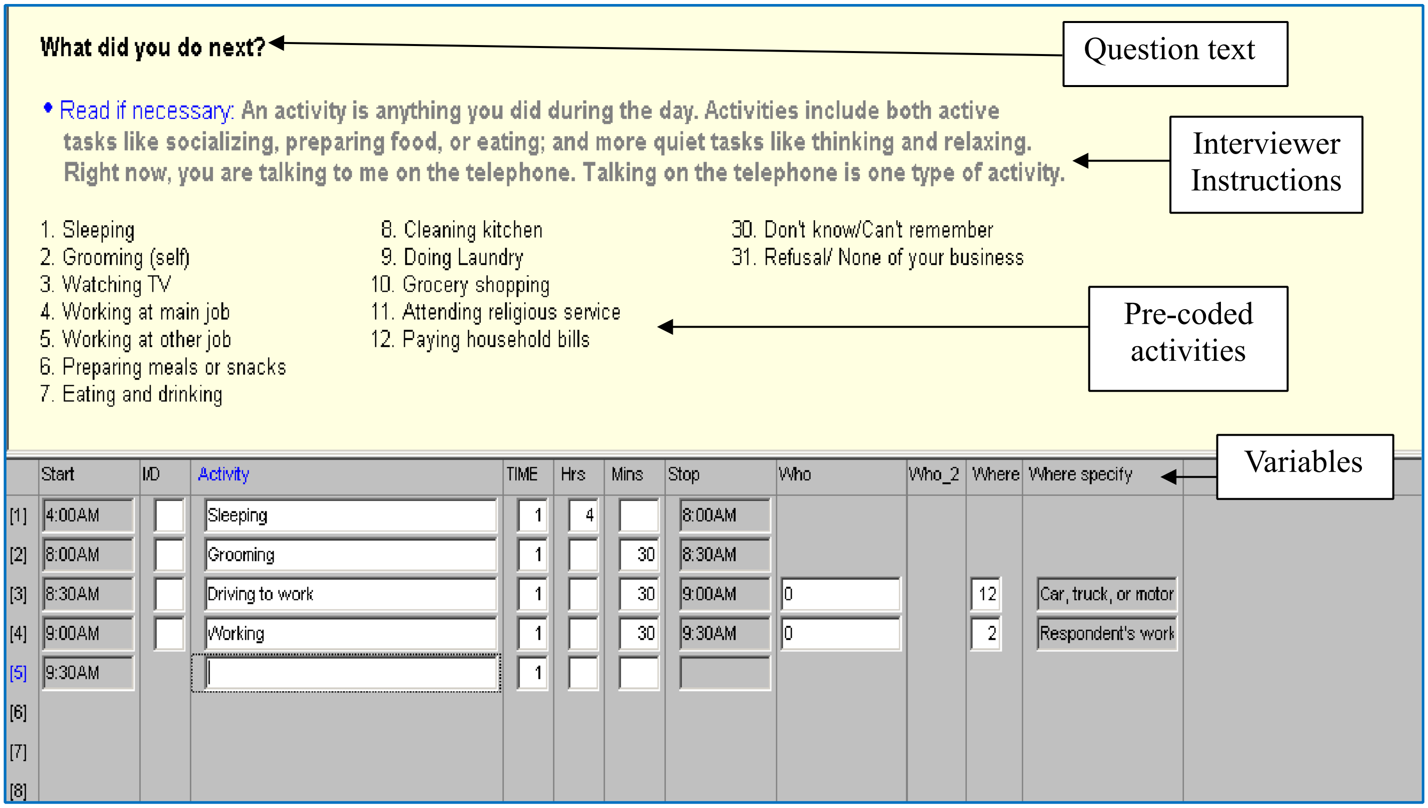

CORE_LEAD

Universe: All

Now I'd like to find out how you spent your time yesterday, [yesterday's day & date], from 4:00 in the morning until 4:00 AM this morning. I'll need to know where you were and who else was with you. If an activity is too personal, there's no need to mention it.

The following variables are included in the diary grid:

ACTIVITY

Universe: All

So let's begin. Yesterday, [previous weekday] at 4:00 AM, what were you doing? /What did you do next?

(If the DP reports an activity with no associated precode, the interviewer can type the activity directly onto the blank activity line.)

1. Sleeping
2. Grooming (self)
3. Watching TV
4. Working at main job
5. Working at other job
6. Preparing meals or snacks
7. Eating and drinking
8. Cleaning kitchen
9. Laundry
10. Grocery shopping
11. Attending religious service
12. Paying household bills
30. Don't know/ Can't remember
31. Refusal/ None of your business [Go to TIME]

TIME

Universe: ACTIVITY = valid response

How long did you spend [ACTIVITY]?

1. Enter duration (hours, minutes) [Go to HOURDUR]
2. Enter stop time [Go to STOPTIME]

HOURDUR
Universe: **TIME=1**

Enter Hours [Go to MINDUR]

MINDUR
Universe: **TIME =1 AND HOURDUR=blank or valid entry**

Enter Minutes [Go to STOPTIME]

STOPTIME

Universe: All

*Instrument will calculate STOPTIME if HOURDUR and MINDUR have entries other than blank or Don't Know.
*If there is a value for STARTIM, then interviewer should do the following:

Enter Time and AM or PM

WHO

Universse: Not a personal activity and not an activity with a precode of 1, 2, 30, 31*

Who was with you? / Who accompanied you?
0. Alone
1—39. Household members and nonhousehold children
50. All household members
51. Parents
52. Other non-HH family members <18
53. Other non-HH family members 18 and older (including parents-in-law)
54. Friends
56. Neighbors, acquaintances
57. Other non-HH children < 18
58. Other non-HH adults 18 and older (including parents-in-law)
59. Boss or manager*
60. People whom I supervise*
61. Co-workers*
62. Customers* [Go to WHERE]

*Note: WHO was not asked about work activities (corresponding to precodes 4 and 5) until January 2010. Response options 59-62 were added to the WHO question at this time.

WHERE

**Universe:**	**Personal activity reported OR ACTIVITY ≠ Precodes 1, 2, 30, 31**	
Where were you while you were [ACTIVITY]?		
PLACE		MODE OF TRANSPORTATION
1. DP's home or yard	30. Bank*	12. Car, truck, or motorcycle (driver)
2. DP's workplace	31. Gym/ Health Club*	13. Car, truck, or motorcycle (passenger)
3. Someone else's home	32. Post Office*	14. Walking
4. Restaurant/Bar		15. Bus
5. Place of worship		16. Subway/Train
6. Grocery store		17. Bicycle
7. Other store/Mall		18. Boat/Ferry
8. School		19. Taxi/Limousine Service
9. Outdoors away from home		20. Airplane
10. Library		21. Other (specify)
11. Other place (specify)		
		[If STOPTIME > 4 AM, go to S5: (Summary questions)]<br>[Else continue to next row]

*Note: Response options 30-32 were added to WHERE in mid-2004.

The following screenshots demonstrate how the WHO and WHERE questions are collected in the instrument:

WHO Screenshot

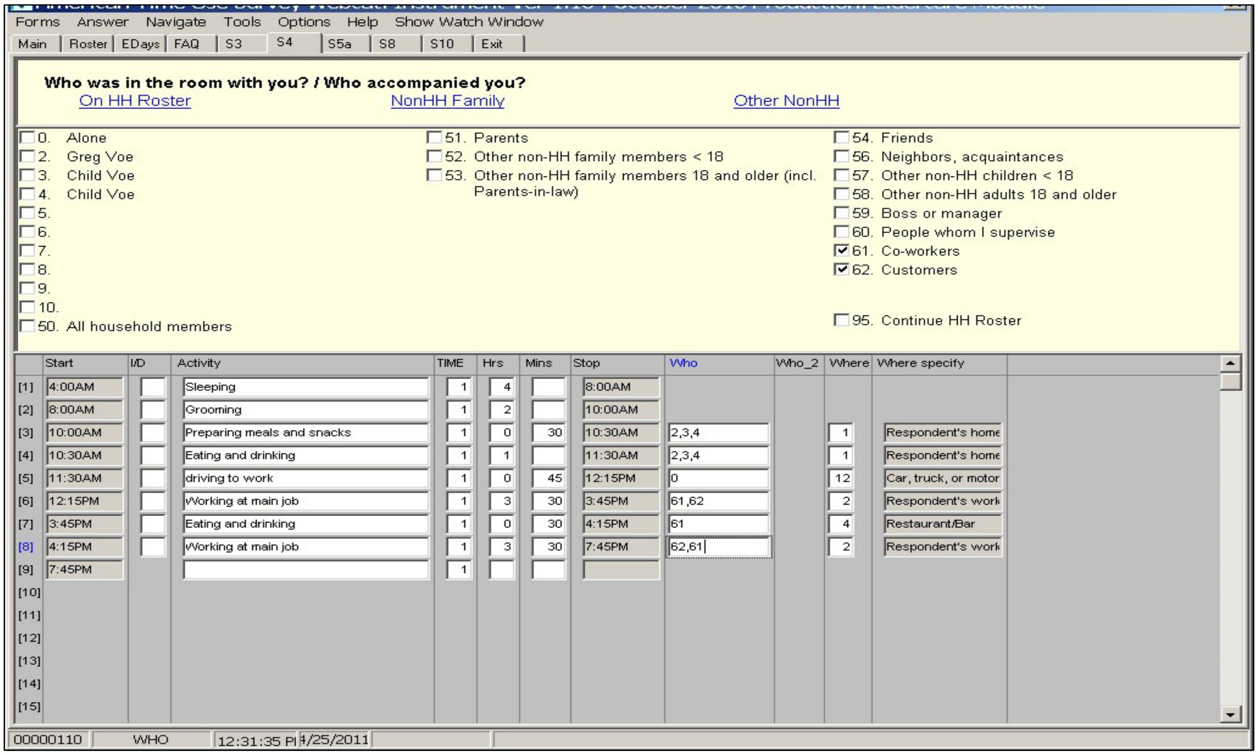

WHERE Screenshot

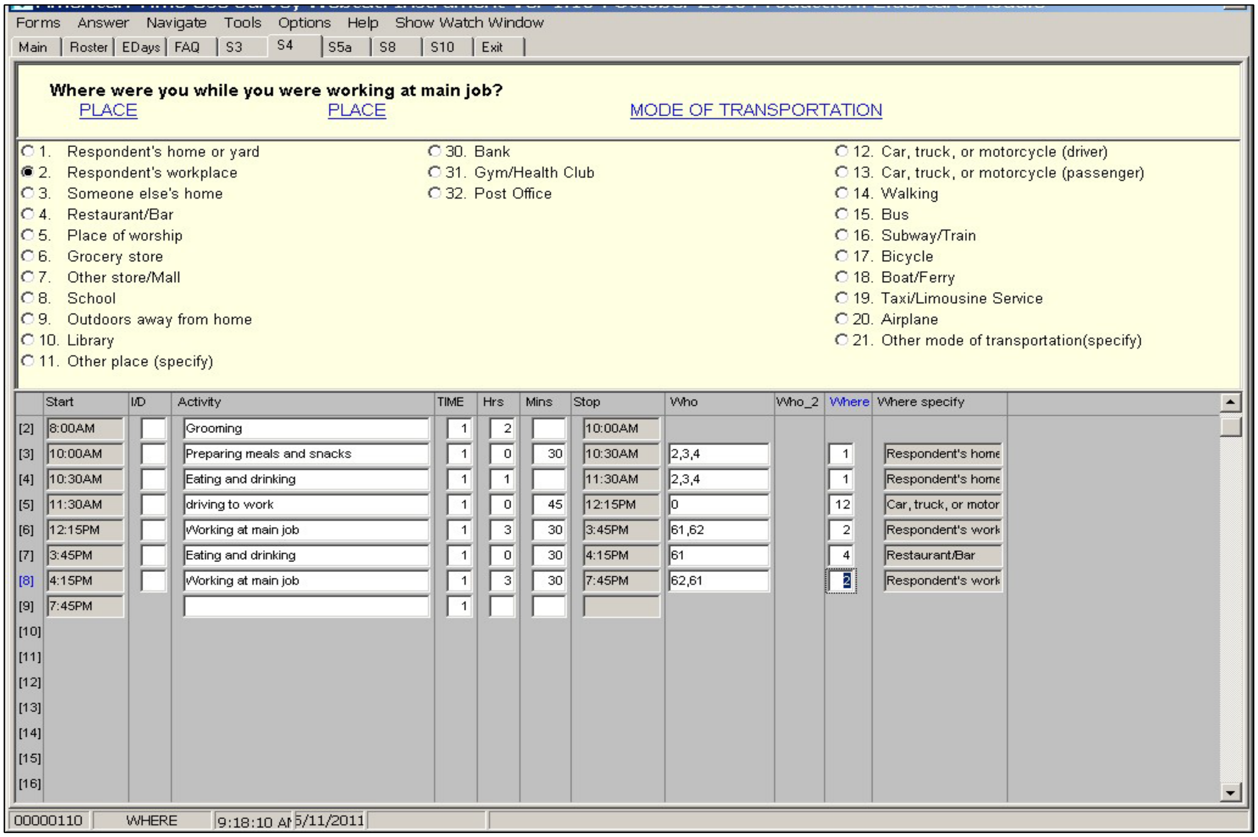

The following variable is not included in the diary grid. This question is asked only if the respondent did not report any eating or drinking as a main activity for the 24-hour reporting day.

EATCK

Universe: ACTIVITY ≠ Precode 7

You did not report any eating or drinking yesterday. Did you do any eating or drinking yesterday as your main activity?<br>1. Yes [Edit diary, go to S5: (Summary questions)]<br>2. No [Go to S5: (Summary questions)]

S5: Summary Questions

Universe: Time-use Diary and Employment are complete

After the time diary is complete, the interviewer asks follow-up "summary questions" to obtain additional information on secondary childcare, volunteer, and work and income-generating activities. These series of questions are explained in the sequence that they appear in the instrument (below).

Work and Income-generating Activities

During the diary portion of the interview, work activities reported as such are captured in a straightforward way. The interviewer usually uses a pre-code of 4 or 5, the instrument automatically filling the field with “work, main job” or “work, other job” as indicated. However, some responses are not obviously work or income-generating activities, such as “reviewing tax documents.” The summary questions below help to clarify which activities should be coded as work or income-generating. Any other activities identified by the respondent as work or incomegenerating are marked with a “1” on the diary grid (see screenshot).

The interviewer first asks the respondent to identify any additional work activities that were done for his or her job or business. If a respondent has more than one job, the question is asked about both his or her main job, and his or her other job(s). Then, the interviewer asks about other activities done to earn additional income, such as hobbies done for income.

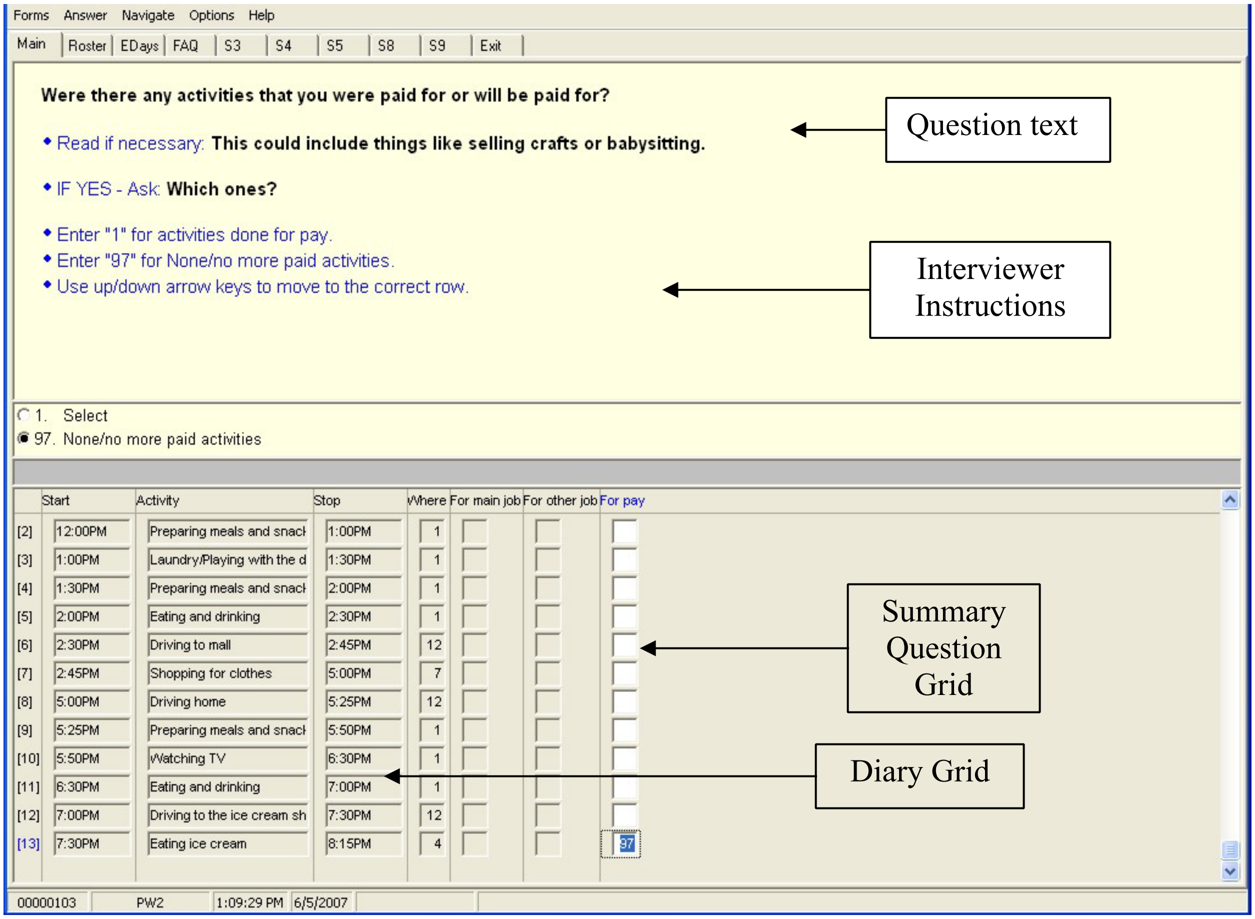

In this section, skip patterns for some questions are driven by responses to the MJ variable (see S3: (Employment)), indicating whether respondents have only one job (MJ=2) or more than one job (MJ=1).

PW1
Universe: **(MJ ≠ 1) AND (DP worked some hours during the week)**

We are interested in measuring the amount of time people spend working both inside and outside their usual workplace. You said that you were working from [start and stop time of worked at main job from time diary] Were there any [other] activities that were done as part of your job or business?
Please do not include getting ready for work or commuting.
1. Activities done for job. [Next line of grid][If no more entries go to PW2]
97. None/no more work activities. [Go to PW2]
Don't Know, Refused [Next line of grid] [If no more entries go to PW2]

PWMJ1

Universe: MJ = 1

We want to analyze how much time people commit to work both inside and outside their place of business. Of all the activities you mentioned, which were done as part of your MAIN job [business]? Please do not include getting ready for work or commuting.
1. Activities done for main job. [Next line of grid] [If no more entries go to PWOJ1]
97. None/no more work activities. [Go to PWOJ1]
Don't Know, Refused [Next line of grid] [If no more entries go to PWOJ1]

PWOJ1
Universe: **MJ = 1**

You said that you were working for your OTHER job from [start and stop time of worked at other job from time diary]. Were any of the [other] activities you mentioned done as part of your OTHER job [or business]?
1. Activities done for other job. [Next line of grid] [If no more entries go to PW2] 97.
None/no more work activities. [Go to PW2]
Don't Know, Refused [Next line of grid] [If no more entries go to PW2]

PW2
Universe: **All**

If HRUSL1 is empty: Sometimes people do things that bring in money like selling crafts or babysitting. Were there any activities that you did yesterday that you were paid for or will be paid for?

If PW1, PWMJ1, or PWOJ1 = 1: You told me about the activities that were done as part of your job(s). Were there any other activities that you were paid for or will be paid for? Please do not include paid breaks at work or paid time off.
* Read if necessary: This could include things like selling crafts or babysitting.
IF YES - Ask: Which ones?
1. Activities done for pay. [Next line of grid] [If last line, go to CC_LEAD]
97. None/no more paid activities. [Go to CC_LEAD]
Don't Know, Refused [Next line of grid] [If last line, go to CC_LEAD]

Secondary Childcare

Next, the interviewer asks questions to obtain information on secondary childcare, defined as occurring when the DP had a child under age 13 in his or her care while doing other activities. The interviewer also asks what time the first child under age 13 got up and what time the last child under 13 went to bed.

In 2003, secondary childcare activities performed by the DP were captured separately for 1) household and own nonhousehold children under 13, and 2) non-own nonhousehold children under 13. After 2003, universes to the questions were altered so that separate measures could be developed for time the DP spent providing secondary childcare to 1) own household children, 2) non-own household children, 3) own nonhousehold children, and 4) non-own nonhousehold children. Because the questions during and after 2003 were similar in structure, with differences only in the universes, they are shown below only as they were asked in 2004 and after.

CC_LEAD

Universe: All

If household roster includes children under 13 years of age: Now I'd like to talk with you in a little more detail about childcare.

1. Enter 1 to continue [If at least two household children < 13 then go to CC1]
[If one household child < 13 then go to CC2]

OR

If household roster does not include children under 13 years of age: Now I'd like to talk with you about childcare. People often spend time with friends', neighbors' or relatives' children.

1. Enter 1 to continue. [If no household children < 13 and at least 1 nonhousehold child < 13 then go to CC6]
[Else go to CC8]

CC1

Universe: At least 2 household children < 13

I'd like you to think back over the day yesterday. Which child got up first yesterday?
*Display all names of household children in universe
*Read names, select all that apply separated by commas

1. [FNAME] [LNAME]
2. [FNAME] [LNAME]
3. [FNAME] [LNAME] [Go to CC2]

Don’t Know, Refused [Go to CC3]

CC2

Universe: (CC1 ≠ Don’t Know, Refused) OR (only one household child <13)

At what time, yesterday, did [FNAME] [LNAME] get up?

1. Time in HH:MM format
   Don’t Know, Refused [If 2 or more children < 13 listed in WHO column on time diary, go to CC3]
   [If 2 or more children < 13 listed in WHO column on time diary and CC2 =value with PM go to CC_CK]
   [If 1 child < 13 listed in WHO column on time diary, go to CC4]

CC3

Universe: (CC1= Don’t Know, Refused) OR [(CC2 = Valid response) AND (At least 2 household children <13)]

Which child or children went to bed last?
*Display names of household children in the universe
*Read names, select those that apply, separated by commas

1. [FNAME] [LNAME]
2. [FNAME] [LNAME]
3. [FNAME] [LNAME] [Go to CC4]

Don’t Know, Refused [If at least one own household child <13, go to CC5]
[Else if no own household child <13, but at least one non-own household child, then go to CC5B]

CC4

Universe: CC3 ≠ Don’t Know, Refused OR (At least 1 household child <13)

At what time did [FNAME] [LNAME] go to bed?
1. Time in HH:MM format [If CC2 = value with AM go to CC_CK]
[If at least one own household child <13, go to CC5]
[Else if no own household child <13, but at least one non-own household child, then go to CC5B]

CC5
Universe: At least 1 OWN household child under 13

I'D LIKE TO ASK YOU ABOUT CHILDREN WHO LIVE WITH YOU. A child was awake between [insert value from CC2] and [insert value from CC4]. At which times or during which activities during that time period was/were [FNAME] [LNAME] in your care? (fills name(s) of all the DP's own children under 13 in the household)

* Probe: Any other times or activities?

1. Activities where child was in your care. [Go to next row]

96. All day.

97. None/no more childcare activities. [If first row of CC5=97, or if no entries in CC5, go to CC5_CK]

Don't Know, Refused [If CC5= Don't Know, Refused, go to next row]

[If no more rows or if CC5=96, 97 and at least one non-own household child< 13, then go to CC5B]

[Else if no more rows or if CC5=96, 97and no own nonhousehold children, go to CC8]

CC5_CK
Universe: If first row of CC5=97 or no entries in CC5

The interviewer may clarify with the respondent that:

*No activities were selected even though there are own children in the household <13.
*Enter reason or use the arrow key to go back to correct

1. No secondary childcare activities
2. Respondent didn't know
3. Respondent refused to answer
4. Child was away from home yesterday
5. Respondent was away from home yesterday [If at least 1 non-own household child <13, then go to CC5B]

[Else if at least 1 own nonhousehold child < 13, then go to CC6]

[Else if no own nonhousehold children <13, then go to CC8]

CC5B

Universe: At least 1 NON-OWN household child under 13

NOW I'D LIKE TO ASK YOU ABOUT OTHER CHILDREN WHO LIVE WITH YOU. A child was awake between [insert value from CC2] and [insert value from CC4]. At which times or during which activities during that time period was/were [FNAME] [LNAME] in your care? (insert name(s) of non-own household children in age of 0-12 years old)

* Probe: Any other times or activities?

1. Activities where child was in your care [Go to next row]

96. All day.

97. None/no more childcare activities. [If first row of CC5B=97, or if no entries in CC5B, go to CC5B_CK]

Don't Know, Refused [If CC5B= Don't Know, Refused, go to next row]

[If no more rows or if CC5B=96, 97 and at least one non-own household child < 13, then go to CC6]
[Else if no more rows or if CC5=96, 97 and no own nonhousehold children, go to CC8]

CC5B_CK
Universe: If first row of CC5B=97 or no entries in CC5B

The interviewer may clarify with the respondent that:

*No activities were selected even though there are non-own children in the household <13. *Enter reason or use the arrow key to go back to correct

1. No secondary childcare activities
2. Respondent didn't know
3. Respondent refusal to answer
4. Child was away from home yesterday
5. Respondent was away from the home yesterday [If at least one own nonhousehold child < 13, then go to CC6] [Else if no own nonhousehold children, then go to CC8]

CC6

Universe: At least 1 OWN Nonhousehold child under 13

Now I'd like to ask you about YOUR children who don't live with you. During any part of the day yesterday, was/were [FNAME][LNAME] in your care? (insert name(s) of own, nonhousehold children under 13)

1. Yes [Go to CC7]
2. No
Don't Know, Refused [Go to CC8]

CC7
Universe: CC6 = 1

At which times or during which activities was/were [FNAME] [LNAME] in your care? (insert name(s) of own, nonhousehold children under 13)

1. Activities where nonhousehold child was in your care. [Go to next row]
97. None/no more nonhousehold childcare activities.
Don't Know, Refused [If 97 in first row or no "1" in column, then go to CC7_CK]
[Else If no more rows go to CC8]

CC7_CK
Universe: If first row of CC7=97 or no entries in CC7 The interviewer may clarify that:

*No activities were selected even though DP said he or she had a nonhousehold child.
*Enter reason or use the arrow key to go back to correct.

1. No secondary childcare activities.
2. DP didn't know.
3. DP refused to answer. [Go to CC8]

CC8

Universe: ALL

If household roster includes children under 13 years of age: Other than [FNAME] [LNAME] during any part of the day yesterday was a child who is 12 years old or younger in your care? Please do not include any paid childcare. (fills household and own nonhousehold children's names)

If household roster does not include children under 13 years of age: During any part of the day yesterday was a child who is 12 years old or younger in your care? Please do not include any paid childcare.

IF YES - Ask:
When was that?
1. Activities where other child was in your care. [Next line of grid] [If no more entries go to CC9] 97. None/no more childcare activities. [If 97 on first line of grid, go to V1]
Don't Know, Refused [Else if entries of 1 prior to entry of 97 go to CC9]

CC9
Universe: CC8 = 97 and/or entry of "1" in at least one column.

Is that child/are those children related to you?
1. Yes
2. No
3. Some are, some are not [Go to V1]

Volunteering

Three summary questions identify which activities reported in the diary were done as part of volunteering for or through an organization.

V1

Universe: All

Now I'd like to ask you about volunteer activities, that is, activities for which people are not paid, except perhaps expenses. Yesterday, did you do any volunteer activities for or through an organization?
1. Yes [Go to V3]
2. No [Go to V2]
Don't Know [Go to V2]
Refused [Go to ELDER_INTRO]

V2

Universe: V1 = 2, Don't Know

Sometimes people don't think of activities they do for schools, or youth, or religious organizations as volunteer activities. Yesterday, did you volunteer for these or similar organizations?
1. Yes [Go to V3]
2. No [Go to ELDER_INTRO]
Don't Know, Refused [Go to ELDER_INTRO]

V3

Universe: V1= 1 OR V2 =1

Which of the activities that you told me about were volunteer activities?

1. Volunteer activities. [Next line of grid] [If no more entries, go to ELDER_INTRO] 97.
None/no more volunteer activities. [Go to ELDER_INTRO]
Don't Know, Refused [Next line of grid] [If no more entries, go to ELDER_INTRO]

S5a: Eldercare (2011 and later) Universe: Summary Questions are complete

Following the summary questions, the interviewer asks a set of questions to identify whether or not the respondent is an eldercare provider. The ATUS defines an eldercare provider as someone who has provided unpaid care or assistance more than one time in the 3-4 months prior to the interview day to a person who needed help because of a condition related to aging. If the respondent is an eldercare provider, the interviewer collects information about the care recipient's age, relationship to the respondent, the duration of care, whether or not care was provided

yesterday, and if so, the activities done as care. Information about whether the eldercare recipient lives in the same household as the respondent also is captured.

The eldercare questions were introduced in 2011. Prior to 2011, questions about trips away from home were asked. The 2003-2010 ATUS questionnaire, which includes the trips questions, is available online at www.bls.gov/tus/tuquestionnaire0310.pdf.

ELDER_INTRO

Universe: All

The next set of questions are about times you may have recently spent assisting or caring for an adult who needed help because of a condition related to aging. For example, as people grow older, it sometimes becomes difficult for them to perform various activities without help – such as grooming, driving, managing the household, taking medication or other common activities. Care may be provided in your home, their home, or at a care facility.

* Read if necessary

A condition related to aging is an ongoing ailment or physical or emotional limitation that typically affects older people. Examples may include becoming more frail; having difficulty seeing, hearing, or physically moving; becoming more forgetful; tiring more quickly; or specific medical ailments that are more common among older adults. It also refers to existing conditions that become progressively worse as one ages.

1. Enter 1 to continue [Go to ELDER]

ELDER

Universe: ELDER_INTRO = 1

Not including financial assistance or help you provided as part of your paid job, since the 1st of [fill= reference month], have you provided any care or assistance for an adult who needed help because of a condition related to aging?

*Reference month is three months prior to the interview date.

. 1. Yes	[Go to ELFREQ]
. 2. No	
Don't Know, Refused	[If DP laid off, go to LAYDT]<br>[If DP didn't work but isn't prevented from work, go to LK]<br>[If DP is prevented from working and AGE ≠ 15-49, go to Module or go to S9: (Conclusion)]

ELFREQ

Universe: ELDER = 1

How often did you provide this care?

*If the respondent volunteers that he/she cares for multiple individuals, the respondent should answer this question based on the recipient for which he/she provides the most care.

*Items in bold read aloud.

1. **Daily** [Go to ELNUM]
2. **Several times a week** [Go to ELNUM]
3. **About once a week** [Go to ELNUM]
4. **Several times a month** [Go to ELNUM]
5. **Once a month** [Go to ELNUM]
6. One time [See below]
7. Other (specify) [Go to ELFREQ_SP]

Don't Know, Refused

<6, Don't Know, Refused> [If DP laid off, go to LAYDT]
[If DP didn't work but isn't prevented from work, go to LK]
[If DP is prevented from working and AGE ≠ 15-49, go to Module or go to S9: (Conclusion)]
[If DP is prevented from working and AGE = 15-49, Don't Know, or Refused, go to SCHENR]

ELFREQ_SP

Universe: ELFREQ = 7

*Specify

<250 characters>

[Go to ELNUM]

ELNUM

Universe: (ELFREQ = 1-5) OR (entry in ELFREQ_SP)

Since the 1st of [fill = reference month], how many people have you provided this care to?

*Reference month is three months prior to the interview date.

<1-5, Don’t Know, Refused> [Go to ELWHO]

The ELWHO, HHDUR, and HHYRS questions collect information about eldercare recipients who live in the same household as the respondent. Age and relationship information for household eldercare recipients is already known from the household roster (section 2), and thus this information is not collected again.

ELWHO
Universe: (ELNUM = 1-5, Don’t Know, or Refused)

Who did you give this care to? (*separate with commas)

[Fill = persons from household roster]

*Read if necessary: Does this person live in your household?

*Check all household members that apply OR press ENTER if person is not listed below to go to WHO_ELSE.

<2-32> [Go to HHDUR]
< Don’t Know, Refused, Blank> [Go to WHO_ELSE]

HHDUR

Universe: ELWHO = 2-32

How long have you provided care to [household member identified in ELWHO]?

1. 0 to 5 months
2. 6 to 11 months
3. 1 year
4. More than one year

<1-3, Don’t Know, Refused> [If ELNUM > 1 and there is at least one nonhousehold care recipient, then go to WHO_ELSE]
[Else go to ELCARE]
<4> [Go to HHYRS]

HHYRS
Universe: HHDUR = 4

How many years?

<1-96, Don't Know, Refused>

[If ELNUM > 1 and at least one nonhousehold care recipient, then go to WHO_ELSE]
[Else go to ELCARE]

The WHO_ELSE question is the first of several questions that collect information about eldercare recipients who do not live in the same household as the respondent. Relationship and age information is collected for non-household eldercare recipients because, unlike those care recipients living in the same household as the respondent, this information was not collected in the household roster (section 2).

WHO_ELSE
Universe: (ELNUM > number of persons selected in ELWHO) OR (ELWHO = Don't Know, Refused, Blank)

Who else/did you provide care to?

33. Mother
34. Father
35. Spouse

3637. . Partner Brother

38. Sister
39. Mother-in-law
40. Father-in-law
41. Aunt

4243. . Uncle Friend

44. Neighbor
45. Other (Valid option in 2011-12)
47. Grandmother/Great-grandmother*
48. Grandfather/Great-grandfather*

4956. . Other related person* Other non-relative (Specify)*

*Note: Response options 47-56 were added to the WHO_ELSE question in January 2013 to better match the relationship categories in the household roster.

ELWHO_SP

Universe: (WHO_ELSE = 45)

*Specify.

<250 characters> [Go to ELAGE]

ELAGE

Universe: (At least one response for WHO_ELSE = 33-49, 56, Don’t Know, or Refused) OR (entry in ELWHO_SP)

If ELNUM = 1 then: What was his/her age on (fill: reference month) 1 st?
Else: What was your (first person identified in WHO_ELSE)’s age on (fill: reference month) 1st?

*Reference month is three months prior to the interview date.

<1-120, Don’t Know, Refused> [Go to ELDUR]

ELDUR
Universe: (ELAGE = 1-120, DK, or R)

How long have you provided care to him/her?

1. 0 to 5 months
2. 6 to 11 months
3. 1 year
4. More than one year

<1-3, Don't Know, Refused> [If ELNUM > 1 and at least one nonhousehold care recipient's age has not been collected, go to ELAGE2]

[Else if ELNUM > 1 and at least one household care recipient's duration of care has not been collected, go to ELDUR2]
[Else go to ELCARE]

<4> [Go to ELYRS]

ELYRS
Universe: **ELDUR = 4**

How many years?

<1-99, Don't Know, Refused>
[If ELNUM > 1 and at least one nonhousehold care recipient's age has not been collected, then go to ELAGE2]
[Else if ELNUM > 1 and at least one household care recipient's duration of care has not been collected, go to ELDUR2]
[Else go to ELCARE]

ELAGEUniverse: 2 **[(ELDUR = 1-3, Don't Know, Refused) AND (ELNUM > 1 and at least one nonhousehold care recipient whose age has not been collected)] OR [(ELYRS = 1-99, DK, or R) AND (ELNUM > 1 and at least one nonhousehold care recipient whose age has not been** collected)]

What was your (second person identified in WHO_ELSE)'s age on (fill: reference month) 1 st?

*Reference month is three months prior to the interview date.

<1-120, DK, R> [Go to ELDUR2]

ELDUR2
Universe: [(ELDUR = 1-3, DK, or R) AND (at least one household care recipient whose duration of care has not been collected)] OR [(ELYRS = 1-99, DK, or R) AND (at least one household care recipient whose duration of care has not been collected)] OR (ELAGE2=1-120, DK, R)

How long have you provided care to him/her?

5. 0 to 5 months
6. 6 to 11 months

7.8. 1More than one year year

<1-3, Don't Know, Refused> [If ELNUM > 1 and at least one nonhousehold care recipient's age has not been collected, go to ELAGE3]
[Else if ELNUM > 1 and at least one household care recipient's duration of care [has not been collected, go to ELDUR3] Else go to ELCARE]

<4> [Go to ELYRS2]

ELUniverse: YRS2 ELDUR = 4

How many years?

<1-99, Don't Know, Refused> [If ELNUM > 1 and at least one nonhousehold care recipient's age has not been collected, then go to ELAGE3]
[Else if ELNUM > 1 and at least one household care recipient's duration of care has not been collected, go to ELDUR3]
[Else go to ELCARE]

NOTE: Interviewers continue to ask ELAGE(3-5), ELDUR(3-5), and ELYRS(3-5) until the age and duration of care information has been collected for each respondent identified in WHO_ELSE for a total of up to five care recipients (household and non-household combined).

ELCARE

Universe: **[(ELDUR = 1-3, Don't Know, or Refused) AND (ELNUM = 1)]**
OR [(ELDUR = 1-3, Don't Know, or Refused) AND (ELNUM > 1 and ages for all

nonhousehold care recipients have been collected and durations of care for all household and nonhousehold care recipients have been collected)]

OR [(ELYRS = 1-99, Don't Know, or Refused) AND (ELNUM = 1)] OR [[(ELYRS = 1-99, Don't Know, or Refused) AND (ELNUM > 1 and ages for all nonhousehold care recipients have been collected and durations of care for all household and nonhousehold care recipients have been collected)]

Did you provide any care or assistance yesterday?

1. Yes [Go to EC24]
2. No

Don't Know, Refused

.

EC24

Universe: (ELCARE = 1)

At which times or during which activities did you provide that care or assistance yesterday?

* Enter 1 for all relevant activities.
* Enter 97 for none / no more activities.
*Enter 96 for all day.
* Use the up/down arrow keys, to move to the correct row.

1. Activities done as eldercare.

97. None/no more eldercare activities.
Don't Know, Refused
[Next line of grid]

[If DP laid off, go to LAYDT]
[If DP didn't work but isn't prevented from work, go to LK] [If DP is prevented from working and AGE ≠ 15-49, go to Module or go to S9: (Conclusion)]
[If DP is prevented from working and AGE = 15-49, Don't
Know, or Refused, go to SCHENR]

S7 (a): Labor Force Status: Layoff/Looking

Universe: DP is laid off

Next the interviewer asks questions to determine the DP's labor force status. DPs who reported being unemployed, on layoff, or disabled in S3: (Employment) are asked if and how they are actively looking for work. Those who reported being on layoff are also asked if and when they expect to be recalled to their jobs. In the ATUS, these questions are worded exactly as in CPS, except that the CPS wording "last week" is replaced with "last seven days."

Note: Some skip patterns refer to "modules." A module is an additional set of questions about a specific topic. Modules are not asked in every year.

LAYDT

Universe: DP is laid off

Now I would like to ask a few more questions about the job you are on layoff from. Has your employer given you a date to return to work?
1. Yes [Go to LAYAVL]
2. No
Don't Know, Refused [Go to LAY6M]

LAY6M
Universe: LAYDT = 2, Don't Know, Refused

Have you been given any indication that you will be recalled to work within the next 6 months?
1. Yes [Go to LAYAVL] 2. No

Don't Know, Refused [Go to LK]

LAYAVL

Universe: LAYDT = 1 or LAY6M = 1

Could you have started a job in the LAST SEVEN DAYS, if one had been offered?
1. Yes [Go to LAYLK]
2. No [Go to LAYAVR]
Don't Know, Refused [Go to LAYLK]

LAYAVR
Universe: LAYAVL = 2

Why is that?
1. Own temporary illness
2. Going to School [Go to LAYLK]
3. Other [Enter verbatim response, go to LAYLK]

Don’t Know, Refused [Go to LAYLK]

LAYLK
Universe: LAY6M = 1 or LAYDT = 1

Even though you expect to be called back to work, have you been looking for work during the last 4 weeks?
1. Yes 2. No

Don’t Know, Refused [If age ≠ 15-49, Don’t Know, Refused go to Module OR go to S9: (Conclusion)]
[If DP is prevented from working and age = 15-49, go to SCHENR]

LK

Universe: LAY6M = 2, Don’t Know, Refused or DP didn’t work and didn’t have a disability that prevented DP from working or DP indicated retired but wanted a job full or part time

Now I would like to ask you a few questions about your job search activities. Have you been doing anything to find work during the last 4 weeks?
1. Yes [Go to LKM1]
2. No
3. Retired [If age ≠ 15-49, go to Module OR go to S9: Conclusion]
[Else if age = 15-49, Don’t Know, Refused, go to SCHENR]

4. Disabled
5. Unable [If DIS1a or DIS1b or DIS1c = blank and LK = 4, then go to DIS1d]
[If DIS2a or DIS2b or DIS2c and LK = 5, then go to DIS2d]

Don’t Know, Refused [If age ≠ 15-49, go to Module OR go to S9: (Conclusion)]
[Else if age = 15-49, Don’t Know, Refused go to SCHENR]

DIS1d
Universe: DIS1a or DIS2a or DIS1b or DIS2b or DIS1c or DIS2c = blank and LK = 4

Does your disability prevent you from accepting any kind of work during the next six months?
1. Yes 2. No

Don’t Know, Refused [If age ≠ 15-49, go to Module OR go to S9: (Conclusion)]
[Else if age = 15-49, Don’t Know, Refused go to SCHENR]

DIS2d
Universe: DIS1a or DIS2a or DIS1b or DIS2b or DIS1c or DIS2c = blank and LK = 5

Do you have a disability that prevents you from accepting any kind of work during the next six months?
1. Yes 2. No

Don't Know, Refused [If age ≠ 15-49, go to Module OR go to S9: (Conclusion)]
[Else if age = 15-49, Don't Know, Refused go to SCHENR]

For the variable LKM, the interviewer follows the pattern described below for up to six iterations, if necessary.

LKM1-LKM6
Universe for LKM1: LK = 1
Universe for LKM2-6: LKM1-LKM5 = 1-11, 13

What are all of the things you have done to find work during the last 4 weeks?
* Do not read answer categories

ACTIVE
1. Contacted employer directly/interview
2. Contacted public employment agency
3. Contacted private employment agency
4. Contacted friends or relatives
5. Contacted school/university/employment center
6. Sent out resumes/filled out applications
7. Check union/professional registers
8. Placed or answered ads [Go to LKM2] [Else if on LKM6, then go to LKAVL]
9. Other active [Enter verbatim response, then go to LKM2]

PASSIVE
10. Looked at ads [Go to LKM2]
11. Attended job training programs/courses [If LKM1 – LKM5 = 10, 11, 13, then go to LKPS1] [Else go to LKAVL]
12. Nothing [Go to LKDK1]
13. Other passive [Enter verbatim response, then go to LKM2]
14. Don't Know [Go to LKDK1]
15. Refused [If age ≠ 15-49, go to Module OR go to S9: (Conclusion)] [Else if age = 15-49, Don't Know, Refused go to SCHENR]

97. No additional answers [If LKM1 – LKM5 = 10, 11, 13, then go to LKPS1] [Else go to LKAVL]

For the variable LKDK, the interviewer follows the pattern described below for up to six iterations, if necessary.

LKDK1-LKDK6
Universe for LKDK1: LKM1 = Don't Know, 12
Universe for LKDK2-LKDK6: LKDK1-5= 1-11, 13

You said you have been trying to find work. How did you go about looking?
* Do not read answer categories

ACTIVE
1. Contacted employer directly/interview
2. Contacted public employment agency
3. Contacted private employment agency
4. Contacted friends or relatives
5. Contacted school/university/employment center
6. Sent out resumes/filled out applications
7. Check union/professional registers
8. Placed or answered ads [Go to LKDK2]
9. Other active [Enter verbatim response, then go to LKDK2]
[Else if on LKDK6, enter verbatim and then go to LAKVL]

PASSIVE
10. Looked at ads [Go to LKDK2] 11. Attended job training programs/courses [Go to LKDK2]
[If LKDK6 and if LKDK1 –LKDK5 = 10, 11, 13 then:
[If age ≠ 15-49, go to Module OR go to S9: (Conclusion)]
[Else if age = 15-49, Don't Know, Refused go to SCHENR]
[Else go to LKAVL]]

12. Nothing [If age ≠ 15-49, go to Module OR go to S9: (Conclusion)]
[Else if age = 15-49, Don't Know, Refused go to SCHENR]

13. Other passive [Enter verbatim response, then go to LKDK2]
[Else if LKDK6, enter verbatim and then go to LAKVL]

14. Don't Know, Refused [If age ≠ 15-49, go to Module OR go to S9: (Conclusion)]
[Else if age = 15-49, Don't Know, Refused go to SCHENR]

97. No additional answers [If LKDK6 and if LKDK1 –LKDK5 = 10, 11, 13 then:
[If age ≠ 15-49, go to Module OR go to S9: (Conclusion)]
[Else if age = 15-49, Don't Know, Refused go to SCHENR]
[Else go to LKAVL]]

For the variable LKPS, the interviewer follows the pattern described below for up to six iterations, if necessary.

LKPS1-LKPS6

**Universe for LKPS1:**	**The DP reported a passive job search activity, and/or refused to answer questions about job search activities.**

Universe for LKPS2-LKPS6: LKPS1-LKPS5=1-11, 13

Can you tell me more about what you did to search for work?

* Do not read answer categories

ACTIVE	
1. Contacted employer directly/interview	
2. Contacted public employment agency	
3. Contacted private employment agency	
4. Contacted friends or relatives	
5. Contacted school/ university/ employment center	
6. Sent out resumes/filled out applications	
7. Check union/ professional registers	
8. Placed or answered ads	[Go to LKPS2]<br>[Else if LKPS6, go to LKAVL]
9. Other active	[Enter verbatim, then go to LKPS2]
PASSIVE	
10. Looked at ads	[Go to LKPS2]
11. Attended job training programs/ courses	[If LKPS6 and if LKPS1 – LKPS5 = 10, 11, 13, then:<br>[If age ≠ 15-49, go to Module OR go to S9: (Conclusion)]<br>[Else if age = 15-49, Don't Know, Refused, go to SCHENR]<br>[Else go to LKAVL]]
12. Nothing	[If age ≠ 15-49, go to Module or go to S9: (Conclusion)]<br>[Else if age = 15-49, Don't Know, Refused, go to SCHENR]
13. Other passive	[Enter verbatim, then go to LKPS2]<br>[If on LKPS6, then enter verbatim and go to LKAVL]
14. Don't Know, Refused	[If age ≠ 15-49, go to Module or go to S9: (Conclusion)]<br>[Else if age = 15-49, Don't Know, Refused, go to SCHENR]
97. No additional answer	[If LKPS6 and if LKPS1 – LKPS5 = 10, 11, 13, then:<br>[If age ≠ 15-49, go to Module OR go to S9: (Conclusion)]<br>[Else if age = 15-49, Don't Know, Refused go to SCHENR]<br>[Else go to LKAVL]]

LKAVL

**Universe:**	**The DP reported an active job search activity.**

LAST WEEK, could you have started a job if one had been offered?

1. Yes [Go to next section]
2. No [Go to LKAVR]
Don't Know, Refused [If age ≠ 15-49, go to Module OR go to S9: (Conclusion)]
[Else if age = 15-49, Don't Know, Refused go to SCHENR]

LKAVR
Universe: LAYAVL = 2

Why is that?
1. Waiting for new job to begin
2. Own temporary illness
3. Going to school [If age ≠ 15-49, go to Module OR go to S9: (Conclusion)]
[Else if age = 15-49, go to SCHENR]
4. Other (Specify) [Enter verbatim, then:
[If age ≠ 15-49, go to Module OR go to S9: (Conclusion)]
[Else if age = 15-49, go to SCHENR]]

Don't Know, Refused [If age ≠ 15-49, go to Module OR go to S9: (Conclusion)]
[Else if age = 15-49, go to SCHENR]

S7 (b): Labor Force Status: Employed

Universe: DP is employed

Next, the interviewer updates information on the DP's job title and description, and the industry in which the DP works, using questions unchanged from the CPS. If the DP's job has changed since the final CPS interview, the job title, description, and industry are all updated. If the interviewer confirms that the DP's job title, description, and industry have *not* changed, then the ATUS continues to a section on Earnings and School Enrollment.

IO_LEAD

Universe: DP worked

Now I have a few questions about [your job/your main job/the job from which you are absent.]

1. Enter 1 to continue. [If DP is employed in ATUS, but MLR=3-7 in CPS, go to IO1INT]
   [Else if DP is employed in ATUS and CPS, but employer name or industry were missing, go to IO1INT]
   [Else if DP employed in both ATUS and CPS, and employer name was not blank, go to IODP1]

IODP1

Universe: DP is employed and employer name was not blank

Last time we talked to someone in this household, that would be [month of final CPS interview] it was reported that you worked for [Employer name]. Do you still work for [Employer name] [at your main job]?

1. Yes [If CPS industry code was previously missing, go to IO1IND]
   [If CPS occupation code was previously missing, go to IO1OCC]
   [Else go to IODP2]
2. No [Go to IO1INT]

Don't Know, Refused [Go to IO1INT]

IODP2

Universe: IODP1 = 1

Have the usual activities and duties of your job changed since [month of final CPS interview]?

1. Yes [Go to IO1OCC]
2. No

Don't Know, Refused [If CPS occupation code was previously missing, go to IO1OCC]
[If CPS usual job activities were previously missing, go to IO1OCC]
[If CPS weekly earnings were allocated, go to S8: (Earnings and School Enrollment)]
[Else go to IODP3]

IODP3
Universe: **(IODP2 = 2, Don't Know, or Refused) AND**
(Industry and Occupation codes entered in CPS)

AND (Usual job activities entered in CPS)

Last time we talked to someone in this household, you were reported as (a/an) [CPS occupation] and your usual activities were [CPS usual job activities]. Is this an accurate description of your current job?
1. Yes [Go to S8: (Earnings and School Enrollment)]
2. No [Go to IO1OCC]
Don't Know, Refused [Go to S8: (Earnings and School Enrollment)]

IO1INT
Universe: **In ATUS DP works AND [(MLR=3-7 in CPS)**
OR (Employer name missing in CPS)]

OR IODP1 = 2, Don't Know, Refused

Are you employed by government, by a private company, a non-profit organization or are you self-employed [or working in a family business]?
1. Government [Go to IO1GVT]
2. Private for-profit company
3. Non-profit organization [Go to IO1NMP]
4. Self-employed
5. Working in family business [Go to IO1INC]
Don't Know, Refused [Go to IO1NMP]

IO1GVT

Universe: IO1INT = 1

Would that be the federal, state or local government?
1. Federal
2. State
3. Local (county, city, township)
Don't Know, Refused [Go to IO1NMG]

IO1INC
Universe: **IO1INT = 4, 5**

Is this business incorporated?
1. Yes [Go to IO1NMB]
2. No
Don't Know, Refused [Go to IO1NMB]
[Else if [BUS = 1 AND ((BUS2 ≠ 1)
OR (there is only one household member))], go to IO1WP]

IO1WP

Universe: IO1INC = 2, Don't Know, Refused AND [BUS =1 AND ((BUS2 ≠1) OR (only one household member))]

Were you working for pay?

1. Yes 2. No

Don't Know, Refused [Go to IO1NMB]

IO1NMP

Universe: IO1INT = 2, 3, Don't Know, Refused

What is the name of the [company/non profit organization] for which you work [at your main job]?

1. Enter name [Go to IO1IND]

IO1NMG

Universe: IO1GVT = 1-3, Don't Know, Refused

What is the name of the government agency for which you work [at your main job]?

1. Enter name [Go to IO1IND]

IO1NMB

Universe: IO1INC = 1, 2, Don't Know, Refused

What is the name of your business? What is the name of the business for which you work?

1. Enter name [Go to IO1IND]

IO1IND

Universe: (IODP1 = 1 AND CPS industry code was previously missing)

OR IO1NMP, IO1NMG, or IO1NMB = valid response

What kind of business or industry is this?

* Read if necessary: What do they make or do where you work?

1. Enter [Go to IO1MFG]

Don't Know, Refused [Go to IO1OCC]

IO1MFG
Universe: IO1IND ≠ Don't Know, Refused

Is this business or organization mainly manufacturing, retail trade, wholesale trade, or something else?
1. Manufacturing
2. Retail trade
3. Wholesale trade
4. Something else
   Don't Know, Refused [Go to IO1OCC]

IO1OCC
Universe: Industry and Occupation codes missing in CPS AND [(DP's usual job duties have changed since CPS) OR (DP's usual job activities have not changed)]
OR Occupation, Industry and job activities not correct in CPS
OR IO1IND = Don't Know, Refused
OR IO1MFG = 1-4, Don't Know, Refused

What kind of work do you do, that is what is your occupation?
(For example: plumber, typist, farmer)
1. Enter verbatim response
   Don't Know, Refused [Go to IO1DT1]

IO1DT1
Universe: IO1OCC = Verbatim response, Don't Know, Refused

What are your usual activities or duties at this job?
(For example: types, keeps account books, files, sells cars, operates printing press, lays bricks)
1. Enter verbatim response
   Don't Know, Refused [Go to S8: (Earnings and School Enrollment)]

S8: Earnings and School Enrollment

Universe: All

For those who changed jobs or employers since the final CPS interview, or whose weekly earnings were imputed in the CPS, the interviewer collects data on the DP's earnings, including any overtime. Based on responses to several questions about earnings, the instrument calculates the DP's usual weekly earnings, which is confirmed by the DP.

Finally, all DP's between ages 15 and 49 are asked about school enrollment status.

Note: Some skip patterns refer to "modules." A module is an additional set of questions about a specific topic. Modules are not asked in every year.

ERNP
Universe: **DP changed jobs or employers since CPS**
OR DP was not employed in CPS, but employed in ATUS
OR DP's weekly earnings were allocated in CPS

This month I have a few questions about earnings.

1. Enter 1 to continue. [Go to ERNPR]

ERNPR
Universe: **All**

For your [main] job, what is the easiest way for you to report your total earnings BEFORE taxes or other deductions: hourly, weekly, annually, or on some other basis?
* If necessary: We use this information to compare the amount that people earn in different types of jobs.
1. Hourly
2. Weekly
3. Bi-weekly
4. Twice monthly
5. Monthly
6. Annually [Go to ERNUOT]
7. Other (specify) [Enter verbatim response, then go to ERNUOT]
Don't Know [Go to ERNUOT]
Refused [If age is 15 – 49, Don't Know, Refused, go to SCHENR]
[Else go to Module OR go to S9: (Conclusion)]

ERNUOT

Universe: **ERNPR = 1-7, Don't Know or verbatim response entered.**

Do you usually receive overtime pay, tips, or commissions [at your main job]?
1. Yes
2. No
Don't Know, Refused [If ERNPR = 1, go to ERNHRT]
[Else if ERNPR = 2-7, or Don't Know then go to ERNWK2]

ERNHRT
Universe: **ERNPR = 1**

If ERNUOT =1: EXCLUDING overtime pay, tips and commissions what is your hourly rate of pay on [this/your main] job?

IF ERNUOT ≠ 1: What is your hourly rate of pay on [this/ your main] job?
1. Enter verbatim response [Go to ERNHR] Don't Know [Go to ERNHRE]
Refused [If age 15-49, Don't Know, Refused, go to SCHENR]
[Else if age ≠ 15-49 go to Module OR go to S9: (Conclusion)]

ERNHRE

Universe: ERNHRT = Don't Know

What is your best estimate of your hourly rate of pay?
1. Enter verbatim response [Go to ERNHR]
Don't Know, Refused [If age = 15-49, Don't Know, Refused go to SCHENR]
[Else if age ≠ 15-49 go to Module OR go to S9: (Conclusion)]

ERNHR

Universe: ERNHRT = verbatim response OR ERNHRE = verbatim response

How many hours do you usually work per week at this rate?
1. Enter response [If ERNUOT = 1, go to ERNOTP] [Else go to ERNVR1]
Don't Know, Refused [If HRUSL1 = Don't Know, Hours Vary, Refused, go to ERNWK2]
[Else if ERNUOT = 1 AND HRUSL1=1-99, go to ERNOTP]
[Else go to ERNVR1]

ERNOTP
Universe: ERNUOT = 1 AND (HRUSL1 = 1 – 99 and ERNHR = 1-99, Don't Know, Refused)

[At your main job] How much do you usually receive JUST in overtime pay, tips or commissions, before taxes or other deductions?
*Enter periodicity first.
1. Per hour
2. Per day
3. Per week 4. Per month
5. Per year [Go to ERNOTA]
6. Other
Don't Know [Go to ERNOTE]
Refused [If age = 15 – 49, Don't Know, Refused, go to SCHENR]
[Else if age ≠ 15-49 go to Module OR go to S9: (Conclusion)]

ERNOTA

Universe: ERNOTP = 1 - 5

Enter Dollar Amount
1. Enter response [If ERNOPT = 1, go to ERNOH] [If ERNOTP = 2, go to ERNOTE]
[If ERNOTP =3, 4, 5, go to ERNVR1]

ERNOTE
Universe: ERNOTP = 6, Don't Know OR (ERNOTA = verbatim response and ERNOTP = 2)

What is your best estimate of how much you usually earn weekly, JUST in overtime pay, tips, or commissions, before taxes or other deductions?
1. Enter verbatim response [Go to ERNVR1]
Don't Know, Refused [IF age = 15 – 49, Don't Know, Refused, go to SCHENR]
[Else if age ≠ 15-49 go to Module OR go to S9: (Conclusion)]

ERNOH

Universe: ERNOTP = 1 AND ERNOTA = verbatim response

How many hours do you usually work per week at this rate?
1. Enter verbatim response [Go to ERNVR1]
Don't Know [Go to ERNOHE]
Refused [If age =15-49, Don't Know, Refused, Go to SCHENR]
[Else if age ≠ 15-49 go to Module OR go to S9: (Conclusion)]

ERNOHE

Universe: ERNOH = Don't Know

What is your best estimate of the number of hours per week you usually work at this rate?
1. Enter response [Go to ERNVR1]
Don't Know, Refused [IF age = 15 – 49, Don't Know, Refused go to SCHENR] [Else if age ≠ 15-49 go to Module OR go to S9: (Conclusion)]

ERNVR1
Universe: DP reported rate of pay, and timing of pay and does not earn overtime, tips or wages OR DP earns overtime, tips or wages, and reported amount of pay, and timing of pay

I have estimated your usual WEEKLY earnings [for your main job] as [HWKRN] before taxes or other deductions. Does that sound correct?
1. Yes [If age = 15 – 49, Don't Know, Refused go to SCHENR]
[Else if age ≠ 15-49 go to Module OR go to S9: (Conclusion)]

2. No
Don't Know [Verify information and correct error]
[If age = 15 – 49, Don't Know, Refused, go to SCHENR]
[Else if age ≠ 15-49 go to Module OR go to S9: (Conclusion)]

Refused [If age = 15 – 49, Don't Know, Refused, go to SCHENR]
[Else if age ≠ 15-49 go to Module OR go to S9: (Conclusion)]

If the DP responds that the calculated earnings are incorrect in ERNVR1, the ATUS instrument then goes through a series of variables to identify and correct the problem, then re-verifies the DP's earning information. This process is repeated until the earnings are correctly reported.

SCHENR
Universe: Age = 15 – 49, Refused but > 15, Don't Know, Refused

I just have a few more questions.
Are you enrolled in a high school, college, or university?
* Enter "1" if currently on holiday or school break.
* Enter "2" for summer vacation from school.

1. Yes [Go to SCHLVL]
2. No

Don't Know, Refused [Go to Module OR go to S9: (Conclusion)]

SCHLVL

Universe: SCHENR = 1

Would that be high school, college, or university?

1. High school
2. College or university

Don't Know, Refused [Go to SCHFT]

SCHFT

Universe: SCHLVL = 1, 2, Don't Know, Refused

Are you enrolled in school as a full-time or part-time student?

1. Full-time
2. Part-time [Go to Module OR go to S9: (Conclusion)]

S9: Conclusion

This section of the interview is used to close out the case.

THANKYOU

Universe: All households

Thank you for your time.

After the interview is complete, the interviewer answers two data quality questions.

INTDQUAL

Universe: All completed interviews

Is there any reason the information from this interview should NOT be used?

1. Yes [Go to DQUAL2]
2. No

DQUAL2

Universe: INTDQUAL = 1

Why do you think the data should not be used?

1. I feel the respondent was intentionally providing wrong answers
2. I feel the respondent was trying to provide correct answers, but could not correctly remember his/ her activities on the previous day
3. I feel the respondent deliberately reported very long duration activities
4. Other

Appendix B: Data Dictionary for Well-being Module of the 2013 ATUS

This appendix provides the documentation provided the ATUS detailing the set of questions and metadata used in the well-being module in the 2013 survey.

American Time Use Survey (ATUS) Data Dictionary:

2010, 2012, and 2013 Well-being Module Data

Variables collected in the ATUS Well-being Module

July 2014

Introduction

The National Institute on Aging sponsored the Well-being (WB) Module of the American Time Use Survey (ATUS). The ATUS is sponsored by the Bureau of Labor Statistics and conducted by the U.S. Census Bureau. The purpose of this document is to provide general information about the ATUS and WB Module and detailed information about the variables available on the ATUS WB Module data files: the WB Respondent files and the WB Activity files. The WB Module data files are available for 2010, 2012, and 2013 and contain information gathered from the ATUS interviews. In addition to the single-year WB Module data files, multi-year (combining 2010, 2012, and 2013) data files also are available. All WB Module questions were asked at the end of the ATUS interview.

In the ATUS, sample cases for the survey are selected monthly, and interviews are conducted continuously throughout the year. ATUS sample households are chosen from the households that completed their eighth (final) interview for the Current Population Survey (CPS). ATUS sample households are selected to ensure that estimates will be nationally representative of the U.S. civilian noninstitutional population age 15 and over. One individual is randomly chosen from each household and is interviewed by telephone about his or her activities on the day before the interview. We refer to the list of activities as the “time diary” and the day the diary refers to as the “diary day.” For more information about the ATUS, please see the ATUS User’s guide (www.bls.gov/tus/atususersguide.pdf).

All ATUS respondents were selected for the WB Module. In the module, 3 activities from the diary were randomly selected[1] and 7 questions related to the quality of life were asked about each activity; a few general questions about health status were asked for each respondent. Starting in 2012, two questions were added about general life satisfaction and how the respondent’s feelings compared to those on a typical day for which the respondent was being interviewed. The activities selected for the WB Module were required to meet the following criteria:

- The activity had to be at least 5 minutes in duration
- The following activities and responses were not eligible for selection: ○ Sleeping (0101xx) ○ Grooming (0102xx) ○ Personal Activities (0104xx) ○ Don’t know/Can’t remember (500106) ○ Refusal/None of your business (500105)

For more information about the questions asked in the WB Module and about the ATUS activity classification system, please see the WB Module questionnaire

[1] This description refers to the design of the WBM, however, there was an error in the software used to collect these data that affected the randomization process. For more information, see Appendix C of this document.”

(www.bls.gov/tus/wbmquestionnaire.pdf) and the ATUS Activity Coding Lexicons (www.bls.gov/tus/lexicons.htm).

This data dictionary lists all the variables available on the single-year and multi-year WB files and their valid values. It also provides directions on how to read the data dictionary.

Two other data dictionaries describe the basic ATUS data files. The first describes the ATUS-CPS file, which contains data from the CPS files for those selected to be surveyed for ATUS and members of their households. (The information on the ATUS-CPS file was collected two to five months before the ATUS interview and may have been out of date at the time of the ATUS survey.) The second is the ATUS interview data dictionary which describes the variables available on five files: the Roster file, the Activity file, the Who file, the Eldercare Roster file (introduced in 2011), and the Respondent file. These variables were collected and assigned in the ATUS interview.

Two other data dictionaries describe additional ATUS data files. The first describes the ATUS Survey Methods data, made up of the Case History file and the Call History file. The second describes the Trips file.

The trips questions were replaced by questions about eldercare in 2011; 2010 is the last year for which the Trips data dictionary was created.

Each of these additional data dictionaries describes variables from an individual year. They are available on the ATUS Web site at www.bls.gov/tus/dictionaries.htm.

Allocation Flags

For every edited variable (or all "E" variables), there is a corresponding allocation flag whose second character is "X." All remaining characters of the two variables' names are the same. For example, WXGENHTH is the allocation flag for WEGENHTH.

All allocation flags have the following list of possible values:

0 Value – no change
1 Blank – no change
2 Don't know – no change
3 Refused – no change
10 Value to value
11 Blank to value
12 Don't know to value

13 Refused to value
20 Value to longitudinal value
21 Blank to longitudinal value
22 Don't know to longitudinal value
23 Refused to longitudinal value
30 Value to allocated longitudinal value (unused)
31 Blank to allocated longitudinal value (unused)
32 Don't know to allocated longitudinal value (unused)
33 Refused to allocated longitudinal value (unused)
40 Value to allocated value
41 Blank to allocated value
42 Don't know to allocated value
43 Refused to allocated value
50 Value to blank

52 Don't know to blank
53 Refused to blank

Each digit of these valid values identifies how and why edited variables were allocated.

The first digit indicates how the allocation was made to the "E" (or edited) variable.

**First Digit**	
0 or Blank	No change between "U" variable and "E" variable
1	"E" variable changed to a value
2	"E" variable changed to a longitudinal value (the corresponding value from the CPS data)
3	"E" variable changed to an allocated longitudinal value (the corresponding allocated value from CPS data) - unused
4	"E" variable changed to allocated value
5	"E" variable changed to a blank

The second digit indicates why the "U" variable was allocated, whether the value was an unacceptable one, missing, don't know, or refused.

**Second Digit**	
0	"U" variable was equal to some value
1	"U" variable was blank (or -1)
2	"U" variable was don't know (or -2)
3	"U" variable was refused (or -3)

Valid Values

Each variable has a number of valid values or a range of valid values. For example, the variable WEGENHTH (general health) has five valid values: 1 for excellent, 2 for very good, 3 for good, 4 for fair, and 5 for poor. The variable WRTELIG (total time spent in all eligible activities), on the other hand, has a range of valid values – any entry between 5 and 1440 is considered valid. Individual valid values or a range of valid values are listed under each variable in the data dictionary.

Many ATUS variables have the following possible valid values:

Value	Description
-1	Blank
-2	Don't know
-3	Refused

Since so many variables have these possible values, they are not shown as valid entries for each variable. TUCASEID, the primary identification number for ATUS, does not have either a list of valid values or a range of valid values.

Name	Description	File
TRWBELIG	Flag identifying activities eligible for the Well-being Module	Activity File

Edited Universe: All activities

Valid Entries: 0 Activity not eligible for selection in the Well-being Module
1 Activity eligible for selection in the Well-being Module

* Note: Activities with codes of 0101xx, 0102xx, 0104xx, 500105, or 500106 or with durations less than 5 minutes are not eligible

Name	Description	File
TRWBMODR	Well-being Module respondent	Respondent File

Edited Universe: All respondents

Valid Entries: 0 Did not respond to Well-being Module
1 Responded to the Well-being Module

* Note: The Well-being Module was conducted in 2010, 2012 and 2013. All individuals on the Respondent file were selected to be interviewed for the Well-being Module.

Name	Description	File
TUACTIVITY_N	Activity line number	Activity File, Who File, WB Activity File

Valid Entries: 1 Min Value
91 Max Value

Name	Description	File
TUCASEID	ATUS Case ID (14-digit identifier)	All Files
TULINENO	ATUS person line number	ATUS-CPS File, Respondent File, Roster File, Who File, WB Respondent File, EC Roster File

Valid Entries: 1 Min Value
30 Max Value

* Note: The person selected to be interviewed for ATUS is always TULINENO = 1

Name	Description	File
WECANTRIL	Edited: Please imagine a ladder with steps numbered from 0 at the bottom to 10 at the top. The top of the ladder represents the best possible life for you and the bottom of the ladder represents the worst possible life for you. If the top step is 10 and the bottom step is 0, on which step of the ladder do you feel you personally stand at the present time?	WB Respondent File

Edited Universe: All respondents

Valid Entries: 0 Min Value
10 Max Value

* Note: This question was introduced in the 2012 WB Module. Therefore WECANTRIL is not on the 2010 Annual WB Respondent file. On the pooled WB Respondent file, WECANTRIL will have missing values for cases with TUYEAR=2010.

Name	Description	File
WEGENHTH	Edited: Would you say your health in general is excellent, very good, good, fair, or poor?	WB Respondent File

Edited Universe: All respondents

Valid Entries: 1 Excellent
2 Very Good

	3	Good
	4	Fair
	5	Poor

WEHBP	Edited: In the last five years, were you ever told by a doctor or other health professional that you have hypertension, also called high blood pressure, or borderline hypertension?	WB Respondent File

Edited Universe: All respondents

Valid Entries:

1	Yes
2	No

WEPAINMD	Edited: Did you take any pain medication yesterday, such as Aspirin, Ibuprofen or prescription pain medication?	WB Respondent File

Edited Universe: All respondents

Valid Entries:

1	Yes
2	No

WEREST	Edited: When you woke up yesterday, how well-rested did you feel? Did you feel very rested, somewhat rested, a little rested, or not at all rested?	WB Respondent File

Edited Universe: All responde ts

Valid Entries:

1	Very
2	Somewhat
3	A little
4	Not at all

WETYPICAL	Edited: Thinking about yesterday as a whole, how would you say your feelings, both good and bad, compared to a typical [FILL= DAY]? Were they better than a typical [FILL=DAY], the same as a typical [FILL=DAY], or worse than a typical [FILL=DAY]?	WB Respondent File

Edited Universe: All respondents

Valid Entries:

1	Better
2	The same
3	Worse

* Note: This question was introduced in the 2012 WB Module. Therefore WETYPICAL is not on the 2010 Annual WB Respondent file. On the pooled WB Respondent file, WETYPICAL will have missing values for cases with TUYEAR=2010.

The FILL is the day of the week about which the respondent was being interviewed. For example, if the diary day was a Tuesday, the respondent was asked how his feelings yesterday compared to a typical Tuesday.

WRTELIG	Total time spent in all activities eligible to be selected for Well-being	WB Respondent File module

Edited Universe: All Well-being respondents

Valid Entries:

5	Min Value
1440	Max Value

* Note: Includes time spent in all activities with duration 5 minutes or more, except 0101xx, 0102xx, 0104xx, 500105, and 500106

WUFINLWGT	Well-being module respondent weight	WB Respondent File

Valid Entries: 0 Min Value

9999999999 Max Value

WUFNACTWT	Well-being module original activity weight	WB Activity File

Valid Entries: 0 Min Value

99999999999 Max Value

* Note: For each activity, if the data were missing, or the respondent refused to answer or reported "don't know" to 4 or more of the affect questions (WUHAPPY, WUPAIN, WUSAD, WUSTRESS, and WUTIRED), meaningful (WUMEANING), or interaction (WUINTERACT) question, a value of 0 was assigned to the activity weight for that activity.

This weight was computed prior to implementing an adjustment to the WB activity weights. See note to WUFNACTWTC and WUFNACTWTP.

WUFNACTWTC	Well-being module adjusted annual activity weight	WB Activity File

Valid Entries: 0 Min Value

9999999999999999999 Max Value

* Note: For each activity, if the data were missing, or the respondent refused to answer or reported "don't know" to 4 or more of the affect questions (WUHAPPY, WUPAIN, WUSAD, WUSTRESS, and WUTIRED), meaningful (WUMEANING), or interaction (WUINTERACT) question, a value of 0 was assigned to the activity weight for that activity.

This weight includes an adjustment factor to compensate for an under sampling of late night activities. It should be used for annual estimates. See Appendix C for a more detailed discussion of these weights.

WUFNACTWTP	Well-being module adjusted pooled activity weight	WB Activity File

Valid Entries: 0 Min Value

999999999999 Max Value

* Note: For each activity, if the data were missing, or the respondent refused to answer or reported "don't know" to 4 or more of the affect questions (WUHAPPY, WUPAIN, WUSAD, WUSTRESS, and WUTIRED), meaningful (WUMEANING), or interaction (WUINTERACT) question, a value of 0 was assigned to the activity weight for that activity.

This weight includes a 3-year (2010, 2012, 2013) adjustment factor to compensate for an under sampling of late night activities. WUFNACTWTP is only available on the pooled Well-being activity data file. It should be used only for pooled estimates. See Appendix C for a more detailed discussion of these weights.

WUHAPORD	Order of WUHAPPY	WB Activity File

Valid Entries: 1 Min Value

5 Max Value

* Note: For each respondent to the Well-being module, the order of the affect questions (WUHAPPY, WUPAIN, WUSAD, WUSTRESS, and WUTIRED) was assigned randomly.
For each WB respondent, the order of the affect questions was the same for each selected activity.

WUHAPPY	From 0 to 6, where a 0 means you were not happy at all and a 6 means WB Activity File you were very happy, how happy did you feel during this time?	

Valid Entries: 0 Min Value

6 Max Value

WUINTERACT	Were you interacting with anyone during this time, including over the phone?	WB Activity File

Valid Entries: 1 Yes
2 No

WUMEANING	From 0 to 6, how meaningful did you consider what you were doing? 0 means it was not meaningful at all to you and a 6 means it was very meaningful to you.	WB Activity File

Valid Entries: 0 Min Value

6 Max Value

WUPAIN	From 0 to 6, where a 0 means you did not feel any pain at all and a 6 means you were in severe pain, how much pain did you feel during this time if any?	WB Activity File

Valid Entries: 0 Min Value

6 Max Value

WUPNORD	Order of WUPAIN	WB Activity File

Valid Entries: 1 Min Value
5 Max Value

* Note: For each respondent to the Well-being module, the order of the affect questions (WUHAPPY, WUPAIN, WUSAD, WUSTRESS, and WUTIRED) was assigned randomly.

WUSTRORD	Order of WUSTRESS	WB Activity File

Valid Entries: 1 Min Value
5 Max Value

* Note: For each respondent to the Well-being module, the order of the affect questions (WUHAPPY, WUPAIN, WUSAD, WUSTRESS, and WUTIRED) was assigned randomly.
For each WB respondent, the order of the affect questions was the same for each selected activity.

WUTIRED	From 0 to 6, where a 0 means you were not tired at all and a 6 means you were very tired, how tired did you feel during this time?	WB Activity File

Valid Entries: 0 Min Value
6 Max Value

WUTRDORD	Order of WUTIRED	WB Activity File

Valid Entries: 1 Min Value
5 Max Value

* Note: For each respondent to the Well-being module, the order of the affect questions (WUHAPPY, WUPAIN, WUSAD, WUSTRESS, and WUTIRED) was assigned randomly.
For each WB respondent, the order of the affect questions was the same for each selected activity.

WXCANTRIL	WECANTRIL: allocation flag	WB Respondent File

* Note: See introduction for allocation flag values.

WECANTRIL was introduced in the 2012 WB Module. Therefore WXCANTRIL is not on the 2010 WB Respondent file.

WXGENHTH	WEGENHTH: allocation flag	WB Respondent File

For each WB respondent, the order of the affect questions was the same for each selected activity.

WUSAD	From 0 to 6, where a 0 means you were not sad at all and a 6 means you WB Activity File were very sad, how sad did you feel during this time?	
	Valid Entries: 0	Min Value
	6	Max Value

WUSADORD	Order of WUSAD	WB Activity File
	Valid Entries: 1	Min Value
	5	Max Value

* Note: For each respondent to the Well-being module, the order of the affect questions (WUHAPPY, WUPAIN, WUSAD, WUSTRESS, and WUTIRED) was assigned randomly.
For each WB respondent, the order of the affect questions was the same for each selected activity.

WUSTRESS	From 0 to 6, where a 0 means you were not stressed at all and a 6 means WB Activity File you were very stressed, how stressed did you feel during this time?	
	Valid Entries: 0	Min Value
	6	Max Value

* Note: See introduction for allocation flag values

WXHBP	WEHBP: allocation flag	WB Respondent File

* Note: See introduction for allocation flag values

WXPAINMD	WEPAINMD: allocation flag	WB Respondent File

* Note: See introduction for allocation flag values

WXREST	WEREST: allocation flag	WB Respondent File

* Note: See introduction for allocation flag values

WXTYPICAL	WETYPICAL: allocation flag	WB Respondent File

* Note: See introduction for allocation flag values.
WETYPICAL was introduced in the 2012 WB Module. Therefore WXTYPICAL is not on the 2010 WB Respondent file.

Appendix C: Data Dictionary for Well-being Module of the 2021 ATUS

This appendix provides the documentation provided the ATUS detailing the set of questions and metadata used in the well-being module in the 2021 survey.

American Time Use Survey (ATUS) Data Dictionary:

2021 Well-being Module Data

Variables collected in the ATUS Well-being Module

June 2022

Introduction

The University of Minnesota and the University of Maryland sponsored the 2021 Well-being (WB) Module of the American Time Use Survey (ATUS). The ATUS is sponsored by the Bureau of Labor Statistics and conducted by the U.S. Census Bureau. The purpose of this document is to provide general information about the ATUS and WB Module and detailed information about the variables available on the 2021 ATUS WB Module data files: the WB Respondent files and the WB Activity files. The 2021 WB Module was conducted from March through December and data files contain information gathered from the ATUS interviews. All WB Module questions were asked at the end of the ATUS interview.

In the ATUS, sample cases for the survey are selected monthly, and interviews are conducted continuously throughout the year. ATUS sample households are chosen from the households that completed their eighth (final) interview for the Current Population Survey (CPS). ATUS sample households are selected to ensure that estimates will be nationally representative of the U.S. civilian noninstitutional population age 15 and over. One individual is randomly chosen from each household and is interviewed by telephone about his or her activities on the day before the interview. We refer to the list of activities as the "time diary" and the day the diary refers to as the "diary day." For more information about the ATUS, please see the ATUS User's guide (www.bls.gov/tus/atususersguide.pdf).

All ATUS respondents with diaries from March 1 through December 31, 2021 were selected for the WB Module. In the module, 3 activities from the diary were randomly selected and 7 questions related to the quality of life were asked about each activity; a few general questions about health status were asked for each respondent. The 2021 WB Module includes two questions about general

life satisfaction and how the respondent's feelings compared to those on a typical day for which the respondent was being interviewed. The activities selected for the WB Module were required to meet the following criteria:

- The activity had to be at least 5 minutes in duration
- The following activities and responses were not eligible for selection: ○ Sleeping (0101xx) ○ Grooming (0102xx) ○ Personal Activities (0104xx) ○ Don't know/Can't remember (500106) ○ Refusal/None of your business (500105)

For more information about the questions asked in the WB Module and about the ATUS activity classification system, please see the WB Module questionnaire (www.bls.gov/tus/wbmquestionnaire.pdf) and the ATUS Activity Coding Lexicons (www.bls.gov/tus/lexicons.htm).

This data dictionary lists all the variables available on the WB files and their valid values. It also provides directions on how to read the data dictionary.

Two other data dictionaries describe the basic ATUS data files. The first describes the ATUS-CPS file, which contains data from the CPS files for those selected to be surveyed for ATUS and members of their households. (The information on the ATUS-CPS file was collected two to five months before the ATUS interview and may have been out of date at the time of the ATUS survey.) The second is the ATUS interview data dictionary which describes the variables available on five files: the Roster file, the Activity file, the Who file, the Eldercare Roster file, and the Respondent file. These variables were collected and assigned in the ATUS interview.

Two other data dictionaries describe additional ATUS data files. The first describes the ATUS Survey Methods data, made up of the Case History file and the Call History file. The second describes the Trips file. The trips questions were replaced by questions about eldercare in 2011; 2010 is the last year for which the Trips data dictionary was created.

Each of these additional data dictionaries describes variables from an individual year. They are available on the ATUS Web site at www.bls.gov/tus/dictionaries.htm.

Allocation Flags

For every edited variable (or all "E" variables), there is a corresponding allocation flag whose second character is "X." All remaining characters of the two variables' names are the same. For example, WXGENHTH is the allocation flag for WEGENHTH.

All allocation flags have the following list of possible values:

0 Value – no change
1 Blank – no change
2 Don't know – no change
3 Refused – no change
10 Value to value
11 Blank to value
12 Don't know to value
13 Refused to value
20 Value to longitudinal value
21 Blank to longitudinal value
22 Don't know to longitudinal value
23 Refused to longitudinal value
30 Value to allocated longitudinal value (unused)
31 Blank to allocated longitudinal value (unused)
32 Don't know to allocated longitudinal value (unused)
33 Refused to allocated longitudinal value (unused)
40 Value to allocated value
41 Blank to allocated value
42 Don't know to allocated value
43 Refused to allocated value
50 Value to blank

52 Don't know to blank
53 Refused to blank

Each digit of these valid values identifies how and why edited variables were allocated.

The first digit indicates how the allocation was made to the "E" (or edited) variable.

**First Digit**	
0 or Blank	No change between "U" variable and "E" variable
1	"E" variable changed to a value
2	"E" variable changed to a longitudinal value (the corresponding value from the CPS data)
3	"E" variable changed to an allocated longitudinal value (the corresponding allocated value from CPS data) - unused
4	"E" variable changed to allocated value
5	"E" variable changed to a blank

The second digit indicates why the "U" variable was allocated, whether the value was an unacceptable one, missing, don't know, or refused.

Second Digit	
0	"U" variable was equal to some value
1	"U" variable was blank (or -1)
2	"U" variable was don't know (or -2)
3	"U" variable was refused (or -3)

TRWBELIG	Flag identifying activities eligible for the Well-being Module		Activity File
	**Edited Universe:**	All activities	
	**Valid Entries:**	0	Activity not eligible for selection in the Well-being Module
		1	Activity eligible for selection in the Well-being Module
	***Note**	Activities with codes of 0101xx, 0102xx, 0104xx, 500105, or 500106 or with durations less than 5 minutes are not eligible	
**Name**	**Description**		**File**
TRWBMODR	Well-being Module respondent		Respondent File
	**Edited Universe:**	All respondents	
	**Valid Entries:**	0	Did not respond to Well-being Module
		1	Responded to the Well-being Module
	***Note**	The 2021 Well-being Module was conducted from March 1, 2021 through December 31, 2021. All individuals on the Respondent file with TUMONTH > 02 were selected to be interviewed for the Well-being Module. Cases with TUMONTH = 01 or 02 will have values of -1 for TRWBMODR	
**Name**	**Description**		**File**
TUACTIVITY_N	Activity line number		Activity File,<br>Who File,<br>WB Activity File
	**Valid Entries:**	1<br>91	Min Value<br>Max Value
**Name**	**Description**		**File**
TUCASEID	ATUS Case ID (14-digit identifier)		All Files
**Name**	**Description**		**File**
TULINENO	ATUS person line number		ATUS-CPS File,<br>Respondent File,<br>Roster File,<br>Who File,<br>WB Respondent File,<br>EC Roster File
	**Valid Entries:**	1<br>30	Min Value<br>Max Value
	***Note**	The person selected to be interviewed for ATUS is always TULINENO = 1	

WECANTRIL	Edited: Please imagine a ladder with steps numbered from 0 at the bottom to 10 at the top. The top of the ladder represents the best possible life for you and the bottom of the ladder represents the worst possible life for you. If the top step is 10 and the bottom step is 0, on which step of the ladder do you feel you personally stand at the present time?		WB Respondent File
	**Edited Universe:**	All respondents	
	**Valid Entries:**	0<br>10	Min Value<br>Max Value
**Name**	**Description**		**File**
WEGENHTH	Edited: Would you say your health in general is excellent, very good, good, fair, or poor?		WB Respondent File
	**Edited Universe:**	All respondents	
	**Valid Entries:**	1	Excellent
		2	Very Good
		3	Good
		4	Fair
		5	Poor
**Name**	**Description**		**File**
WEHBP	Edited: In the last five years, were you ever told by a doctor or other health professional that you have hypertension, also called high blood pressure, or borderline hypertension?		WB Respondent File
	**Edited Universe:**	All respondents	
	**Valid Entries:**	1	Yes
		2	No
**Name**	**Description**		**File**
WEPAINMD	Edited: Did you take any pain medication yesterday, such as Aspirin, Ibuprofen or prescription pain medication?		WB Respondent File
	**Edited Universe:**	All respondents	
	**Valid Entries:**	1	Yes
		2	No

WEREST	Edited: When you woke up yesterday, how well-rested did you feel? Did you feel very rested, somewhat rested, a little rested, or not at all rested?		WB Respondent File
	**Edited Universe:**	All respondents	
	**Valid Entries:**	1	Very
		2	Somewhat
		3	A little
		4	Not at all
**Name**	**Description**		**File**
WETYPICAL	Edited: Thinking about yesterday as a whole, how would you say your feelings, both good and bad, compared to a typical [FILL= DAY]? Were they better than a typical [FILL=DAY], the same as a typical [FILL=DAY], or worse than a typical [FILL=DAY]?		WB Respondent File
	**Edited Universe:**	All respondents	
	**Valid Entries:**	1	Better
		2	The same
		3	Worse
	***Note**	The FILL is the day of the week about which the respondent was being interviewed. For example, if the diary day was a Tuesday, the respondent was asked how his feelings yesterday compared to a typical Tuesday.	
**Name**	**Description**		**File**
WRTELIG	Total time spent in all activities eligible to be selected for Well-being module		WB Respondent File
	**Edited Universe:**	All Well-being respondents	
	**Valid Entries:**	5<br>1440	Min Value<br>Max Value
	***Note**	Includes time spent in all activities with duration 5 minutes or more, except 0101xx, 0102xx, 0104xx, 500105, and 500106	
**Name**	**Description**		**File**
WUFINLWGT	Well-being module respondent weight		WB Respondent File
	**Valid Entries:**	0<br>9999999999	Min Value<br>Max Value
**Name**	**Description**		**File**
WUFNACTWT	Well-being module activity weight		WB Activity File

	**Valid Entries:**	0<br>99999999999	Min Value<br>Max Value
	***Note**	For each activity, if the data were missing, or the respondent refused to answer or reported "don't know" to 4 or more of the affect questions (WUHAPPY, WUPAIN, WUSAD, WUSTRESS, and WUTIRED), meaningful (WUMEANING), or interaction (WUINTERACT) question, a value of 0 was assigned to the activity weight for that activity.	

WUHAPORD	Order of WUHAPPY		WB Activity File
	**Valid Entries:**	1<br>5	Min Value<br>Max Value
	***Note**	For each respondent to the Well-being module, the order of the affect questions (WUHAPPY, WUPAIN, WUSAD, WUSTRESS, and WUTIRED) was assigned randomly.<br>For each WB respondent, the order of the affect questions was the same for each selected activity.	
**Name**	**Description**		**File**
WUHAPPY	From 0 to 6, where a 0 means you were not happy at all and a 6 means you were very happy, how happy did you feel during this time?		WB Activity File
	**Valid Entries:**	0<br>6	Min Value<br>Max Value
**Name**	**Description**		**File**
WUINTERACT	Were you interacting with anyone during this time, including over the phone?		WB Activity File
	**Valid Entries:**	1	Yes
		2	No
**Name**	**Description**		**File**
WUMEANING	From 0 to 6, how meaningful did you consider what you were doing?<br><br>0 means it was not meaningful at all to you and a 6 means it was very meaningful to you.		WB Activity File
	**Valid Entries:**	0<br>6	Min Value<br>Max Value
**Name**	**Description**		**File**
WUPAIN	From 0 to 6, where a 0 means you did not feel any pain at all and a 6 means you were in severe pain, how much pain did you feel during this time if any?		WB Activity File
	**Valid Entries:**	0<br>6	Min Value<br>Max Value
**Name**	**Description**		**File**
WUPNORD	Order of WUPAIN		WB Activity File
	**Valid Entries:**	1<br>5	Min Value<br>Max Value
	***Note**	For each respondent to the Well-being module, the order of the affect questions (WUHAPPY, WUPAIN, WUSAD, WUSTRESS, and WUTIRED) was assigned randomly.<br>For each WB respondent, the order of the affect questions was the same for each selected activity.	

Name	Description	File
WUSAD	From 0 to 6, where a 0 means you were not sad at all and a 6 means you were very sad, how sad did you feel during this time?	WB Activity File
	**Valid Entries:** 0 Min Value<br>6 Max Value	

Name	Description	File
WUSADORD	Order of WUSAD	WB Activity File
	**Valid Entries:** 1 Min Value<br>5 Max Value	
	***Note** For each respondent to the Well-being module, the order of the affect questions (WUHAPPY, WUPAIN, WUSAD, WUSTRESS, and WUTIRED) was assigned randomly. For each WB respondent, the order of the affect questions was the same for each selected activity.	

Name	Description	File
WUSTRESS	From 0 to 6, where a 0 means you were not stressed at all and a 6 means you were very stressed, how stressed did you feel during this time?	WB Activity File
	**Valid Entries:** 0 Min Value<br>6 Max Value	

Name	Description	File
WUSTRORD	Order of WUSTRESS	WB Activity File
	**Valid Entries:** 1 Min Value<br>5 Max Value	
	***Note** For each respondent to the Well-being module, the order of the affect questions (WUHAPPY, WUPAIN, WUSAD, WUSTRESS, and WUTIRED) was assigned randomly. For each WB respondent, the order of the affect questions was the same for each selected activity.	

Name	Description	File
WUTIRED	From 0 to 6, where a 0 means you were not tired at all and a 6 means you were very tired, how tired did you feel during this time?	WB Activity File
	**Valid Entries:** 0 Min Value<br>6 Max Value	

Name	Description	File
WUTRDORD	Order of WUTIRED	WB Activity File
	**Valid Entries:** 1 Min Value<br>5 Max Value	
	***Note** For each respondent to the Well-being module, the order of the affect questions (WUHAPPY, WUPAIN, WUSAD, WUSTRESS, and WUTIRED) was assigned randomly. For each WB respondent, the order of the affect questions was the same for each selected activity.	

Name	Description	File
WXCANTRIL	WECANTRIL: allocation flag	WB Respondent File

	***Note**	See introduction for allocation flag values.
**Name**	**Description**	**File**
WXGENHTH	WEGENHTH: allocation flag	WB Respondent File
	***Note**	See introduction for allocation flag values

WXHBP	WEHBP: allocation flag	WB Respondent File
	***Note**	See introduction for allocation flag values
**Name**	**Description**	**File**
WXPAINMD	WEPAINMD: allocation flag	WB Respondent File
	***Note**	See introduction for allocation flag values
**Name**	**Description**	**File**
WXREST	WEREST: allocation flag	WB Respondent File
	***Note**	See introduction for allocation flag values
**Name**	**Description**	**File**
WXTYPICAL	WETYPICAL: allocation flag	WB Respondent File
	***Note**	See introduction for allocation flag values.
